# A critical evaluation of systematic reviews assessing the effect of chronic physical activity on academic achievement, cognition and the brain in children and adolescents: a systematic review

**DOI:** 10.1186/s12966-020-00959-y

**Published:** 2020-06-22

**Authors:** Thomas M. Wassenaar, Wilby Williamson, Heidi Johansen-Berg, Helen Dawes, Nia Roberts, Charlie Foster, Claire E. Sexton

**Affiliations:** 1 Wellcome Centre for Integrative Neuroimaging, FMRIB, Nuffield Department of Clinical Neurosciences, University of Oxford, John Radcliffe Hospital, Headley Way, Oxford, OX3 9DU UK; 2 Trinity Institute of Neurosciences (TCIN), University of Dublin, College Green, Dublin 2, Ireland; 3 Department of Sport Health Sciences and Social Work, Centre for Movement Occupational and Rehabilitation Sciences, Oxford Brookes Centre for Nutrition and Health, Oxford Brookes University, Oxford, OX3 0BP UK; 4 Information Specialist Department, Bodleian Health Care Libraries, University of Oxford, Oxford, UK; 5 Centre for Exercise, Nutrition and Health Sciences, School for Policy Studies, University of Bristol, Queens Road, Bristol, BS8 1QU UK; 6 Wellcome Centre for Integrative Neuroimaging, Oxford Centre for Human Brain Activity, Department of Psychiatry, University of Oxford, Warneford Hospital, Oxford, OX3 7JX UK

**Keywords:** Physical activity, Cognitive function, Academic achievement, Brain imaging, MRI, Child, Adolescence, Policy

## Abstract

**Background:**

International and national committees have started to evaluate the evidence for the effects of physical activity on neurocognitive health in childhood and adolescence to inform policy. Despite an increasing body of evidence, such reports have shown mixed conclusions. We aimed to critically evaluate and synthesise the evidence for the effects of chronic physical activity on academic achievement, cognitive performance and the brain in children and adolescents in order to guide future research and inform policy.

**Methods:**

MedLine, Embase, PsycINFO, Cochrane Library, Web of Science, and ERIC electronic databases were searched from inception to February 6th, 2019. Articles were considered eligible for inclusion if they were systematic reviews with or without meta-analysis, published in peer-reviewed (English) journals. Reviews had to be on school-aged children and/or adolescents that reported on the effects of chronic physical activity or exercise interventions, with cognitive markers, academic achievement or brain markers as outcomes. Reviews were selected independently by two authors and data were extracted using a pre-designed data extraction template. The quality of reviews was assessed using AMSTAR-2 criteria.

**Results:**

Of 908 retrieved, non-duplicated articles, 19 systematic reviews met inclusion criteria. One high-quality review reported inconsistent evidence for physical activity-related effects on cognitive- and academic performance in obese or overweight children and adolescents. Eighteen (critically) low-quality reviews presented mixed favourable and null effects, with meta-analyses showing small effect sizes (0.1–0.3) and high heterogeneity. Low-quality reviews suggested physical activity-related brain changes, but lacked an interpretation of these findings. Systematic reviews varied widely in their evidence synthesis, rarely took intervention characteristics (e.g. dose), intervention fidelity or study quality into account and suspected publication bias. Reviews consistently reported that there is a lack of high-quality studies, of studies that include brain imaging outcomes, and of studies that include adolescents or are conducted in South American and African countries.

**Conclusions:**

Inconsistent evidence exists for chronic physical activity-related effects on cognitive-, academic-, and brain outcomes. The field needs to refocus its efforts towards improving study quality, transparency of reporting and dissemination, and is urged to differentiate between intervention characteristics for its findings to have a meaningful impact on policy.

## Introduction

Physical inactivity is an important risk factor for chronic diseases (e.g. cardiovascular disease, depression), obesity, and early deaths [1–4] placing a high economic burden on society [5]. Conversely, higher levels of physical activity (PA) have been associated with lower risk of mortality, beneficial mental- and cardiovascular health outcomes [6–8], and possibly improvements in cognitive and brain health [9]. Increasing PA has therefore been considered a low-cost strategy for global health improvement [10, 11], and has received great interest from scientific and public health communities demonstrated by the 2012 and 2016 Lancet series on PA (2012 series: https://www.thelancet.com/series/physical-activity, and 2016 series: https://www.thelancet.com/series/physical-activity-2016), the United States (US) 2018 Physical Activity Guidelines [12] and the 2018 World Health Organization (WHO) Global Action Plan for Physical Activity [13]. Specifically, both the US and WHO guidelines recommend 150 min of moderate intensity PA (MPA) or 75 min of vigorous intensity PA (VPA) per week for adults and at least 60 min of MVPA per day for children and adolescents (5–18 years), as well as muscle-strengthening activities. However, globally approximately 20–30% of adults [4, 11] and the majority of youth, including 80% of adolescents [11, 14], do not meet recommended levels of PA.

Childhood and adolescence is marked by rapid social, psychological and neurobiological development and provides the foundation for future health [15–17]. Consequently, scientists have begun to examine the effects of PA on brain structure and function, using neuroimaging tools such as magnetic resonance imaging (MRI) and electroencephalography (EEG), and cognition in this population [9]. The findings of these studies have been summarised extensively in systematic reviews, which have been evaluated by The 2018 Physical Activity Guidelines Advisory Committee (PAGAC) to inform policy [12]. The report suggested *moderate* evidence for PA-related beneficial effects on cognitive performance during pre-adolescence, but *inconsistent evidence* during adolescence [12]. These conclusions were recently updated to also suggest beneficial effects on brain structure and function during pre-adolescence and limited but promising evidence for PA on cognition in adolescence [9]. In contrast, the United Kingdom (UK) equivalent of the PAGAC concluded there was inconclusive evidence for effects of PA on cognitive and academic performance, but beneficial effects on maths performance [18].

Both of these reports were based on conclusions from a small, non-overlapping set of systematic reviews (US: 9 and UK: 2) and did not provide insight into review quality, nor quality of the primary studies on which the reviews’ conclusions were based. A recent systematic review of reviews considered findings from 25 reviews and concluded that the evidence supports a causal link between PA and cognition in young people [19]. However, the authors did not incorporate review quality in their evidence synthesis and based their conclusions on a subset of reviews that included a mixture of observational and interventional evidence. To seek clarity among the contrasting reports, provide specific recommendations for the field, and inform policy, this systematic review of reviews aimed to synthesise the evidence for the effect of PA or exercise on brain structure and function, academic-, and cognitive performance in childhood and adolescence. In this review we aimed to include overall reporting of bias and heterogeneity in the literature, the quality of the primary studies and reporting of intervention fidelity, as well as the consistency of conclusions, limitations and recommendations across the literature.

## Method

This systematic review was registered at PROSPERO (ID: CRD42019124472) and conducted in accordance with the Preferred Reporting Items for Systematic Reviews and Meta-analysis (PRISMA) guidelines (checklist is provided in Additional file 1) [20].

### Search strategy and selection criteria

An information specialist (NR) performed an electronic search of the following databases, originally on June 14th 2018, and updated on February 6th 2019: MedLine, Embase, PsycINFO, Cochrane Library, Web of Science and ERIC. No date restrictions were applied to the initial search. Reference lists of identified articles were examined for additional relevant articles. Data sources and (medical subject headings) search terms are provided in Additional File 2. In line with the a priori defined selection criteria (PROSPERO, ID: CRD42019124472), systematic reviews with or without meta-analysis that were published in English with clearly defined inclusion criteria were included if they reported on the effects of chronic PA interventions (i.e. more than one PA session, over a set period), including randomised controlled trials (RCT), quasi-experimental studies, controlled and pre-post designs, on cognitive, academic and /or brain MRI outcomes in school-aged children or adolescents. Systematic reviews that contained a mixture of observational and interventional evidence, that reported on a single bout of acute PA (i.e. a single session) or cardiovascular fitness only, or included case reports, were excluded.

The in- and exclusion criteria were adapted post-protocol to exclude systematic reviews that also included observational studies because the findings from interventional and observational studies were generally combined in the evidence synthesis. It was deemed too subjective to extract conclusions regarding the effects of intervention studies only. Studies that distinguished between multicomponent (e.g. PA and diet) and PA only interventions were included, but only results from PA only interventions were considered in this systematic review.

Systematic reviews that also included findings of acute PA interventions were considered for inclusion only if they selectively reported on the effects of chronic PA interventions. Following data extraction, it became clear that no single systematic review had aimed to include MRI studies only. Instead, two out of four systematic reviews included studies that used either MRI or EEG. We therefore decided to adapt our inclusion criteria to also include brain EEG markers as an outcome measure. Because the search criteria included the term MRI, we post-hoc searched the databases (October 1st, 2019) for systematic reviews that included EEG studies. No additional systematic reviews were found.

### Review selection and data extraction

Following removal of duplicates, two reviewers (TW, WW) independently screened all titles and abstracts using Abstrackr [21]. Short-listed full-text reviews were then independently assessed using the in- and exclusion criteria. Any disagreements between the authors were discussed with two other authors (CS and CF) and resolved by consensus. A data extraction form was piloted independently by two authors (TW, WW) and adjusted to ensure it captured all relevant data, including: year of publication, type of review (systematic review with or without meta-analysis), review methodology (aim, in/exclusion criteria, number of studies included/excluded, database search, quality and bias assessment method), characteristics of included studies (design, number of included participants, type of participants and countries), outcome measures, reporting on intervention fidelity, limitations and recommendations. A single author extracted the data from selected studies using the data extraction template which were verified by a second author and disagreements were resolved by consensus.

### Quality assessment

The methodological quality of all reviews was assessed using the updated AMSTAR checklist, AMSTAR-2 [22]. Two reviewers (TW, WW) independently scored the selected reviews. The quality of each review is reflected by an overall confidence rating, which is determined by an evaluation of non-critical and critical domains (seven critical items: preregistration, literature search, justification for excluding studies, risk of bias assessment, appropriateness of meta-analytical methods, risk of bias in synthesis of results, publication bias). A lack of addressing one or multiple critical domains resulted in a, respectively, low or critically-low confidence rating. If no critical flaws were present, the presence of *non-critical* weaknesses determined whether the review received a high (no weaknesses) or moderate (one or more weaknesses) confidence rating. While it is not recommended to combine individual item ratings into an overall score [22], we provided the total score to merely acknowledge that a gradient exists between reviews in their study quality.

## Results

### Search results

**Fig. 1:**
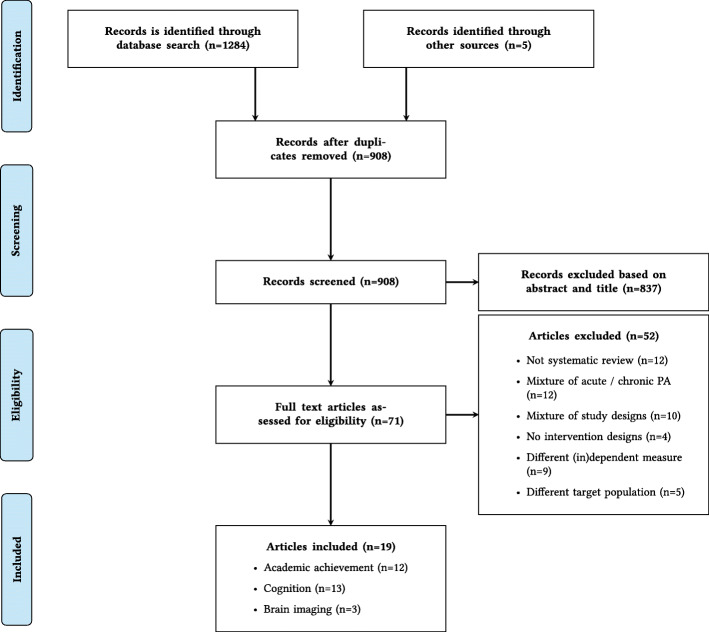
PRISMA flow diagram

Of 908 abstracts that were screened, 71 full-text articles were reviewed. Only 19 articles met inclusion criteria (Fig. 1). An overview of excluded studies, with reasons, is provided in Additional file 3. Twelve articles reviewed academic outcomes [23–34], thirteen reviewed cognitive outcomes [24, 25, 27, 29–31, 33, 35–40], and three evaluated brain outcomes [27, 39, 41]. Seven systematic reviews included randomised controlled trials (RCT) only, and the remainder included a combination of intervention studies (including RCTs, quasi-experimental studies and controlled designs). Eight systematic reviews (42%) also included a meta-analysis. An overview of search details of each systematic review is provided in Additional file 4.

**Fig. 2:**
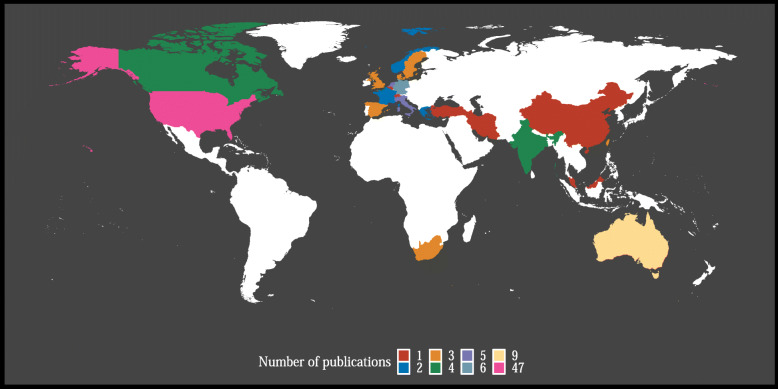
Countries where PA interventions have been conducted. PA interventions included in the 19 systematic reviews have been conducted in 26 countries. The findings for these interventions have been presented in 118 unique publications, the majority (40%) of which were conducted in the USA

The 19 systematic reviews included a total of 118 unique *primary studies*, of which 84 on cognitive outcomes, 53 on academic outcomes and nine on brain outcomes. The research was predominantly conducted in developed countries (Fig. 2, an overview of countries per outcome is provided in Additional file 5), especially in the USA and Australia. None of the primary studies were conducted in countries in South-America or Africa, with the exception of South Africa. The quality of the primary studies was generally regarded to be low to moderate (details of assessments per review are provided in Additional file 6).

### Review quality

Of 19 systematic reviews, one received a high confidence rating, one a low confidence rating and 17 a critically low confidence rating (i.e. did not address multiple critical domains, as defined in quality assessment section above; see Table 1, and details in Additional file 7). While 18 reviews performed a comprehensive search (95%) and provided study characteristics (95%), only five (26%) had an a-priori design, three (16%) included an overview of excluded studies, 14 assessed risk of bias (72%), seven of eight (88%) performed a meta-analysis with generally appropriate methods (i.e. used accepted statistical techniques for combination of results and exploration of causes of heterogeneity), and three (of 15 that performed a quality assessment: 20%) considered quality information in their evidence synthesis.

**Table 1:** Descriptors of systematic reviews

Authors	Population^a^	Number and design of relevant studies	Author’s conclusions	AMSTAR-2 rating^b^
Álvarez-Bueno et al. [23]	Healthy children and adolescents (4–13 years)	26 intervention studies (8 non-RCT)	PA benefits several aspects of academic achievement, particularly maths, reading and composite scores	Critically low (10.5)
Álvarez-Bueno et al. [38]	Healthy children and adolescents (4–18 years)	36 intervention studies (5 non-RCT)	PA benefits several domains of non-executive, executive and meta-cognitive functions and skills, with curricular PE interventions being most effective	Critically low (10.5)
Bustamante, Williams, and Davis [39]	Overweight or obese children and/or adolescents	14 intervention studies (5 non-RCT)	Positive effects on cognitive and neurologic outcomes in high-quality RCTs, but all studies showing neurologic benefits were from the same group	Critically low (4.5)
de Greeff et al. [24]	Primary school children (6–12 years)	14 intervention studies (3 non-RCT)^c^	Positive effects were found for physical activity on executive functions, attention and academic performance; largest effects are expected for interventions that aim for continuous regular physical activity over several weeks	Critically low (8)
Gunnell et al. [27]	Healthy children (1–17.99 years)	49 RCT^c^	PA is unrelated or beneficial for cognitive function (incl. academic achievement), brain function and brain structure	Critically low (8)
Haapala [28]	Healthy children and adolescents (7–16 years)	4 RCT^c^	Review does not support the idea that PA interventions are effective at enhancing academic performance; short intervention times (less than 36 and 64 weeks) have little effect.	Critically low (3.5)
Jackson et al. [40]	Healthy children (7–12 years)	8 RCT	Increased regular physical activity is associated with a small and measurable improvement in neuropsychological tests of executive functions, specifically inhibitory control	Critically low (6.5)
Lees and Hopkins [29]	Children and adolescents (< 19 years)	4 RCT	PA is positively associated with cognition and academic achievement	Critically low (4.5)
Li et al. [30]	Healthy adolescents (13–18 years)	2 intervention studies (1 non-RCT)^c^	PA effect on cognitive and academic performance is equivocal and limited in quantity and quality	Critically low (5.5)
Lubans et al. [41]	Children (7–11 years)	6 RCT (3 unique studies)	There is a lack of available evidence regarding neurobiological mechanisms	Critically low (5.5)
Martin et al. [31]	Overweight or obese children (3–18 years)	8 RCT	High-quality evidence for composite executive functions, but not academic achievement, attention, cognitive flexibility or inhibition control; however, this evidence is based on a small number of studies	High (16)
Martin and Murtagh [32]	Children (5–12 years)	4 intervention studies(2 non-RCT)	All of the studies (s = 4) reported some positive effects of physically active academic lessons on learning outcomes	Critically low (5.5)
Mura et al. [33]	Healthy children (3–18 years)	28 intervention studies (7 non-RCT)	Positive effects of PA interventions on academic achievement and cognitive performance	Critically low (3.5)
Pucher, Boot, and Vries [34]	School-aged children	4 intervention studies (3 non-RCT)	No negative effects of PA on academic performance and some positive effects	Critically low (5.5)
Singh et al. [25]	Healthy children and adolescents (3–16 years)	11 high-quality intervention studies (3 non-RCT; out of 58 interventions)	Inconclusive evidence for beneficial effects of PA on cognitive or academic performance, but strong evidence for beneficial effects on maths performance	Low (10.5)
Spruit et al. [26]	Children and adolescents (mean age 11–18)	10 intervention studies (3 non-RCT), including dissertations	PA interventions are effective in improving academic performance	Critically low (4.5)
Suarez-Manzano et al. [36]	Children and adolescents with ADHD (6–18 years)	7 intervention studies (1 non-RCT)	Systematic PA (≥ 30 min per day, ≥ 40%, intensity, ≥ three days per week, ≥ five weeks) further improves attention and inhibition	Critically low (4.5)
Vazou et al. [35]	Typically developing children and adolescents (4–16 years)	27 intervention studies (3 non-RCT)	PA interventions have a positive impact on cognition, but more research is needed	Critically low (3.5)
Verburgh et al. [37]	Children and adolescents (6–17), but one study in young adults	5 RCT^c^	Inconsistent results regarding the effects of exercise on executive functions	Critically low (7.5)

Abbreviations: *PA* physical activity, *PE* physical education, *RCT* randomised controlled trial

^a^Age range taken from inclusion criteria unless a more specific range was provided

^b^The AMSTAR-2 confidence rating (critically low, low, medium or high) is reported, followed by the overall score. The overall score is added to acknowledge the inter-review variability in quality, but is not used in the synthesis of findings as recommended by Shea et al. [22]

^c^This review also includes acute PA studies which have been excluded from this count

### Outcomes

#### Academic achievement

Twelve systematic reviews evaluated academic outcomes (Table 2), one of which synthesised evidence from overweight/obese children and adolescents [31]. Reviews included between one [30] and 26 primary studies [23] and 19 studies (36%) were included in more than one review (Additional file 8). Four reviews included a meta-analysis, and reviews synthesised findings of studies in terms of overall academic achievement, its sub-domains (e.g. maths, language, reading), or both.

**Table 2:** Academic outcomes: findings from systematic reviews and meta-analyses (ordered by quality rating)

Authors	Population	Systematic review results	Meta-analysis results^c^
**High-quality reviews**			
Martin et al. [31]	Overweight or obese children (3–18 years)	No effects of PA on maths, reading or language were found (moderate quality evidence) **Maths (s = 3):** no evidence for an effect **Reading (s = 2):** no evidence for an effect **Language (s = 2):** no evidence for an effect	**Maths:** 0.49 (−0.04, 1.01), I^2^ =57% (s = 2) **Reading:** 0.10 (−0.30, 0.49), I^2^ =63% (s = 2) **Language:** not performed
**Low-quality reviews**			
Singh et al. [25]	Children and adolescents (3–16 years)	7 high-quality studies: 15/25 analysed constructs (60%) found a beneficial effect, leading to inconclusive evidence; no studies reported adverse effects of PA on academic achievement **Maths:** Strong evidence for PA on maths performance (86% of outcomes are beneficial) **Language**: Inconclusive evidence for language performance (27% of outcomes are beneficial)	NA
**Critically low-quality reviews**			
Álvarez-Bueno et al. [23]	Healthy children and adolescents (4–13 years)	**Language:** 4/9 studies reported significant improvements in the intervention group **Maths:** 13/18 studies reported significant improvements in the intervention group **Reading:** 5/10 studies reported significant improvements in the intervention group **Composite scores:** 2/5 studies reported significant differences between the groups **Other subjects:** 1/3 studies reported improvements after the PA intervention	**Language:***d* =0.16 (−0.06, 0.37), I^2^ =71.7% (s = 3, k = 7) **Maths:***d* =0.21 (0.09, 0.33), I^2^ =57.8% (s = 10, k = 16)^b^ **Reading:***d* =0.13 (0.02, 0.24), I^2^ =25.5% (s = 5, k = 10) **Composite scores:***d* =0.26 (0.07, 0.45), I^2^ =75.6% (s = 4, k = 8)
de Greeff et al. [24]	Primary school children (6–12 years)	Academic achievement: 9/14 reported positive findings on at least 1 outcome measure, 5 reported no significant findings	**Academic performance:***g* =0.26 (0.02, 0.49), I^2^ =39% (k = 4, s = 3) **Maths:***g* =0.09 (−0.17, 0.35), I^2^< 0.01% (s = 1, k = 2) **Reading:***g* =0.15 (−0.15, 0.46), I^2^ =35.31% (s = 2, k = 2) **Spelling:***g* =0.34 (−0.23, 0.92), I^2^ = NA (s = 1, k = 1)
Gunnell et al. [27]^a^	Healthy children (1–17.99 years)	Academic achievement and intelligence: mixed evidence. *PA* vs *none*^*a*^*(n = 2202 participants, s = 9):* 5/9 —, 1/9 ↑, 3/9 — ↑ *Multiple comparisons (n = 1141, s = 4):* 3/4 ↑, 1/4 — ↑↓ *PA* vs *PA (n = 546, s = 5):* 3/5 ↑, 1/5 — ↑, 1/5 —	Not performed given heterogeneity of study designs, PA exposures and outcomes
Li et al. [30]	Healthy adolescents (13–18 years)	1/1 studies showed a beneficial effect on academic performance. Of two parameters, only one showed significance	NA
Martin and Murtagh [32]	Children (5–12 years)	4/4 reported some positive effects	NA
Pucher, Boot, and Vries [34]	School-aged children	Across 4 studies: additional PA is not likely to affect academic performance negatively, and positive effects of PA have been demonstrated and are more likely when PE is delivered at vigorous levels and by a trained specialist/teacher	NA
Lees and Hopkins [29]	Children and adolescents (< 19 years)	3/3 showed positive effects on academic performance	NA
Spruit et al. [26]	Children and adolescents (mean age 11–18)	Physical activity interventions are effective in improving academic achievement (s = 10)	**Academic performance:***d* =0.367 (0.038, 0.69), (s = 10, k = 34)
Mura et al. [33]	Children (3–18 years)	10/16 studies showed an improvement in academic performance (maths (s = 4), reading (s = 1), overall academic achievement (s = 5)), in 6/16 it did not worsen academic performance	NA
Haapala [28]	Children and adolescents (7–16 years)	Positive effect of PA on maths, reading and language skills in 3/4 studies. In 2/4 studies no significant differences between groups	NA

Abbreviations: *d* = Cohen’s *d*, *ES* effect size, *g* = Hedges’ *g*, *k* number of comparisons, *n* number of participants, *NA* not assessed, *PA* physical activity, *RCT* randomised controlled-trial, *s* number of study/studies

^a^*PA* vs *none:* PA was compared to a sedentary control condition, *Multiple comparisons:* studies with multiple treatment and/or control groups, *PA* vs *PA:* comparison of multiple types of PA interventions. Coding represents combinations of: — = null results, ↓ = unfavourable results, ↑ = favourable results

^b^Effect size changes in sensitivity analysis, the findings of which are presented in Additional file 9

^c^Results are reported as: standardized mean difference, 95% confidence intervals, heterogeneity statistics if available, the number of studies (s) and number of comparisons (k)

Across all twelve reviews, six concluded that PA benefits academic performance [23, 24, 26, 29, 32, 33], and the remaining six concluded that there was mixed or inconclusive evidence for PA-related academic changes (Tables 1 and 2) [25, 27, 28, 30, 31, 34].

Only one review received a high confidence rating [31]. Among three RCTs of obese/overweight children, this review found no evidence for PA-related improvements in maths, reading or language performance. Sensitivity analyses for risk of bias (e.g. attrition) and cluster RCT designs were performed, but were less meaningful due to a small number of studies.

Among the 11 low- to critically low- quality reviews (i.e. lack of addressing respectively one versus multiple critical domains), six reviews found (mainly) positive effects of PA on academic performance and five presented mixed evidence and conclusions. All three meta-analyses showed a small beneficial effect of PA on overall academic performance (effect sizes are presented in Table 2), but only one (of two) found significant evidence for small positive effects on its sub-domains [23]. This meta-analysis included 26 intervention studies (RCT and quasi-experimental) and found evidence for beneficial effects of PA on maths, reading and composite scores, albeit with substantial heterogeneity among studies (I^2^ > 50%, apart from reading). Sensitivity analyses showed changes in effect sizes of reading-, language-related skills and composite scores after removal of studies, and a drop in effect size for maths performance (from *d* = 0.21 to *d* =0.12) upon exclusion of low quality quasi-experimental studies. Among the other two meta-analyses, one included three studies [24], whereas the other included a large number of non-peer reviewed dissertations [26].

Subgroup analyses of meta-analyses (Additional file 9) further suggest that academic performance may benefit most from PA during curricular PE [23] and cognitively challenging PA [24, 26]. Intervention duration does not seem to be an important moderator of PA-related academic changes [23, 24, 26].

In summary, evidence from systematic reviews is inconsistent, with conclusions suggesting positive as well as mixed (inconclusive) effects of PA on overall academic achievement and its sub-domains. One high confidence review on overweight and obese children did not report beneficial effects of PA on academic performance [31].

#### Cognitive function

Thirteen systematic reviews evaluated cognitive outcomes, one of which synthesised evidence from children with ADHD [36] and two evaluated findings from overweight/obese children [31, 39]. Reviews included between one [29] and 36 intervention studies [27, 38], and of 83 published studies, 42 (50%) were included in more than one review (Additional file 8). Six reviews included a meta-analysis and reviews synthesised findings of studies in terms of overall cognitive performance, and / or various sub-domains (e.g. executive functions, memory).

Across all thirteen reviews, eight concluded that PA was beneficial [24, 29, 33, 35, 36, 38–40] and five reported mixed or inconclusive evidence [25, 27, 30, 31, 37]. None of the reviews suggested worse cognitive performance following PA (Tables 1 and 3).

**Table 3:** Cognitive outcomes: findings from systematic reviews and meta-analyses (ordered by quality rating)

Authors	Population	Systematic review results	Meta-analysis results^c^
**High-quality reviews**			
Martin et al. [31]	Overweight or obese children (3–18 years)	High quality evidence for an effect of PA on composite executive functions and non-verbal memory, but not cognitive flexibility, inhibition (low quality), attention or visuo-spatial abilities **Composite executive functions (s = 3):** 2/3 no evidence for an effect, 1/3 showing a positive effect **Inhibition control (s = 1)**: no evidence for an effect **Attention (s = 3):** no evidence for an effect **Working memory (s = 1):** no evidence for an effect **Visuo-spatial abilities (s = 3):** no evidence for an effect **Cognitive flexibility (s = 2):** no evidence for an effect **Non-verbal memory (s = 2):** some evidence for a small effect **General intelligence (s = 1):** some evidence for an effect	**Composite executive functions:***PA:* 0.42 (0.05, 0.78), (s = 1); *Exergaming*: 0.58 (−0.02, 1.18), (s = 1) **Inhibition control:** not performed **Attention:** 0.46 (−0.16, 1.08), I^2^ =41% (s = 2) **Working memory:** not performed **Visuo-spatial abilities:** not performed **Cognitive flexibility:** −0.06 (−0.37, 0.25), I^2^ =0% (s = 2) **Non-verbal memory:** not performed **General intelligence:** not performed
**Low-quality reviews**			
Singh et al. [25]	Children and adolescents (3–16 years)	6 high-quality studies: 10/21 (48%) analyses found a significant beneficial intervention effect, leading to inconclusive evidence	NA
**Critically low-quality reviews**			
Álvarez-Bueno et al. [38]^a^	Healthy children and adolescents (4–18 years)	**Non-executive functions:** 7/7 found improvements; of 4 studies that included multiple intervention groups, two suggested that increases in duration and intensity were associated with greater improvements **Executive functions:** 29/29 found improvements; of 11 studies that included multiple intervention groups, three did not find differences between the groups **Meta-cognition:** 15/15 found improvements; of 6 studies that included multiple intervention groups, none found differences in improvements	**Non-executive functions:***d* =0.23 (0.09, 0.37), I^2^ =21.9% (s = 6, k = 17) **Executive functions:***d* =0.20 (0.10, 0.30), I^2^ =70.0% (s = 22, k = 42) **Working memory:***d* =0.14 (0.00, 0.27), I^2^ =48% (s = 9, k = 13) **Selective attention / inhibition:***d* =0.26 (0.10, 0.41), I^2^ =76.0% (s = 17, k = 24) **Selective attention:***d* =0.13 (−0.07, 0.33), I^2^ =66.8% **Inhibition:***d* =0.38 (0.13, 0.63), I^2^ =68.7% **Cognitive flexibility:***d* =0.11 (−0.10, 0.32), I^2^ =68.7% (s = 4, k = 5) **Meta-cognition:***d* =0.23 (0.13, 0.32), I^2^ =4.7% (s = 10, k = 21) **Higher level executive functions:***d* =0.19 (0.06, 0.31), I^2^ =12.9% (s = 7, k = 13)
de Greeff et al. [24]	Primary school children (6–12 years)	Combined academic achievement and cognition: 9/14 reported positive findings on at least 1 outcome measure, 5 reported no significant findings	**Overall cognitive functions:***g* =0.37 (0.20, 0.55), I^2^ =64.92% (s = 14, k = 18) **Executive functions:***g* =0.24 (0.09, 0.39), I^2^ = 34% (s = 12, k = 15) **Working memory:***g* =0.36 (0.10, 0.62), I^2^ =56.79% (s = 6, k = 8) **Cognitive flexibility:***g* =0.18 (0.01, 0.35), I^2^ =4.79% (s = 4, k = 4) **Inhibition:***g* =0.19 (−0.04, 0.42), I^2^ =49.7% (s = 6, k = 7) **Planning:***g* =0.12 (−0.08, 0.32), I^2^< 0.01% (s = 4, k = 4)
Gunnell et al. [27]^b^	Healthy children (1–17.99 years)	**Inhibitory control:***PA* vs *none (n = 1248, s = 5):* 3/5 —, 1/5 — ↑, 1/5 — ↓; *PA* vs *PA (n = 557, s = 6):* 3/6 ↑, 1/6 —, 2/6 — ↑; *Multiple comparisons (n = 181, s = 1):* 1/1 — **Working memory:***PA* vs *none (n = 1804, s = 3):* 1/3 —, 1/3 ↑, 1/3 —↑; *PA* vs *PA (n = 487, s = 3):* 2/3 —, 1/3 ↑; *Multiple comparisons (n = 181, s = 1):* 1/1 — **Cognitive flexibility:***PA* vs *PA (n = 501, s = 2):* 1/2 —↑, 1/2 ↑; *Multiple comparisons (n = 246, s = 2):* 1/2 —, 1/2 — ↑ **Unitary constructs:***PA* vs *none (n = 549, s = 2):* 1/2 — ↑, 1/2 —; *PA* vs *PA (n = 472, s = 3):* 1/3 ↑, 1/3 — ↑, 1/3 — **Attention:***PA* vs *none (n = 1809, s = 8):* 5/8 —, 1/8 ↑, 1/8 — ↑; *PA* vs *PA (n = 156, s = 5):* 1/5 — ↑, 2/5 ↑, 2/5 —; *Multiple comparisons (n = 757, s = 1):* 1/1 — ↑ **Information processing:***PA* vs *none (n = 1659, s = 5):* 1/5 ↑, 4/5 —; *Multiple comparisons (n = 265, s = 2):* 2/2 — ↑; *PA* vs *PA (n = 448, s = 3):* 3/3 — **Memory:***PA* vs *none (n = 44, s = 1):* 1/1 — **Motor speed and learning:***PA* vs *PA (n = 508, s = 2):* 1/2 ↑, 1/2 — **Composite cognition:***PA* vs *none (n = 1794, s = 3):* 1/3 —, 2/3 — ↑	Not performed given heterogeneity of study designs, PA exposures and outcomes
Verburgh et al. [37]	Children and adolescents (6-17 years), but one study in young adults	Inconsistent results among 5 studies that reported on the effect of chronic PA on executive functions (one in young adults)	**Executive functions** across age groups: *d* =0.14 (−0.04, 0.32), *Q* =5.1 (s = 5) **Planning:***d* = 0.16 (-0.07, 0.39), Q = 0.89 (s=3)
Jackson et al. [40]	Healthy children (7–12 years)	8/8 studies showed a positive effect of PA on inhibitory control, but none were statistically significant in isolation; other domains of executive function were measured too infrequently to perform a meta-analysis	**Inhibitory control:***d* =0.2 (0.03, 0.37), I^2^ =0%
Li et al. [30]	Healthy adolescents (13–18 years)	1/2 studies showed a beneficial effect on cognitive function; of five cognitive function parameters, only one showed significance	NA
Lees and Hopkins [29]	Children and adolescents (< 19 years)	1/1 studies showed positive effects of PA on cognitive performance; another study was included in the data table, but not part of the results section or evidence synthesis	NA
Bustamante, Williams, and Davis [39]	Overweight or obese children and/or adolescents	*Quasi-experimental (s = 4):* each of the four studies showed some benefit on neural, cognitive or academic outcomes; but the PA was confounded with other elements of the intervention and no control groups were present, thus providing little evidence for PA effects *RCT (s = 10, but only 5 independent studies):* high quality RCT’s (s = 2) have shown benefits for different executive functions	NA
Suarez-Manzano et al. [36]	Children and adolescents with ADHD (6–18 years)	7/7 studies showed a positive effect of PA on cognition, no study revealed a negative association; the systematic practice of PA between 5-20 weeks, 30-90mins at moderate-vigorous intensity (40-75%) produces a chronic effect that improves cognition in young people with ADHD	NA
Mura et al. [33]	Children (3–18 years)	7/9 studies showed an improvement in global cognitive performance, 1/9 showed no difference and 1/9 worse intelligence; two of these studies found dose-response relationships, with high dose PA performing better than low-dose PA or control; specific cognitive skills improved in almost all studies (6 studies)	NA
Vazou et al. [35]	Typically developing children and adolescents (4–16 years)	**Aerobic only (s = 7):** Significant cognitive outcomes: planning (s = 1), creativity (s = 2), working memory and spatial memory span (s = 4), attentional accuracy and spatial inattention (s = 1), cued recall memory (s = 1) and mathematics fluency (s = 1) **Motor skills (s = 4):** Improvements in working memory (s = 1), spatial processing/math/reading/concentration (s = 1 study), lower error on attentional task (s = 1), mixed effects (s = 1) **Cognitively engaging PA (s = 2):** Improved planning (s = 1), and spatial memory, but not verbal memory (s = 1) **Aerobic and motor skill (s = 1):** No difference in inhibition accuracy or reaction time (s = 1) **Motor skills and cognitively engaging PA (s = 7):** Improved inhibition (s = 1), attention related to arithmetic (s = 1), parent-rated inhibitory behavioral control and reaction time (s = 1), inhibition (s = 1), attention (s = 1), inattention and hyperactivity (s = 1) and attentional accuracy (s = 1) **Aerobic and cognitively engaging PA (s = 8):** Improvements in math fluency (s = 2), math and spelling (s = 1), fluid intelligence (s = 1), cognitive shifting (s = 1), memory recall (s = 1), verbal working memory and inhibition (s = 1), time-on-task in classroom (s = 1) **Aerobic, motor and cognitively engaging PA (s = 1):** Improved receptive attention (s = 1)	**Overall cognitive function:***g* =0.46 (0.28, 0.64), I^2^ =85% (s = 21, k = 28) **PA interventions versus comparison treatments:** *PA* vs *no treatment:* 0.86 (0.18, 1.55), I^2^ = 93% (s = 5) *PA* vs *academic instruction:* 0.57 (0.32, 0.83), I^2^ =81% (s = 10) *PA* vs *traditional PE:* 0.09 (−0.07, 0.24), I^2^ = 44% (s = 9) *PA combinations* vs *aerobic PA*: 0.80 (−0.08, 1.67), I^2^ =88% (s = 4) **Qualitatively different PA interventions vs comparison:** see subgroup analysis in Additional file 9

Abbreviations: *d* = Cohen’s *d*, *g* = Hedges’ *g*, *k* number of comparisons, *n* number of participants, *NA* not assessed, *PA* physical activity, *RCT* randomised controlled-trial, *s* number of study/studies

^a^This review also analysed *cognitive life skills*, which is different from any of the other typically examined cognitive functions and therefore excluded from this table

^b^PA vs none: PA was compared to a sedentary control condition, Multiple comparisons: studies with multiple treatment and/or control groups, PA vs PA: comparison of multiple types of PA interventions. Coding represents combinations of: —= null results, ↓ = unfavourable results, ↑ = favourable results

^c^Results are reported as: standardized mean difference, 95% confidence intervals, heterogeneity statistics if available, the number of studies (s) and number of comparisons (k)

One review received a high confidence rating [31]. Among seven RCTs of obese/overweight children, this review reported a significant PA-related benefit on non-verbal memory and composite executive functions based on findings from single studies, but not inhibition control, attention, working memory, cognitive flexibility or visuo-spatial abilities.

Among 12 (of 13) low- [25] to critically low- quality reviews, six reviews found positive effects of PA on cognitive performance and/or its sub-domains in healthy young people [24, 29, 33, 35, 38, 40], and four presented mixed evidence and conclusions [25, 27, 30, 37]. Two meta-analyses showed a positive effect of PA on overall cognitive performance [24, 35], two of three showed PA-related benefits on overall executive functions [24, 37, 38] and inhibition [24, 38, 40], and two of three meta-analyses reported non-significant effect sizes for planning or higher-level cognitive functions [24, 37]. Moreover, a meta-analysis additionally reported beneficial effects of PA on non-executive functions and working memory, but not selective attention or cognitive flexibility [38]. Despite some positive results, the effect sizes are generally small (0.1–0.3) and suffer from substantial heterogeneity. Moreover, only two of five meta-analyses performed a sensitivity analysis [35, 38], showing a lower effect size for overall cognitive functions following removal of outliers [35] and a change in effect size for working memory upon removal of studies [38]. There is some evidence for beneficial effects of PA on cognitive functions in children with overweight and obesity [39] and with ADHD [36].

Subgroup analyses of meta-analyses further suggest that cognitive performance may benefit most from PA during curricular PE [38], with mixed evidence for enhanced (i.e. quantitative increase in PA) or enriched PA (i.e. qualitative manipulation of PA, e.g. increasing coordinative task requirements; Additional file 9) [35, 38]. Intervention duration does not seem to be an important moderator of PA-related cognitive changes [24, 37, 40], although one review reported a negative relationship with working memory [38]. No significant relationships were found between study quality and heterogeneity [38] or effect sizes [37].

In summary, evidence from predominantly low-quality systematic reviews is inconsistent, with conclusions suggesting both positive and mixed (inconclusive) effects of PA on overall cognitive performance and its sub-domains. A single high-quality review [31] showed that PA may benefit executive functions and non-verbal memory of obese/overweight children, but evidence is based on findings from single studies. A comparison between reviews is complicated by the considerable variability in reporting of findings across systematic reviews (e.g. overall cognition vs sub-domains, choice of sub-domains).

#### Brain structure and function

While four systematic reviews reported on the effects of PA on the brain [27, 33, 39, 41], only three of these systematically explored exercise-related brain changes [27, 39, 41], the findings of which are discussed here. Reviews included five or six primary studies, and three (of 10 unique publications) were included in all reviews (Additional file 8) [42–44]. All reviews were classified as critically low quality. Across three reviews, one concluded that PA is beneficial for the brain [39], one reported mixed favourable and null effects [27] and one reported a lack of available evidence (Table 4) [41].

**Table 4:** Brain outcomes: findings from systematic reviews and meta-analyses

Author	Population	Systematic review results	Meta-analysis results
**Critically low-quality reviews**			
Gunnell et al. [27]	Healthy children (1–17.99 years)	For brain function, increases, no changes or a mixture were interpreted as being supportive of brain function; for brain structure, results were favourable or null **Brain function**^**a**^**:** *PA* vs *none: Activation (n = 86, s = 3):* 1/3 no change, 2/3 increased, decreased and no change ([study 1] decreased during anti-saccade task, increased or no change during flanker task; [study 2] no change in frontal or supplementary eye fields, increased in bilateral prefrontal cortex and decreased in bilateral parietal cortex) *Resting-state synchrony (n = 37, s = 1):* 1/1 increases, decreases and no change *Blood flow (n = 30, s = 1):* 1/1 no changes in cerebral blood flow velocity **Brain structure (*n*** **= 36, s = 2, same sample):** *PA* vs *none:* 1/2 improved white matter coherence and myelination, 1/2 no change	NA
Bustamante, Williams, and Davis [39]	Overweight or obese children and/or adolescents	Benefits for neurologic outcomes following PA in high quality studies (RCT, s = 4, 2/4 brain function, 2/4 brain structure), but all from the same group; results from a quasi-experimental study (s = 1) suggest a neural benefit, but the study is of low rigor and suffers from confounding	NA
Lubans et al. [41]^b^	Children (7–11 years)	5/6 studies reported significant brain changes (2/6 using EEG, 4/6 using MRI one of which explored brain structure), but there was little overlap between studies	NA

Abbreviations: n = number of participants, *NA* not assessed, PA = physical activity, RCT = randomised controlled-trial, s = study/studies

^a^The authors also included findings on changes in brain-derived neurotrophic factor, which are not measured by EEG or MRI and therefore excluded from this table. PA vs none: PA was compared to a sedentary control condition

^b^This study examined brain changes as potential mediators of cognitive changes, rather than exploring brain changes per se

Among healthy children and adolescents, Gunnell et al. [27] stratified findings from six RCTs by brain function and structure and showed some evidence for changes in activation and resting-state synchrony following a PA intervention, but not blood flow and inconsistent changes in white matter structure. Lubans et al. [41] tabulated brain changes from six studies to explore neurobiological mechanisms of cognitive changes, and showed increases, decreases or no changes following PA interventions in widespread brain areas (as well as cognitive changes). The authors did not synthesise brain findings and reported a lack of overlap between studies (e.g. imaging methods, brain regions). Bustamante et al. [39] focused on children and adolescents with overweight/obesity and interpreted PA-related brain changes of four high-quality studies as beneficial, but their conclusions lack anatomical specificity.

In summary, only a small number of studies have examined PA-related brain changes and while PA-related effects have been reported, findings are inconsistent with little methodological or anatomical overlap between studies.

### Intervention fidelity reporting

Reporting of fidelity is crucial for accurate interpretation of intervention results [45, 46]. This is particularly the case in behavioural interventions where there is substantial heterogeneity in intervention characteristics. Only two (of 19) reviews reported on intervention fidelity in their results section [28, 32] and noted a lack of fidelity reporting in studies. Several other reviews discuss the lack- and importance of reporting fidelity metrics in their discussion [25, 31, 38]. None of the systematic reviews took intervention fidelity into account when summarising the findings of effects of PA on cognitive-, academic-, or brain outcomes.

### Limitations and recommendations

#### Limitations

An overview of limitations included in systematic reviews is provided in Additional file 10. A (non-exhaustive) narrative synthesis is provided here, intended to identify limitations that were common to the discussion section of at least two reviews. One of the most reported limitations is the presence of high heterogeneity across studies: in designs of PA interventions (duration, frequency, resources provided, delivery [32, 40]), the (appropriate) control groups [25, 30, 40], and the measurement tools that were used [29, 30, 41]. Reviews often reported a lack of detailed reporting of interventions (e.g. duration, intensity, compliance, resources, delivery), assessments [30, 32, 38], and potential moderators [24, 34], as well as a lack of valid measurement tools [29, 30]. These within study limitations may contribute to a general lack of high-quality studies [26, 35, 38, 39]. Other limitations that were frequently noted are the lack of studies in adolescents [25, 30, 31] and those covering various sub-domains of cognition/academic achievement [24, 35, 37], the presence of relatively small samples [29, 30] and samples being predominantly from high-income countries [31, 32, 34]. While some reviews tested for the presence of publication bias, this too was often reported as a limitation [23, 25, 40].

#### Recommendations

In addition to resolving the above limitations, authors recommended to include long term follow-up assessments [28, 29, 31, 34, 38], explore the effect of different PA characteristics (intensity, duration) [24, 37], include brain imaging [25, 27, 31], monitor the PA dose that participants receive [25, 37], and explore the influence of effect modifiers (e.g. sex, ethnicity and socioeconomic status [31, 34]). Furthermore, authors suggested to report effect sizes and standardized regression coefficients [25, 41], focus on examining the qualitative aspects of PA [24, 25, 35, 39], explore after-school PA interventions [23, 38] and conduct transitional work, examining what interventions are most effective for implementation in schools [23, 39].

## Discussion

### Key findings

The aim of this review was to critically and systematically evaluate systematic reviews that examined the effects of chronic PA interventions on cognitive-, academic- and brain outcomes in children and adolescents. Of 19 systematic reviews, only one received a high confidence rating and reported inconsistent evidence for PA-related effects on academic performance and favourable effects on executive functions in overweight/obese children, albeit based on results from single studies. Reviews with a (critically) low confidence rating presented mixed favourable or null effects. Reviews that evaluated brain outcomes suggested PA-related brain changes, but with little anatomical or methodological overlap which has complicated the synthesis and interpretation of findings.

In general, the quality of the majority of systematic reviews is considered to be critically low with high heterogeneity between systematic reviews (e.g. number of included studies, presentation of findings). Furthermore, only three systematic reviews took the quality of primary studies into account when synthesising evidence and there is a general lack of reporting of intervention fidelity. Systematic reviews and meta-analyses consistently stated that the field suffers from high heterogeneity between primary studies (e.g. in the design, intervention, outcome measures and reporting) and that high-quality studies are required.

In the following sections we will discuss these key findings and argue that the field would benefit from improvements in study quality, reporting and dissemination.

### Academic-, cognitive- and brain outcomes

The findings on cognitive outcomes are largely in line with conclusions from the UK Expert Working Group Working Paper on children and young people [18]. In contrast, the US 2018 PA Guidelines [12] suggested moderate evidence for PA-related improvements in cognitive and academic performance (among 5–13 year olds), and Biddle et al. [19] claimed a causal association of PA with cognitive functioning and indicated less clear results for academic achievement. The discrepancy in conclusions is likely due to the set of reviews considered for inclusion and the strategy for evidence synthesis. That is, conclusions of both the UK Working Group and US guidelines were based on a small number of systematic reviews (Additional file 11) without mention of review quality or reasons for selecting this (sub) set of reviews. Biddle et al. [19] based their conclusions on a large number of reviews (*n* = 25), including some that combined observational and interventional evidence, without reference to review quality or sub-domains of cognitive- or academic performance.

It is clear that the selection of reviews and strategy for synthesis could impact on conclusions, particularly if the synthesis is based on study authors’ conclusions, which commonly emphasise positive findings (e.g. [24, 33, 38]). Future reports would benefit from a nuanced overview, with conclusions that take into account (objective) findings presented in reviews’ results sections.

### Study quality and methodological considerations

We observed high variability in quality and methods across systematic reviews. In particular, systematic reviews often lacked an a priori design, an overview of excluded studies, an assessment of publication bias (unless a meta-analysis was included), and did not consider quality information in their evidence synthesis. While the majority of reviews described the sample (e.g. size, age, sex) of the included studies, they did not discuss baseline PA levels, which is important for assessing whether the sample is representative of the general population and for the interpretation of reported effects (e.g. greater effects may be expected for a sample of low active individuals [47, 48]). Only a minority of reviews reported on intervention fidelity, which is crucial if one wishes to understand whether observed effects can be ascribed to the intervention or other environmental factors (e.g. PE enjoyment). Furthermore, reviews hardly distinguished between PA interventions (e.g. dose or type) in presenting results. This practice is useful if the goal is to examine whether any form of PA has an effect on cognitive, academic or brain outcomes, yet provides little insight into the appropriate dose or PA type (e.g. enhanced / quantitative or qualitative / enriched PA manipulations) that may benefit outcomes most. Similarly, reviews varied substantially in whether they presented findings by sub-domains or overall cognitive- or academic performance, as well as the definition of sub-domains. If conclusions are to be drawn regarding specific PA effects from findings across reviews, a more granular and consistent presentation of results is required. Finally, reviews included multiple publications stemming from the same study (and sample), which is rarely considered (but see e.g. [31, 39]). For instance, half of the neuroimaging publications included the same sample of overweight children, and care should be taken in generalising these results.

A subset of reviews included meta-analyses, the appropriateness of which has been questioned due to the heterogeneity between studies [27]. Among those that did include a meta-analyses, we observed variability in whether the meta-analysis was pre-registered, in the number of included studies (2–22), and whether one or multiple studies on the same sample were included and appropriately accounted for (e.g. Cochrane handbook 5.1, section 16.5.4 [49]). Importantly, meta-analyses rarely took study quality or study design ([cluster] RCT [50] and non-RCT) into account. This practice could bias effect sizes and requires separate reporting [22]. In addition, not all meta-analyses used inverse variance weighting and only a few meta-analyses fully explored the high observed heterogeneity using meta-regression and / or subgroup analysis [51]. Finally, small sample sizes of primary studies may present with larger effect sizes and thereby affect meta-analyses results [52, 53]. Although meta-analyses are a useful statistical tool to quantitatively synthesise findings [54, 55], care should be taken in their implementation.

### Research gaps

Several gaps and forms of bias were identified in the evidence base. These would need to be addressed before findings about the effects of PA on cognition, academic performance and the brain can be generalised to a wider population and impact on policy.

- **Sample bias** The majority of primary studies and by extension systematic reviews included samples of children (7–12 year old), with far fewer studies on adolescents [30]. While observational evidence in adolescents exists [56, 57], high-quality RCTs are needed to bridge the gap.
- **Geographical bias** The majority of studies were conducted in the USA, and more generally in developed countries. RCTs in less-developed countries, particularly those from Africa and South America (Fig. 2), but also from Asia, are needed to explore whether findings can be generalised to the wider population. The “Cogni-Action” cross-over randomised trial of an acute PA intervention in Chile is one such attempt to close the geographical gap [58].
- **Publication bias** Systematic reviews indicated the presence of publication bias: studies that report on significant, often positive, effects of PA interventions are more likely to be published. For instance, all brain imaging studies included at least one significant outcome. To counteract publication bias, PA researchers should publish non-significant findings and consider preregistering their analysis plans. Moreover, initiatives such as registered reports, journals soliciting negative results, and funder-supported journals that encourage open practices, are warranted.
- **Brain imaging** There was a lack of studies that included brain imaging, in particular T1-weighted (structural) MRI. Consequently, the evidence for PA-related brain changes is based on a small number of studies, 50% of which included the same sample of overweight children. High variability in outcome measures and lack of replication further complicate the interpretation of brain findings. Surprisingly, none of studies that were included in the reviews have examined PA effects on hippocampal metrics (e.g. volume), despite overwhelming evidence from animal studies [59] and various reports in human adults [60]. To uncover neuro-biological mechanisms of exercise, brain imaging (MRI and/or EEG) should be considered as an outcome measure in future intervention studies, e.g. *Cogni-Action* project [58] and *Fit to Study* [61].
- **Moderators** There is little understanding of the influence of potential moderators, such as sex, socio-economic status, ethnicity and genetic background on PA-related cognitive, academic and brain outcomes. Understanding the effect of such moderators could help develop targeted PA interventions for sub-groups of children who may benefit most.

### Recommendations

To address research gaps and improve the quality of both primary studies and systematic reviews, the field is increasingly recognising that current research practice needs to evolve. We therefore provide recommendations, including references to resources, in an attempt to facilitate improvements in evidence generation and synthesis.

Future reviews are encouraged to screen for existing or pre-registered systematic reviews (e.g. on PROSPERO: https://www.crd.york.ac.uk/prospero/ or Open Science Framework, OSF: https://osf.io) on the same topic. At least seven new systematic reviews have been published [62–68] since February 2019, the majority of which reported on the same studies that have been included in previous reviews. Moreover, researchers are encouraged to pre-register their protocol (e.g. on PROSPERO), follow the PRISMA guidelines and consult online resources (e.g. Cochrane handbook [69]), particularly if meta-analyses are conducted [54, 55, 70]. We encourage researchers to think carefully about their research question and inclusion criteria (e.g. type of design, PA interventions and outcomes) which will determine the scope of the review. To further our understanding of PA effects, we recommend researchers to accurately describe the sample of the included studies (including baseline PA levels) and summarise findings by cognitive- or academic sub-domains and PA characteristics, taking into account the quality of the studies, with conclusions providing a balanced summary of findings. For instance, researchers could consider focussing on the high(est) quality studies and, in addition to summarising findings by cognitive sub-domain, show the relationship between dose parameters and outcome measures (e.g. effect sizes, for an example see ref [66]). If a meta-analysis is appropriate, researchers are encouraged to consult guidelines to ensure the correct statistical methods are used (e.g. [54, 55, 70]), heterogeneity and publication bias are assessed, and sub-group and/or sensitivity analyses are conducted (e.g. for cluster-RCT effects [50, 64]).

While a systematic review of reviews allows for an evaluation of the field and provide recommendations for future research and policy [71], it does not allow for a detailed discussion of primary studies. Based on systematic reviews, however, we found that the quality of primary studies is low to moderate. To improve study quality, researchers are advised to consult the protocol- (Standard Protocol Items: Recommendations for Interventional Trials, SPIRIT) [72] and reporting (Consolidated Standards of Reporting Trials, CONSORT) [73, 74] guidelines for trials early on in the design process, and pre-register the study on an independent registry (e.g. ClinicalTrials.gov). An adequate sampling strategy should be followed to ensure an unbiased sample and generalizability of findings [75], and a power analysis ought to be performed a-priori to determine an adequate sample size. PA researchers are encouraged to carefully consider the PA intervention, including the type, the dose, the control group(s) [25], and to implement strategies to measure adherence (e.g. using actigraphy or heart rate monitors). Moreover, the validity of outcome measures should be ensured. Researchers are encouraged to follow the CONSORT guidelines for reporting of RCT outcomes and the Template for intervention description and replication (TIDieR) checklist for intervention reporting [76]. Point estimates, confidence intervals and effect sizes should be provided, and, in addition to an intention-to-treat analysis, instrumental variable or compliance-average causal effect approaches may be used in cases of non-compliance [77, 78]. Missing data should be reported and dealt with appropriately (e.g. using multiple imputation) [79, 80].

### Strengths and limitations

Strengths of this review include its evidence synthesis by review quality and its focus on interventional evidence only, thereby only considering studies from which causality can be inferred. At the same time, relevant systematic reviews may have been excluded. In addition, the AMSTAR-2 criteria merely consider methodological quality, not whether the findings were synthesised appropriately. For instance, Martin et al. [31] based their conclusions for beneficial effects of PA on findings from a single study and Singh et al. [54] used vote-counting to summarise findings, the validity of which has been questioned [54]. None of the reviews took intervention characteristics (dose, type) into account when summarising results of primary studies. Therefore, no conclusions could be drawn regarding the potential differential effects of PA characteristics on outcomes. Reviews on acute PA effects were not considered in this review and may benefit from an independent evaluation [81]. Furthermore, systematic reviews used a variety of quality assessments which prevented a direct comparison of quality of primary studies. We further acknowledge that our summary of limitations and recommendations given by systematic reviews may be influenced by our own interpretations, yet its aim was to provide an overview of common discussion points. Finally, differences in reporting of findings across systematic reviews limited the extent to which evidence at the level of individual cognitive and academic domains was summarised.

## Conclusion

Based on a single high-quality review and 18 (critically) low-quality reviews, we found inconsistent evidence for PA-related effects on cognitive- and academic performance in children and adolescents. Low-quality reviews suggest PA-related brain changes, but lack an interpretation of these findings. If this field were to inform policy, high-quality systematic reviews and primary studies are needed that provide insight into the effect of dose and PA characteristics on (domains of) cognitive-, academic and brain outcomes, in particular in adolescents and children in developing countries.

## Supplementary information

**Additional file 1.** PRISMA 2009 checklist.

**Additional file 2.** Search strategy and search terms.

**Additional file 3.** Excluded studies with reasons.

**Additional file 4.** Search details of systematic reviews.

**Additional file 5.** Countries where PA interventions were conducted.

**Additional file 6.** Quality assessment and bias tools from systematic reviews.

**Additional file 7.** Quality assessment AMSTAR-2.

**Additional file 8.** Overlap of primary studies across reviews.

**Additional file 9.** Supplementary findings from meta-analyses.

**Additional file 10.** Limitations and recommendations of included reviews.

**Additional file 11.** Studies included in PA committee reports.

## Data Availability

All data generated as part of this systematic review are included as additional files in this published article.

## Abbreviations

CONSORT: Consolidated Standards of Reporting Trials
EEG: Electroencephalography
MPA: Moderate physical activity
MRI: Magnetic Resonance Imaging
MVPA: Moderate-to-vigorous physical activity
OSF: Open Science Framework
PA: Physical activity
PAGAC: Physical Activity Guidelines Advisory Committee
PRISMA: Preferred Reporting Items for Systematic Reviews and Meta-analysis
RCT: Randomised controlled trial
SPIRIT: Standard Protocol Items: Recommendations for Interventional Trials
TIDieR: Template for intervention description and replication
VPA: Vigorous physical activity
