# Supplementary material for: A critical evaluation of systematic reviews assessing the effect of chronic physical activity on academic achievement, cognition and the brain in children and adolescents: a systematic review
Source: Int J Behav Nutr Phys Act. 2020 Jun 22;17:79. doi: 10.1186/s12966-020-00959-y (PMC7310146; doi:10.1186/s12966-020-00959-y)
Supplement: Supplementary file 2 — Additional file 2. Search strategy and search terms. [file 12966_2020_959_MOESM2_ESM.docx]

# S2. Search strategy

The literature search was performed on the 21^st^ of June 2018 and updated on the 6^th^ of February 2019 by an information specialist (NR).

## S2.1. Summary of search results

A summary of results of the initial search is provided in Table 1and the updated search in Table 2.

### Table 1. Summary of initial search results (21 June 2018)

| **Database** | **Interface** | **Coverage** | **Date** | **Hits** |
| --- | --- | --- | --- | --- |
| Cochrane Database of Systematic Reviews | Cochrane Library, Wiley | Issue 6 of 12, June 2018 | 21/06/2018 | 33 |
| Database of Abstracts of Reviews of Effects | Cochrane Library, Wiley | Issue 2 of 4 April 2015 | 21/06/2018 | 4 |
| Education Research Information Centre (ERIC) | EBSCOHost |  | 21/06/2018 | 48 |
| Embase | OvidSP | 1974 to present | 21/06/2018 | 286 |
| Medline | OvidSP | 1946-present | 21/06/2018 | 449 |
| PsycINFO | OvidSP | 1806 to present | 21/06/2018 | 74 |
| Science Citation index and Conference Proceedings Citation Index - Science | Web of Science Core Collection, Thomson Reuters | 1945-present | 21/06/2018 | 218 |
| **Total** |  |  |  | **1112** |
| Duplicates |  |  |  | 279 |
| **Final Total** |  |  |  | **833** |

Abbreviations: ERIC = Education Research Information Centre, SCI and CPCI-Science = Science Citation Index and Conference Proceedings Citation Index – Science

### Table 2. Summary of updated search results (6 February 2019)

| **Database** | **Interface** | **Coverage** | **Date** | **Hits** |
| --- | --- | --- | --- | --- |
| Cochrane Database of Systematic Reviews | Cochrane Library, Wiley | Issue 6 of 12, June 2018 | 06/02/2019 | 28 |
| Database of Abstracts of Reviews of Effects | Cochrane Library, Wiley | Issue 2 of 4 April 2015 | 06/02/2019 | 0 |
| Education Research Information Centre (ERIC) | EBSCOHost |  | 06/02/2019 | 0 |
| Embase | OvidSP | 1974 to present | 06/02/2019 | 43 |
| Medline | OvidSP | 1946-present | 06/02/2019 | 63 |
| PsycINFO | OvidSP | 1806 to present | 06/02/2019 | 5 |
| Science Citation index and Conference Proceedings Citation Index - Science | Web of Science Core Collection, Thomson Reuters | 1945-present | 06/02/2019 | 33 |
| **Total** |  |  |  | **172** |
| Duplicates: |  |  |  | 102 |
| **Final Total** |  |  |  | **70** |

Abbreviations: ERIC = Education Research Information Centre, SCI and CPCI-Science = Science Citation Index and Conference Proceedings Citation Index - Science

##

## S2.2. Search terms

Search terms for each of the databases are provided in Tables 3 – 14. Tables 3 – 8 contain the terms and number of hits of the initial search, and Tables 9 – 14 contain the results of the updated search. The updated search was limited to papers that were published in 2018 or 2019.

### Table 3. Medline search– initial search terms

| **ID** | **Searches** | **Results** |
| --- | --- | --- |
| 1 | motor activity/ or exp exercise/ | 249636 |
| 2 | Exercise Test/ and Oxygen Consumption/ | 10408 |
| 3 | physical exertion/ or exp physical fitness/ | 78713 |
| 4 | (physical* adj3 (activ* or fit* or exert* or endurance or train*)).ti,ab. | 113517 |
| 5 | exercis*.ti,ab. | 258475 |
| 6 | (aerobic? or ((resistance or weight or intensity or strength* or endurance or muscleor isometric or circuit*) adj3 (train* or workout? or work out?))).ti,ab. | 93927 |
| 7 | (running or jogging or walk*).ti,ab. | 149899 |
| 8 | ((cardiovascular or cvd) adj2 fitness).ti,ab. | 1425 |
| 9 | ((peak oxygen adj2 (uptake or consumption)) or (peak adj2 vo2)).ti,ab. | 7470 |
| 10 | 1 or 2 or 3 or 4 or 5 or 6 or 7 or 8 or 9 | 654706 |
| 11 | Brain/ and exp Magnetic Resonance Imaging/ | 66851 |
| 12 | exp Neuroimaging/ | 153473 |
| 13 | Cognition/ | 84009 |
| 14 | learning/ | 137822 |
| 15 | exp Educational Measurement/ | 133083 |
| 16 | (brain and (mri or magnetic resonance imaging)).ti. | 6871 |
| 17 | (brain adj5 (mri or magnetic resonance imaging)).ti,ab. | 28515 |
| 18 | (neuroimag* or neuro-imag*).ti,ab. | 41249 |
| 19 | ((white matter or grey matter) and (mri or magentic resonance imaging)).ti,ab. | 14526 |
| 20 | (cognit* adj5 (function* or dysfunction* or impair*)).ti,ab. | 123425 |
| 21 | (brain adj5 (function* or dysfunction* or impair*)).ti,ab. | 72526 |
| 22 | cognition.ti,ab. | 54381 |
| 23 | ((academic or education*) adj5 (achieve* or attain* or performance or success)).ti,ab. | 24165 |
| 24 | 11 or 12 or 13 or 14 or 15 or 16 or 17 or 18 or 19 or 20 or 21 or 22 or 23 | 677919 |
| 25 | adolescent/ or exp child/ | 2788574 |
| 26 | PEDIATRICS/ | 49356 |
| 27 | (child* or schoolchild* or preschool* or pre-school* or girl* or boy* or pediatric* or paediatric*).ti,ab. | 1497750 |
| 28 | 25 or 26 or 27 | 3263804 |
| 29 | 10 and 24 and 28 | 4281 |
| 30 | (MEDLINE or systematic review).tw. or meta analysis.pt. or review.pt. | 2466154 |
| 31 | 29 and 30 | 479 |
| 32 | limit 31 to english language | 449 |

### Table 4. Embase search – initial search terms

| **ID** | **Searches** | **Results** |
| --- | --- | --- |
| 1 | exp *physical activity/ or exp *exercise/ | 232527 |
| 2 | exercise test/ and oxygen consumption/ | 8477 |
| 3 | *fitness/ | 15073 |
| 4 | (physical* adj3 (activ* or fit* or exert* or endurance or train*)).ti,ab. | 153160 |
| 5 | exercis*.ti,ab. | 343650 |
| 6 | (aerobic? or ((resistance or weight or intensity or strength* or endurance or muscleor isometric or circuit*) adj3 (train* or workout? or work out?))).ti,ab. | 113934 |
| 7 | (running or jogging or walk*).ti,ab. | 199157 |
| 8 | ((cardiovascular or cvd) adj2 fitness).ti,ab. | 1843 |
| 9 | ((peak oxygen adj2 (uptake or consumption)) or (peak adj2 vo2)).ti,ab. | 11699 |
| 10 | 1 or 2 or 3 or 4 or 5 or 6 or 7 or 8 or 9 | 750238 |
| 11 | brain/ and exp nuclear magnetic resonance imaging/ | 50217 |
| 12 | [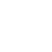](http://ezproxy-prd.bodleian.ox.ac.uk:2081/sp-3.27.2b/ovidweb.cgi?&S=CIKPFPKCFFDDHMDENCFKHGDCNKEGAA00&R=14&Search+Annotations+Options=S)exp *neuroimaging/ | 19795 |
| 13 | *Cognition/ | 67574 |
| 14 | *learning/ | 60579 |
| 15 | exp academic achievement/ or "outcome of education"/ or school admission/ or exp school attendance/ | 34210 |
| 16 | (brain and (mri or magnetic resonance imaging)).ti. | 9544 |
| 17 | (brain adj5 (mri or magnetic resonance imaging)).ti,ab. | 52303 |
| 18 | (neuroimag* or neuro-imag*).ti,ab. | 60519 |
| 19 | ((white matter or grey matter) and (mri or magentic resonance imaging)).ti,ab. | 27544 |
| 20 | (cognit* adj5 (function* or dysfunction* or impair*)).ti,ab. | 185709 |
| 21 | (brain adj5 (function* or dysfunction* or impair*)).ti,ab. | 97485 |
| 22 | [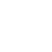](http://ezproxy-prd.bodleian.ox.ac.uk:2081/sp-3.27.2b/ovidweb.cgi?&S=CIKPFPKCFFDDHMDENCFKHGDCNKEGAA00&R=24&Search+Annotations+Options=S)[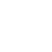](http://ezproxy-prd.bodleian.ox.ac.uk:2081/sp-3.27.2b/ovidweb.cgi?&S=CIKPFPKCFFDDHMDENCFKHGDCNKEGAA00&R=26&Search+Annotations+Options=S)cognition.ti,ab. | 76723 |
| 23 | ((academic or education*) adj5 (achieve* or attain* or performance or success)).ti,ab. | 29327 |
| 24 | 11 or 12 or 13 or 14 or 15 or 16 or 17 or 18 or 19 or 20 or 21 or 22 or 23 | 580616 |
| 25 | child/ or preschool child/ or school child/ or toddler/ | 2E+06 |
| 26 | PEDIATRICS/ | 72610 |
| 27 | (child* or schoolchild* or preschool* or pre-school* or girl* or boy* or pediatric* or paediatric*).ti,ab. | 2E+06 |
| 28 | 25 or 26 or 27 | 3E+06 |
| 29 | 10 and 24 and 28 | 3463 |
| 30 | (meta-analysis or systematic review).tw. or review.pt. | 3E+06 |
| 31 | 29 and 30 | 312 |
| 32 | limit 31 to english language | 286 |

### Table 5. PsychInfo - initial search terms

| **ID** | **Searches** | **Results** |
| --- | --- | --- |
| 1 | exp physical activity/ | 36604 |
| 2 | physical fitness/ | 3979 |
| 3 | exp exercise/ | 23784 |
| 4 | (physical* adj3 (activ* or fit* or exert* or endurance or train*)).ti,ab. | 35422 |
| 5 | exercis*.ti,ab. | 60554 |
| 6 | (aerobic? or ((resistance or weight or intensity or strength* or endurance or muscleor isometric or circuit*) adj3 (train* or workout? or work out?))).ti,ab. | 6775 |
| 7 | (running or jogging or walk*).ti,ab. | 37831 |
| 8 | ((cardiovascular or cvd) adj2 fitness).ti,ab. | 430 |
| 9 | ((peak oxygen adj2 (uptake or consumption)) or (peak adj2 vo2)).ti,ab. | 351 |
| 10 | 1 or 2 or 3 or 4 or 5 or 6 or 7 or 8 or 9 | 126818 |
| 11 | exp Brain/ and exp Magnetic Resonance Imaging/ | 20798 |
| 12 | exp neuroimaging/ | 82124 |
| 13 | Cognition/ | 30474 |
| 14 | exp academic achievement/ or academic aptitude/ or academic failure/ or educational attainment level/ or exp educational measurement/ | 91703 |
| 15 | learning/ | 58972 |
| 16 | exp academic achievement/ or "outcome of education"/ or school admission/ or exp school attendance/ | 73720 |
| 17 | (brain and (mri or magnetic resonance imaging)).ti. | 1523 |
| 18 | (brain adj5 (mri or magnetic resonance imaging)).ti,ab. | 8601 |
| 19 | (neuroimag* or neuro-imag*).ti,ab. | 24597 |
| 20 | ((white matter or grey matter) and (mri or magentic resonance imaging)).ti,ab. | 5398 |
| 21 | (cognit* adj5 (function* or dysfunction* or impair*)).ti,ab. | 86150 |
| 22 | (brain adj5 (function* or dysfunction* or impair*)).ti,ab. | 38371 |
| 23 | cognition.ti,ab. | 68417 |
| 24 | ((academic or education*) adj5 (achieve* or attain* or performance or success)).ti,ab. | 54270 |
| 25 | 11 or 12 or 13 or 15 or 16 or 17 or 18 or 19 or 20 or 21 or 22 or 23 or 24 | 407550 |
| 26 | (adolescence 13 17 yrs or childhood birth 12 yrs).ag. | 734961 |
| 27 | PEDIATRICS/ | 22592 |
| 28 | (child* or schoolchild* or preschool* or pre-school* or girl* or boy* or pediatric* or paediatric*).ti,ab. | 704751 |
| 29 | 26 or 27 or 28 | 1E+06 |
| 30 | 10 and 25 and 29 | 2234 |
| 31 | (meta-analysis or search:).tw. or meta analysis/ or "literature review"/ | 128981 |
| 32 | 30 and 31 | 76 |
| 33 | limit 32 to english language | 74 |

### Table 6. Cochrane Library – initial search terms

| **ID** | **Search** |
| --- | --- |
| #1 | MeSH descriptor: [Motor Activity] explode all trees |
| #2 | MeSH descriptor: [Exercise] explode all trees |
| #3 | MeSH descriptor: [Physical Fitness] explode all trees |
| #4 | MeSH descriptor: [Physical Exertion] explode all trees |
| #5 | (physical* near/3 (activ* or fit* or exert* or endurance or train*)):ti,ab,kw or exercis*:ti,ab,kw or (aerobic* or (resistance or weight or intensity or strength* or endurance or muscleor isometric or circuit*)):ti,ab,kw or running or jogging or walk*:ti,ab,kw (Word variations have been searched) |
| #6 | ((cardiovascular or cvd) near/2 fitness):ti,ab,kw or ((peak oxygen near/2 (uptake or consumption)) or (peak near/2 vo2)):ti,ab,kw (Word variations have been searched) |
| #7 | #1 or #2 or #3 or #4 or #5 or #6 |
| #8 | MeSH descriptor: [Neuroimaging] explode all trees |
| #9 | MeSH descriptor: [Cognition] this term only |
| #10 | MeSH descriptor: [Learning] this term only |
| #11 | MeSH descriptor: [Educational Measurement] explode all trees |
| #12 | (brain and (mri or magnetic resonance imaging)):ti or (brain near (mri or magnetic resonance imaging)):ti,ab,kw or (neuroimag* or neuro-imag*):ti,ab,kw or ((white matter or grey matter) and (mri or magentic resonance imaging)):ti,ab,kw (Word variations have been searched) |
| #13 | (cognit* near (function* or dysfunction* or impair*)):ti,ab,kw or (brain near (function* or dysfunction* or impair*)):ti,ab,kw or cognition:ti,ab,kw or ((academic or education*) near (achieve* or attain* or performance or success)):ti,ab,kw (Word variations have been searched) |
| #14 | #8 or #9 or #10 or #11 or #12 or #13 |
| #15 | MeSH descriptor: [Child] explode all trees |
| #16 | MeSH descriptor: [Adolescent] explode all trees |
| #17 | MeSH descriptor: [Pediatrics] explode all trees |
| #18 | child* or schoolchild* or preschool* or pre-school* or girl* or boy* or pediatric* or paediatric*:ti,ab,kw (Word variations have been searched) |
| #19 | #15 or #16 or #17 or #18 |
| #20 | #7 and #14 and #19 |

### Table 7. Web of Science – initial search terms

| **ID** | **Results** | **Search** |
| --- | --- | --- |
| # 1 | 715,440 | TS=((physical* NEAR3 (activ* or fit* or exert* or endurance or train*))) OR TS=(exercis*) OR TS=((aerobic? or ((resistance or weight or intensity or strength* or endurance or muscleor isometric or circuit*) NEAR3 (train* or workout? or "work out?")))) OR TS=(running or jogging or walk*) OR TS=(((cardiovascular or cvd) NEAR2 fitness)) OR TS=(("peak oxygen" NEAR2 (uptake or consumption)) or (peak NEAR2 vo2)) |
| # 2 | 527,507 | TI=((brain and (mri or "magnetic resonance imaging"))) OR TS=((brain NEAR5 (mri or "magnetic resonance imaging"))) OR TS=(neuroimag* or neuro-imag*) OR TS=((("white matter" or "grey matter") and (mri or "magentic resonance imaging"))) OR TS=((cognit* NEAR5 (function* or dysfunction* or impair*))) OR TS=((brain NEAR5 (function* or dysfunction* or impair*))) or TS=(cognition OR learning) OR TS=((academic or education*) NEAR5 (achieve* or attain* or performance or success)) |
| # 3 | 1,363,929 | TS=(child* or schoolchild* or preschool* or pre-school* or girl* or boy* or pediatric* or paediatric*) |
| # 4 | 1,503 | #3 AND #2 AND #1 |
| # 5 | 314,663 | TS=("systematic review" OR meta-analysis OR metaanalysis) |
| # 6 | 104 | #5 AND #4 |
| # 7 | 155 | (#3 AND #2 AND #1) AND DOCUMENT TYPES: (Review) |
| # 8 | 224 | #7 OR #6 |
| # 9 | 218 | #7 OR #6 Refined by: LANGUAGES: ( ENGLISH ) |

### Table 8. ERIC – initial search terms

| **ID** | **Search** | **Results** |
| --- | --- | --- |
| S1 | TX ( (physical* N3 (activ* or fit* or exert* or endurance or train*)) ) OR TX exercis* OR TX ( (aerobic* or ((resistance or weight or intensity or strength* or endurance or "muscleor isometric" or circuit*) N3 (train* or workout* or "work out*"))) ) OR TX ( running or jogging or walk* ) OR TX ( ((cardiovascular or cvd) N2 fitness) ) OR ( (("peak oxygen" N2 (uptake or consumption)) or (peak N2 vo2)) ) | 56,128 |
| S2 | TI ( (brain and (mri or magnetic resonance imaging)) ) OR TX ( (brain N5 (mri or magnetic resonance imaging)) ) OR TX ( neuroimag* or neuro-imag* ) OR TX ( (("white matter" or "grey matter") and (mri or "magentic resonance imaging")) ) OR TX ( (cognit* N5 (function* or dysfunction* or impair*)) ) OR TX ( (brain N5 (function* or dysfunction* or impair*)) ) OR TX ( cognition or learning ) OR TX ( ((academic or education*) N5 (achieve* or attain* or performance or success)) ) | 541,058 |
| S3 | TX child* or schoolchild* or preschool* or pre-school* or girl* or boy* or pediatric* or paediatric* | 369,508 |
| S4 | S1 AND S2 AND S3 | 4,405 |
| S5 | TX ( MEDLINE or "systematic review" or meta-analysis ) OR TI review | 25,253 |
| S6 | S4 AND S5 | 48 |

### Table 9. Medline – updated search terms

| **ID** | **Searches** | **Results** |
| --- | --- | --- |
| 1 | motor activity/ or exp exercise/ | 259031 |
| 2 | Exercise Test/ and Oxygen Consumption/ | 10722 |
| 3 | physical exertion/ or exp physical fitness/ | 79759 |
| 4 | (physical* adj3 (activ* or fit* or exert* or endurance or train*)).ti,ab. | 120299 |
| 5 | exercis*.ti,ab. | 268415 |
| 6 | (aerobic? or ((resistance or weight or intensity or strength* or endurance or muscleor isometric or circuit*) adj3 (train* or workout? or work out?))).ti,ab. | 98136 |
| 7 | (running or jogging or walk*).ti,ab. | 156801 |
| 8 | ((cardiovascular or cvd) adj2 fitness).ti,ab. | 1487 |
| 9 | ((peak oxygen adj2 (uptake or consumption)) or (peak adj2 vo2)).ti,ab. | 7829 |
| 10 | 1 or 2 or 3 or 4 or 5 or 6 or 7 or 8 or 9 | 680172 |
| 11 | Brain/ and exp Magnetic Resonance Imaging/ | 71699 |
| 12 | exp Neuroimaging/ | 158308 |
| 13 | Cognition/ | 87437 |
| 14 | cognition/ or learning/ | 143137 |
| 15 | exp Educational Measurement/ | 137735 |
| 16 | (brain and (mri or magnetic resonance imaging)).ti. | 7258 |
| 17 | (brain adj5 (mri or magnetic resonance imaging)).ti,ab. | 30187 |
| 18 | (neuroimag* or neuro-imag*).ti,ab. | 43772 |
| 19 | ((white matter or grey matter) and (mri or magentic resonance imaging)).ti,ab. | 15387 |
| 20 | (cognit* adj5 (function* or dysfunction* or impair*)).ti,ab. | 131199 |
| 21 | (brain adj5 (function* or dysfunction* or impair*)).ti,ab. | 76411 |
| 22 | cognition.ti,ab. | 58145 |
| 23 | ((academic or education*) adj5 (achieve* or attain* or performance or success)).ti,ab. | 25616 |
| 24 | 11 or 12 or 13 or 14 or 15 or 16 or 17 or 18 or 19 or 20 or 21 or 22 or 23 | 706452 |
| 25 | adolescent/ or exp child/ | 3E+06 |
| 26 | PEDIATRICS/ | 50598 |
| 27 | (child* or schoolchild* or preschool* or pre-school* or girl* or boy* or pediatric* or paediatric*).ti,ab. | 2E+06 |
| 28 | 25 or 26 or 27 | 3E+06 |
| 29 | 10 and 24 and 28 | 4520 |
| 30 | (MEDLINE or systematic review).tw. or meta analysis.pt. or review.pt. | 3E+06 |
| 31 | 29 and 30 | 504 |
| 32 | limit 31 to english language | 473 |
| 33 | (2018* or 2019*).ed,yr. | 2E+06 |
| 34 | 32 and 33 | 63 |

### Table 10. Embase - updated search terms

| **ID** | **Searches** | **Results** |
| --- | --- | --- |
| 1 | exp *physical activity/ or exp *exercise/ | 234409 |
| 2 | exercise test/ and oxygen consumption/ | 8333 |
| 3 | *fitness/ | 14092 |
| 4 | (physical* adj3 (activ* or fit* or exert* or endurance or train*)).ti,ab. | 161278 |
| 5 | exercis*.ti,ab. | 353349 |
| 6 | (aerobic? or ((resistance or weight or intensity or strength* or endurance or muscleor isometric or circuit*) adj3 (train* or workout? or work out?))).ti,ab. | 118054 |
| 7 | (running or jogging or walk*).ti,ab. | 207967 |
| 8 | ((cardiovascular or cvd) adj2 fitness).ti,ab. | 1924 |
| 9 | ((peak oxygen adj2 (uptake or consumption)) or (peak adj2 vo2)).ti,ab. | 12168 |
| 10 | 1 or 2 or 3 or 4 or 5 or 6 or 7 or 8 or 9 | 770308 |
| 11 | brain/ and exp nuclear magnetic resonance imaging/ | 51388 |
| 12 | exp *neuroimaging/ | 21094 |
| 13 | *Cognition/ | 67954 |
| 14 | *learning/ | 58636 |
| 15 | exp academic achievement/ or "outcome of education"/ or school admission/ or exp school attendance/ | 35591 |
| 16 | (brain and (mri or magnetic resonance imaging)).ti. | 10023 |
| 17 | (brain adj5 (mri or magnetic resonance imaging)).ti,ab. | 55362 |
| 18 | (neuroimag* or neuro-imag*).ti,ab. | 63660 |
| 19 | ((white matter or grey matter) and (mri or magentic resonance imaging)).ti,ab. | 28930 |
| 20 | (cognit* adj5 (function* or dysfunction* or impair*)).ti,ab. | 195578 |
| 21 | (brain adj5 (function* or dysfunction* or impair*)).ti,ab. | 101246 |
| 22 | cognition.ti,ab. | 81538 |
| 23 | ((academic or education*) adj5 (achieve* or attain* or performance or success)).ti,ab. | 30967 |
| 24 | 11 or 12 or 13 or 14 or 15 or 16 or 17 or 18 or 19 or 20 or 21 or 22 or 23 | 601602 |
| 25 | child/ or preschool child/ or school child/ or toddler/ | 2E+06 |
| 26 | PEDIATRICS/ | 70145 |
| 27 | (child* or schoolchild* or preschool* or pre-school* or girl* or boy* or pediatric* or paediatric*).ti,ab. | 2E+06 |
| 28 | 25 or 26 or 27 | 3E+06 |
| 29 | 10 and 24 and 28 | 3701 |
| 30 | (meta-analysis or systematic review).tw. or review.pt. | 3E+06 |
| 31 | 29 and 30 | 334 |
| 32 | limit 31 to english language | 308 |
| 33 | (2018* or 2019*).dc,yr. | 2E+06 |
| 34 | 32 and 33 | 42 |

### Table 11. PsychINFO – updated search terms

| **ID** | **Searches** | **Results** |
| --- | --- | --- |
| 1 | exp physical activity/ | 37810 |
| 2 | physical fitness/ | 4053 |
| 3 | exp exercise/ | 24338 |
| 4 | (physical* adj3 (activ* or fit* or exert* or endurance or train*)).ti,ab. | 36799 |
| 5 | exercis*.ti,ab. | 62170 |
| 6 | (aerobic? or ((resistance or weight or intensity or strength* or endurance or muscleor isometric or circuit*) adj3 (train* or workout? or work out?))).ti,ab. | 7024 |
| 7 | (running or jogging or walk*).ti,ab. | 38794 |
| 8 | ((cardiovascular or cvd) adj2 fitness).ti,ab. | 441 |
| 9 | ((peak oxygen adj2 (uptake or consumption)) or (peak adj2 vo2)).ti,ab. | 365 |
| 10 | 1 or 2 or 3 or 4 or 5 or 6 or 7 or 8 or 9 | 130540 |
| 11 | exp Brain/ and exp Magnetic Resonance Imaging/ | 21512 |
| 12 | exp neuroimaging/ | 84633 |
| 13 | Cognition/ | 31437 |
| 14 | exp academic achievement/ or academic aptitude/ or academic failure/ or educational attainment level/ or exp educational measurement/ | 93214 |
| 15 | learning/ | 60729 |
| 16 | exp academic achievement/ or "outcome of education"/ or school admission/ or exp school attendance/ | 74995 |
| 17 | (brain and (mri or magnetic resonance imaging)).ti. | 1574 |
| 18 | (brain adj5 (mri or magnetic resonance imaging)).ti,ab. | 8880 |
| 19 | (neuroimag* or neuro-imag*).ti,ab. | 25531 |
| 20 | ((white matter or grey matter) and (mri or magentic resonance imaging)).ti,ab. | 5604 |
| 21 | (cognit* adj5 (function* or dysfunction* or impair*)).ti,ab. | 89303 |
| 22 | (brain adj5 (function* or dysfunction* or impair*)).ti,ab. | 39751 |
| 23 | cognition.ti,ab. | 70869 |
| 24 | ((academic or education*) adj5 (achieve* or attain* or performance or success)).ti,ab. | 55973 |
| 25 | 11 or 12 or 13 or 15 or 16 or 17 or 18 or 19 or 20 or 21 or 22 or 23 or 24 | 419641 |
| 26 | (adolescence 13 17 yrs or childhood birth 12 yrs).ag. | 747912 |
| 27 | PEDIATRICS/ | 23202 |
| 28 | (child* or schoolchild* or preschool* or pre-school* or girl* or boy* or pediatric* or paediatric*).ti,ab. | 719174 |
| 29 | 26 or 27 or 28 | 1043558 |
| 30 | 10 and 25 and 29 | 2307 |
| 31 | (meta-analysis or search:).tw. or meta analysis/ or "literature review"/ | 133038 |
| 32 | 30 and 31 | 78 |
| 33 | limit 32 to english language | 74 |
| 34 | (2018* or 2019*).up,yr. | 187094 |
| 35 | 33 and 34 | 5 |

### Table 12. Cochrane Library – updated search terms

| **ID** | **Search** |
| --- | --- |
| #1 | MeSH descriptor: [Motor Activity] explode all trees |
| #2 | MeSH descriptor: [Exercise] explode all trees |
| #3 | MeSH descriptor: [Physical Fitness] explode all trees |
| #4 | MeSH descriptor: [Physical Exertion] explode all trees |
| #5 | (physical* near/3 (activ* or fit* or exert* or endurance or train*)):ti,ab,kw or exercis*:ti,ab,kw or (aerobic* or (resistance or weight or intensity or strength* or endurance or muscleor isometric or circuit*)):ti,ab,kw or running or jogging or walk*:ti,ab,kw (Word variations have been searched) |
| #6 | ((cardiovascular or cvd) near/2 fitness):ti,ab,kw or ((peak oxygen near/2 (uptake or consumption)) or (peak near/2 vo2)):ti,ab,kw (Word variations have been searched) |
| #7 | #1 or #2 or #3 or #4 or #5 or #6 |
| #8 | MeSH descriptor: [Neuroimaging] explode all trees |
| #9 | MeSH descriptor: [Cognition] this term only |
| #10 | MeSH descriptor: [Learning] this term only |
| #11 | MeSH descriptor: [Educational Measurement] explode all trees |
| #12 | (brain and (mri or magnetic resonance imaging)):ti or (brain near (mri or magnetic resonance imaging)):ti,ab,kw or (neuroimag* or neuro-imag*):ti,ab,kw or ((white matter or grey matter) and (mri or magentic resonance imaging)):ti,ab,kw (Word variations have been searched) |
| #13 | (cognit* near (function* or dysfunction* or impair*)):ti,ab,kw or (brain near (function* or dysfunction* or impair*)):ti,ab,kw or cognition:ti,ab,kw or ((academic or education*) near (achieve* or attain* or performance or success)):ti,ab,kw (Word variations have been searched) |
| #14 | #8 or #9 or #10 or #11 or #12 or #13 |
| #15 | MeSH descriptor: [Child] explode all trees |
| #16 | MeSH descriptor: [Adolescent] explode all trees |
| #17 | MeSH descriptor: [Pediatrics] explode all trees |
| #18 | child* or schoolchild* or preschool* or pre-school* or girl* or boy* or pediatric* or paediatric*:ti,ab,kw (Word variations have been searched) |
| #19 | #15 or #16 or #17 or #18 |
| #20 | #7 and #14 and #19 |

### Table 13. Web of Science – update search terms

| **ID** | **Results** | **Search** |
| --- | --- | --- |
| # 1 | 905,908 | TS=((physical* NEAR3 (activ* or fit* or exert* or endurance or train*))) OR TS=(exercis*) OR TS=((aerobic? or ((resistance or weight or intensity or strength* or endurance or muscleor isometric or circuit*) NEAR3 (train* or workout? or "work out?")))) OR TS=(running or jogging or walk*) OR TS=(((cardiovascular or cvd) NEAR2 fitness)) OR TS=(("peak oxygen" NEAR2 (uptake or consumption)) or (peak NEAR2 vo2)) |
| # 2 | 807,679 | TI=((brain and (mri or "magnetic resonance imaging"))) OR TS=((brain NEAR5 (mri or "magnetic resonance imaging"))) OR TS=(neuroimag* or neuro-imag*) OR TS=((("white matter" or "grey matter") and (mri or "magentic resonance imaging"))) OR TS=((cognit* NEAR5 (function* or dysfunction* or impair*))) OR TS=((brain NEAR5 (function* or dysfunction* or impair*))) or TS=(cognition OR learning) OR TS=((academic or education*) NEAR5 (achieve* or attain* or performance or success)) |
| # 3 | 1,451,284 | TS=(child* or schoolchild* or preschool* or pre-school* or girl* or boy* or pediatric* or paediatric*) |
| # 4 | 1,910 | #3 AND #2 AND #1 |
| # 5 | 347,620 | TS=("systematic review" OR meta-analysis OR metaanalysis) |
| # 6 | 113 | #5 AND #4 |
| # 7 | 169 | (#3 AND #2 AND #1) AND DOCUMENT TYPES: (Review) |
| # 8 | 244 | #7 OR #6 |
| # 9 | 33 | (#7 OR #6) AND LANGUAGE: (English) |

### Table 14. ERIC – updated search terms

| **ID** | **Search** | **Results** |
| --- | --- | --- |
| S1 | TX ( (physical* N3 (activ* or fit* or exert* or endurance or train*)) ) OR TX exercis* OR TX ( (aerobic* or ((resistance or weight or intensity or strength* or endurance or "muscleor isometric" or circuit*) N3 (train* or workout* or "work out*"))) ) OR TX ( running or jogging or walk* ) OR TX ( ((cardiovascular or cvd) N2 fitness) ) OR ( (("peak oxygen" N2 (uptake or consumption)) or (peak N2 vo2)) ) | 56,128 |
| S2 | TI ( (brain and (mri or magnetic resonance imaging)) ) OR TX ( (brain N5 (mri or magnetic resonance imaging)) ) OR TX ( neuroimag* or neuro-imag* ) OR TX ( (("white matter" or "grey matter") and (mri or "magentic resonance imaging")) ) OR TX ( (cognit* N5 (function* or dysfunction* or impair*)) ) OR TX ( (brain N5 (function* or dysfunction* or impair*)) ) OR TX ( cognition or learning ) OR TX ( ((academic or education*) N5 (achieve* or attain* or performance or success)) ) | 541,058 |
| S3 | TX child* or schoolchild* or preschool* or pre-school* or girl* or boy* or pediatric* or paediatric* | 369,508 |
| S4 | S1 AND S2 AND S3 | 4,405 |
| S5 | TX ( MEDLINE or "systematic review" or meta-analysis ) OR TI review | 25,253 |
| S6 | S4 AND S5 | 48 |
| S7 | S4 AND S5   Limiters - Date Published: 20180101-20191231 | 0 |
