# Supplementary material for: A critical evaluation of systematic reviews assessing the effect of chronic physical activity on academic achievement, cognition and the brain in children and adolescents: a systematic review
Source: Int J Behav Nutr Phys Act. 2020 Jun 22;17:79. doi: 10.1186/s12966-020-00959-y (PMC7310146; doi:10.1186/s12966-020-00959-y)
Supplement: Supplementary file 3 — Additional file 3. Excluded studies with reasons. [file 12966_2020_959_MOESM3_ESM.docx]

# S3. Excluded studies

A list of excluded studies is provided in Table 1. The main reasons for exclusion were:

- **Different target population**: includes reviews that also considered adults or pre-school aged children.
- **Mixture of acute and chronic**: includes reviews that did not distinguish between acute and chronic PA interventions in their results section or synthesis. The summary of results is a mixture of findings from both types of interventions.
- **Not systematic review**: includes reviews that cannot be considered systematic reviews, either because they did not perform a systematic search of the literature or because they missed crucial elements of a systematic review, such as clear inclusion / exclusion criteria.
- **Different (in)dependent measure**: includes reviews that did not consider cognitive, academic, or brain-related outcomes, or that did not consider solely PA interventions (e.g. sensori-motor or diet interventions).
- **Mixture of study designs**: includes reviews that also included observational study designs.
- **No intervention designs**: includes reviews where no intervention designs were included

Two out of five studies that were identified through other sources and manually added to the list of selected studies were later removed due to them not meeting the inclusion criteria. In particular, one contained a mixture of observational and interventional studies and the other study contained a mixture of acute and chronic physical activity interventions.

### Table 1. Excluded studies with reasons

| **PubMed ID** | **Title** | **Journal** | **Author** | **Reasons** |
| --- | --- | --- | --- | --- |
| 27400928 | Sweat it out? The effects of physical exercise on cognition and behavior in children and adults with ADHD: a systematic literature review | Journal of Neural Transmission | Den Heijer, Anne E. and Groen, Yvonne and Tucha, Lara and Fuermaier, Anselm B. M. and Koerts, Janneke and Lange, Klaus W. and Thome, Johannes and Tucha, Oliver | Mixture of acute and chronic |
| 25387418 | VII. The history of physical activity and academic performance research: informing the future | Monographs of the Society for Research in Child Development | Castelli, Darla M. and Centeio, Erin E. and Hwang, Jungyun and Barcelona, Jeanne M. and Glowacki, Elizabeth M. and Calvert, Hannah G. and Nicksic, Hildi M. | Mixture of acute and chronic |
| 21992090 | Promoting physical activity amongst adolescent girls | Issues in Comprehensive Pediatric Nursing | Standiford Brown, Anne | Not systematic review |
| 24737131 | The WHO Health Promoting School framework for improving the health and well-being of students and their academic achievement | Cochrane Database of Systematic Reviews | Langford, Rebecca and Bonell, Christopher P. and Jones, Hayley E. and Pouliou, Theodora and Murphy, Simon M. and Waters, Elizabeth and Komro, Kelli A. and Gibbs, Lisa F. and Magnus, Daniel and Campbell, Rona | Different (in)dependent variable |
| 26088038 | The Relationship of Health Behaviors to Childhood Cognition and Brain Health | Annals of Nutrition & Metabolism | Hillman, Charles H. and Khan, Naiman A. and Kao, Shih-Chun | Not systematic review |
| 24722921 | The relation of childhood physical activity and aerobic fitness to brain function and cognition: a review | Pediatric Exercise Science | Khan, Naiman A. and Hillman, Charles H. | Not systematic review |
| 29032365 | How does academic achievement relate to cardiorespiratory fitness, self-reported physical activity and objectively reported physical activity: a systematic review in children and adolescents aged 6-18 years | British Journal of Sports Medicine | Marques, Adilson and Santos, Diana A. and Hillman, Charles H. and Sardinha, Luis B. | No interventional designs |
| WOS: 00008982 0600002 | Effects of motor skill intervention on developmental coordination disorder: A meta-analysis | Adapted Physical Activity Quarterly | Pless, M. and Carlsson, M. | Different (in)dependent variable |
| 602074041 | Physically active lessons as physical activity and educational interventions: A systematic review of methods and results | Preventive Medicine | Norris, E. and Shelton, N. and Dunsmuir, S. and Duke-Williams, O. and Stamatakis, E. | Mixture of acute and chronic |
| 24621460 | Possible Cognitive Benefits of Acute Physical Exercise in Children With ADHD | Journal of Attention Disorders | Grassmann, Viviane and Alves, Marcus Vinicius and Santos-Galduroz, Ruth Ferreira and Galduroz, Jose Carlos Fernandes | Different (in)dependent variable |
| 28382668 | The Association of Physical Activity and Academic Behavior: A Systematic Review | Journal of School Health | Sullivan, Rachel A. and Kuzel, AnnMarie H. and Vaandering, Michael E. and Chen, Weiyun | Mixture of acute and chronic |
| 28692579 | Physical Activity Interventions for Children with Social, Emotional, and Behavioral Disabilities-A Systematic Review | Journal of Developmental & Behavioral Pediatrics | Ash, Tayla and Bowling, April and Davison, Kirsten and Garcia, Jeanette | Mixture of acute and chronic |
| 27306434 | Combinations of physical activity, sedentary behaviour and sleep: relationships with health indicators in school-aged children and youth | Applied Physiology, Nutrition, & Metabolism = Physiologie Appliquee, Nutrition et Metabolisme | Saunders, Travis John and Gray, Casey Ellen and Poitras, Veronica Joan and Chaput, Jean-Philippe and Janssen, Ian and Katzmarzyk, Peter T. and Olds, Timothy and Connor Gorber, Sarah and Kho, Michelle E. and Sampson, Margaret and Tremblay, Mark S. and Carson, Valerie | Different (in)dependent variable |
| 25964449 | Exercise Interventions in Children and Adolescents With ADHD: A Systematic Review | Journal of Attention Disorders | Neudecker, Christina and Mewes, Nadine and Reimers, Anne K. and Woll, Alexander | Different (in)dependent variable |
| 27615274 | A Review of Childhood Physical Activity, Brain, and Cognition: Perspectives on the Future | Pediatric Exercise Science | Hillman, Charles H. and Biggan, John R. | Not systematic review |
| 27714852 | Physical fitness and academic performance in youth: A systematic review | Scandinavian Journal of Medicine & Science in Sports | Santana, C. C. A. and Azevedo, L. B. and Cattuzzo, M. T. and Hill, J. O. and Andrade, L. P. and Prado, W. L. | No interventional designs |
| 21807669 | Physical activity and mental health in children and adolescents: a review of reviews | British Journal of Sports Medicine | Biddle, Stuart J. H. and Asare, Mavis | Not systematic review |
| 21281664 | The effects of physical activity on attention deficit hyperactivity disorder symptoms: the evidence | Preventive Medicine | Gapin, Jennifer I. and Labban, Jeffrey D. and Etnier, Jennifer L. | Not systematic review |
| 29387718 | Effects of Physical Activity on Motor Skills and Cognitive Development in Early Childhood: A Systematic Review | BioMed Research International | Zeng, Nan and Ayyub, Mohammad and Sun, Haichun and Wen, Xu and Xiang, Ping and Gao, Zan | Different target population |
| 29671803 | Physical Activity and Cognitive Functioning of Children: A Systematic Review | International Journal of Environmental Research & Public Health [Electronic Resource] | Bidzan-Bluma, Ilona and Lipowska, Malgorzata | Mixture of observational and interventional |
| 18094706 | Be smart, exercise your heart: exercise effects on brain and cognition | Nature Reviews Neuroscience | Hillman, Charles H. and Erickson, Kirk I. and Kramer, Arthur F. | Not systematic review |
| 7728720 | The effects of regular moderate to vigorous physical activity on student outcomes: a review | Canadian Journal of Public Health | Keays, J. J. and Allison, K. R. | Not systematic review |
| 21281669 | A review of chronic and acute physical activity participation on neuroelectric measures of brain health and cognition during childhood | Preventive Medicine | Hillman, Charles H. and Kamijo, Keita and Scudder, Mark | Not systematic review |
| 27419040 | The relationship between physical activity and diet and young children’s cognitive development: A systematic review | Preventive Medicine Reports | Tandon, Pooja S. and Tovar, Alison and Jayasuriya, Avanthi T. and Welker, Emily and Schober, Daniel J. and Copeland, Kristen and Dev, Dipti A. and Murriel, Ashleigh L. and Amso, Dima and Ward, Dianne S. | Mixture of observational and interventional |
| 28847618 | Systematic review of the relationship between 20m shuttle run performance and health indicators among children and youth | Journal of Science & Medicine in Sport | Lang, Justin J. and Belanger, Kevin and Poitras, Veronica and Janssen, Ian and Tomkinson, Grant R. and Tremblay, Mark S. | No interventional designs |
| 25988743 | The effects of physical exercise in children with attention deficit hyperactivity disorder: a systematic review and meta-analysis of randomized control trials | Child: Care, Health & Development | Cerrillo-Urbina, A. J. and Garcia-Hermoso, A. and Sanchez-Lopez, M. and Pardo-Guijarro, M. J. and Santos Gomez, J. L. and Martinez-Vizcaino, V. | Mixture of acute and chronic |
| 27412579 | A Meta-Analytic Review of the Efficacy of Physical Exercise Interventions on Cognition in Individuals with Autism Spectrum Disorder and ADHD | Journal of Autism & Developmental Disorders | Tan, Beron W. Z. and Pooley, Julie A. and Speelman, Craig P. | Different target population |
| 24788950 | The health benefits of muscular fitness for children and adolescents: a systematic review and meta-analysis | Sports Medicine | Smith, Jordan J. and Eather, Narelle and Morgan, Philip J. and Plotnikoff, Ronald C. and Faigenbaum, Avery D. and Lubans, David R. | No interventional designs |
| 2014-47873-001 | Physical education, school physical activity, school sports and academic performance | The International Journal of Behavioral Nutrition and Physical Activity Vol 5 2008, ArtID 10 | Trudeau, Francois and Shephard, Roy J. | Not systematic review |
| 26823546 | A systematic review of the behavioural outcomes following exercise interventions for children and youth with autism spectrum disorder | Autism | Bremer, Emily and Crozier, Michael and Lloyd, Meghann | Mixture of acute and chronic |
| 27306431 | Systematic review of the relationships between objectively measured physical activity and health indicators in school-aged children and youth | Applied Physiology, Nutrition, & Metabolism = Physiologie Appliquee, Nutrition et Metabolisme | Poitras, Veronica Joan and Gray, Casey Ellen and Borghese, Michael M. and Carson, Valerie and Chaput, Jean-Philippe and Janssen, Ian and Katzmarzyk, Peter T. and Pate, Russell R. and Connor Gorber, Sarah and Kho, Michelle E. and Sampson, Margaret and Tremblay, Mark S. | Mixture of observational and interventional |
| 27182986 | Physical Activity, Fitness, Cognitive Function, and Academic Achievement in Children: A Systematic Review | Medicine & Science in Sports & Exercise | Donnelly, Joseph E. and Hillman, Charles H. and Castelli, Darla and Etnier, Jennifer L. and Lee, Sarah and Tomporowski, Phillip and Lambourne, Kate and Szabo-Reed, Amanda N. | Mixture of observational and interventional |
| 27226208 | Enhanced Physical Activity Improves Selected Outcomes in Children With ADHD: Systematic Review | Western Journal of Nursing Research | Song, MinKyoung and Lauseng, Deborah and Lee, Soohee and Nordstrom, Megan and Katch, Victor | Mixture of acute and chronic |
| 21291905 | The association between school-based physical activity, including physical education, and academic performance: a systematic review of the literature | Preventive Medicine | Rasberry, Catherine N. and Lee, Sarah M. and Robin, Leah and Laris, B. A. and Russell, Lisa A. and Coyle, Karin K. and Nihiser, Allison J. | Mixture of observational and interventional |
| 26197943 | Systematic review of physical activity and cognitive development in early childhood | Journal of Science & Medicine in Sport | Carson, Valerie and Hunter, Stephen and Kuzik, Nicholas and Wiebe, Sandra A. and Spence, John C. and Friedman, Alinda and Tremblay, Mark S. and Slater, Linda and Hinkley, Trina | Different target population |
| 28841890 | Effect of classroom-based physical activity interventions on academic and physical activity outcomes: a systematic review and meta-analysis | International Journal of Behavioral Nutrition & Physical Activity | Watson, Amanda and Timperio, Anna and Brown, Helen and Best, Keren and Hesketh, Kylie D. | Mixture of acute and chronic |
| 2015-39696-020 | Systematic review of sedentary behavior and cognitive development in early childhood | Preventive Medicine: An International Journal Devoted to Practice and Theory | Carson, Valerie and Kuzik, Nicholas and Hunter, Stephen and Wiebe, Sandra A. and Spence, John C. and Friedman, Alinda and Tremblay, Mark S. and Slater, Linda G. and Hinkley, Trina | Different (in)dependent variable |
| 22213750 | Physical activity and performance at school: a systematic review of the literature including a methodological quality assessment | Archives of Pediatrics & Adolescent Medicine | Singh, Amika and Uijtdewilligen, Leonie and Twisk, Jos W. R. and van Mechelen, Willem and Chinapaw, Mai J. M. | Mixture of observational and interventional |
| 21957711 | The effects of physical activity and physical fitness on children’s achievement and cognitive outcomes: a meta-analysis | Research Quarterly for Exercise & Sport | Fedewa, Alicia L. and Ahn, Soyeon | Mixture of acute and chronic |
| 2016-26951-001 | Physical activity and school engagement in youth: A systematic review and meta-analysis | Educational Psychologist | Owen, Katherine B. and Parker, Philip D. and Van Zanden, Brooke and MacMillan, Freya and Astell-Burt, Thomas and Lonsdale, Chris | Different (in)dependent variable |
| 28666470 | The effects of chronic and acute physical activity on working memory performance in healthy participants: a systematic review with meta-analysis of randomized controlled trials | Systematic Reviews | Rathore, Azeem and Lom, Barbara | Different target population |
| 24627300 | Lifestyle intervention for improving school achievement in overweight or obese children and adolescents | Cochrane Database of Systematic Reviews | Martin, Anne and Saunders, David H. and Shenkin, Susan D. and Sproule, John | Different (in)dependent variable |
| 2010-01168-006 | The impact of physical activity and fitness on academic achievement and cognitive performance in children | International Review of Sport and Exercise Psychology | Keeley, Thomas J. and Fox, Kenneth R. | Mixture of observational and interventional |
| CD005495 | Early developmental intervention programmes provided post hospital discharge to prevent motor and cognitive impairment in preterm infants | Cochrane Database of Systematic Reviews | Spittle, Alicia and Orton, Jane and Anderson, Peter J and Boyd, Roslyn and Doyle, Lex W | Different target population |
| 36994372 | The relationship between physical activity and cognition in children: A meta-analysis | Pediatric Exercise Science | Sibley, B. A. and Etnier, J. L. | Mixture of observational and interventional |
| 25108657 | Physical activity and cognition in adolescents: A systematic review | Journal of Science & Medicine in Sport | Esteban-Cornejo, Irene and Tejero-Gonzalez, Carlos Ma and Sallis, James F. and Veiga, Oscar L. | Mixture of observational and interventional |
| 72115729 | Effect of physical activity interventions on children and adolescents with autism spectrum disorder: A systematic review and meta-analysis | Physiotherapy (United Kingdom) | Yang, Y. J. and Siao, M. R. and Tsai, F. T. and Luo, H. J. | Not systematic review |
| 30060590 | Relationships Between Motor Proficiency and Academic Performance in Mathematics and Reading in School-Aged Children and Adolescents: A Systematic Review | International Journal of Environmental Research & Public Health [Electronic Resource] | Macdonald, Kirstin and Milne, Nikki and Orr, Robin and Pope, Rodney | Different (in)dependent variable |
| 28554851 | Executive function in childhood obesity: Promising intervention strategies to optimize treatment outcomes | Appetite | Hayes, Jacqueline F. and Eichen, Dawn M. and Barch, Deanna M. and Wilfley, Denise E. | Not systematic review |
| 30264226 | Effect of physical exercises on attention, motor skill and physical fitness in children with attention deficit hyperactivity disorder: a systematic review | Attention Deficit and Hyperactivity Disorders | S, Jeyanthi and Arumugam, Narkeesh and Parasher, Raju K. | Mixture of acute and chronic |
| Added manually | Association between physical education, school-based physical activity, and academic performance: a systematic review | Retos: nuevas tendencias en educación física, deporte y recreación | Marques, Adilson and Corrales, Francisco Rafael Gómez and Martins, Joào and Catunda, Ricardo and Sarmento Hugo | Mixture of observational and interventional |
| Added manually | Influence of physical fitness on cognitive and academic performance in adolescents: A systematic review from 20052015 | International Review of Sport and Exercise Psychology | Ruiz-Ariza, Alberto and Grao-Cruces, Alberto and Marques de Loureiro, Nuno Eduardo and Martínez-López, Emilio J. | Mixture of acute and chronic |
