# Supplementary material for: A critical evaluation of systematic reviews assessing the effect of chronic physical activity on academic achievement, cognition and the brain in children and adolescents: a systematic review
Source: Int J Behav Nutr Phys Act. 2020 Jun 22;17:79. doi: 10.1186/s12966-020-00959-y (PMC7310146; doi:10.1186/s12966-020-00959-y)
Supplement: Supplementary file 4 — Additional file 4. Search details of systematic reviews. [file 12966_2020_959_MOESM4_ESM.docx]

# S4. Search details of systematic reviews

### Table 1. Search details of systematic reviews

| **Review** | **In-/exclusion criteria** | **Search terms** | **Sources** | **Search date** |
| --- | --- | --- | --- | --- |
| Álvarez-Bueno et al (2017)(1) | 1. Population: Healthy children and adolescents, 2. Intervention: PA interventions, 3. Outcome: academic achievement (on-task behavior, marks, specific tests), 4. Design: RCT, Q-exp, controlled pre-post., 5. Language: English or Spanish only, 6. Search: from inception-October 16, 2016, 7. Excluded: acute PA or adult populations | (1) "physical activity, " "physical education," "exercise," "fitness, " and "sport"; (2) "cognition, " "executive, " "executive function, " "cognitive control, " "intelligence, " "memory, " "attention," and "metacognition"; (3) "academic, " "academic achievement, " "academic grades, " "academic behavior, " "academic performance, " and "classroom behavior"; (4) "children, " "childhood, " "preschooler, " "schoolchildren, " "preadolescent, " "adolescent, " and "adolescence"; and (5) "trial" and "effect$*$" | Medline, Embase, Cochrane Central Register of Controlled Trials, Cochrane Database of Systematic Reviews, Web of Science, and PsycINFO databases | From their inception to October 16, 2016 |
| Álvarez-Bueno et al (2017)(2) | 1. Population: Healthy children (4-18 years), 2. Intervention: PA programs, 3. Outcomes: non-executive and executive functions, metacognition, 4. Design: RCT, Q-Exp, controlled pre-post, 5. Language: English or Spanish only, 6. Search: from inception to October 16, 2016, 7. Excluded if: acute PA or adult populations | "physical activity," "physical education," "exercise," "fitness," and "sport"; "cognition," "executive," "executive function," "cognitive control," "intelligence," "memory," "attention," and "metacognition"; "life skills," "goal setting," "problem solving," and "self-regulation"; "brain development," "brain health," "neural," "neuroelectric," "neurotrophic," "neurotrophin," and "hormone"; "children," "childhood," "pre-schooler," "schoolchildren," "preadolescent," "adolescent," and "adolescence" and "trial" and "effect$*$" | MEDLINE, EMBASE, Cochrane Central Register of Controlled Trials, Cochrane Database of Systematic Reviews, Web of Science, and PsycINFO | From their inception to October 16, 2016 |
| Bustamante, Willi, and Davis (2016)(3) | 1. Population: Overweight or obese children, 2. Intervention: PA, exercise or sport, 3. Outcomes: neurologic, cognitive- or academic performance, 4. Design: intervention studies (acute, quasi-experimental, or RCT), 5. Language: English, 6. Search: until December 2015 | children with either exercise or physical activity, with either overweight, obese, weight status, body mass index, and either cognition, brain function, neurologic function, cognitive function, executive function, or academics | PubMed, Journals@OVID, and Web of Science | Before December 2015 |
| De Greeff et al (2018)(4) | 1. Population: Primary school children (6- 12 years), 2. Intervention: PA interventions, 3. Outcomes: executive functions, attention, academic achievement, 4. Design: RCT, Q-exp, 5. Language: English, 6. Search: from 2000- April 2017, 7. Excluded: special populations (e.g. mental or cognitive disorders), studies without appropriate control conditions, studies of which the intervention consisted of more than just physical activity (e.g. including cognitive tasks) | physical activity, physical fitness, executive functions, cognition, academic performance and children (MESH terms have been provided in the supplementary materials of the paper) | PubMed, Web of Science, MEDLINE and ERIC | Between 2000 and April 2017 |
| Gunnell et al. (2018)(5) | 1. Population: Healthy children (1- 17.99 years), including overweight / obesity, 2. Intervention: exclusively PA and not multiple health behaviors, unless the co-behavior was also applied to the control group. Comparator: volume, duration, frequency, intensity, pattern of PA, 3. Outcomes: Cognitive function, brain function, and brain structure. Intelligence and achievement tests were only included if they came from standardized measures, 4. Design: RCT, 5. Language: English or French, 6. Search: until June 2016, and updated in July 2017, 7. Excluded: exergaming studies, results presented by subgroup, quasi-or non-experimental studies, grey literature | Search terms are provided in the supplementary materials of the paper | MEDLINE (1946-present), EMBASE (1980 to 2015 week 25; 1980- 2017 week 31), Cochrane Central Register of Control Trials (February 2016 and June 2017), and PsycINFO (1806 to April week 1 2016; 1806 to July week 4 2017) EBSCOhost was used to search SPORTDiscus | The original searches were conducted in June 2016 and were updated in July 2017 |
| Haapala (2012)(6) | 1. Population: Children without pathological states, 2. Intervention: physical training studies, 3. Outcomes: standardized test scores or academic skills (reported at baseline and post intervention), 4. Design: randomized intervention with parallel intervention and control, 5. Language: English only, 6. Search: 1966- 2011, updated in March 2012, 7. Excluded: | children, adolescent, young, exercise, physical activity, physical training, sport, physical education, academic performance, cognitive ability, cognition, memory, school performance, concentration, attention | Cochrane Register of Controlled Trials, Medline, Eric, CINAHL, PsychINFO, and ISI Web of Knowledge | 1966- 2011, updated in 2012 |
| Jackson et al (2016)(7) | 1. Population: Children (712 years), 2. Intervention: structured PA intervention for at least 1 month, 3. Outcome: executive functions, 4. Design: RCT, 5. Language:,- 6. Search: inception-February 2016, 7. Excluded: prospective cohort and all retrospective studies, EEG/fMRI was excluded from the analysis | "(exercise OR phys$*$) AND (cognit$*$ OR executive) AND (child$*$ OR pre- adolesc$*$)" | Cochrane Library, EBSCO CINAHL, Ovid MEDLINE, PSYCInfo, Pubmed, and Web of Science | All years (February 1, 2016 and February 29, 2016) |
| Lees and Hopkins (2013)(8) | 1. Children (<19 years), 2. Intervention: aerobic physical activity, 3. Outcomes: mental health, behavior, discipline or cognition, 4. Design: RCT only, 5. Language: English only, 6. Search: April 2013, 7. Excluded: if studies measures only fitness or the success of health promotion or if the study measures only health outcomes specific to a disease state | "Two elements were used in the search strategy, the first being for APA (eg, physical activity, aerobic exercise, or cardiovascular health) and the second being measures of cognitive and psychological outcomes (eg, health, mental health, cognition, achievement, intelligence tests, intelligence) The specific search terms used varied slightly in each database so as to make use of MeSH terms or subheadings More specific keywords such as "aerobic" were at times subsumed by these headings and thus not included, and search terms were kept purposefully broad to ensure that relevant results were not excluded" | MEDLINE, Cochrane, PsycINFO, SPORTDiscus, and EMBASE | April 2013 |
| Li et al (2017)(9) | 1. Population: healthy adolescents (13-18 years, or within 1-year range), 2. Intervention: chronic or acute exercise intervention, 3. Outcome: At least one cognitive or academic performance measure, 4. Design: intervention studies, 5. Language: English only, 6. Search: until 31^st^ October 2016, 7. Excluded: Adolescent populations with learning disorders, reviews, abstracts or theses | The respective keywords for each of these categories were (1) "exercise"; "physical fitness"; "physical activit$*$"; "body training," "physical training"; "sport$*$"; (2) "cognition"; "cognitive function$*$"; "executive function$*$"; "learning"; "mental process$*$"; "attention"; "academic performance"; "academic achievement"; (3) "adolescen$*$"; "child$*$"; "boy$*$"; "girl$*$" | AMED, AusportMed, CINAHL, COCHRANE, Embase, Medline, Scopus, SPORTdiscus, Web of Science | until 31^st^ October 2016 |
| Lubans et al (2016)(10) | 1. Population: school aged (5-18 years) at baseline, 2. Intervention: any school-, home-, or community based physical activity intervention or laboratory based exercise intervention, 3. Outcomes: changes in cognitive function or indicators of global well-/ill being; mediators: neurobiological, psychosocial and behavioral mechanisms, 4. Design: experimental or quasi-experimental studies of at least 1 week in duration, 5. Language: -, 6. Search: inception-July 2015, 7. Excluded: - | supplementary file | PubMed, PsycINFO, SCOPUS, Ovid Medline, SportDiscus, and Embase | Up to July 2015 |
| Martin et al (2018)(11) | 1. Population: children and adolescents with obesity or overweight (318 years), attending (pre)school, 2. Intervention: interventions to increase PA, 3. Outcomes: School achievement (excl parent and participant reported data), cognitive function using validated tests, 4. Design: RCT (incl cluster and quasi-RCT with or without cross-over), 5. Language: -, 6. Search: until February 2017, 7. Excluded: children with medical conditions known to affect weight status and academic achievement, PA intervention programms without a stated intention to prevent or treat childhood obesity, if adiposity was included as a covariate only. | In supplementary | "For this update, we searched 17 databases and two trials registers listed below in February 2017. 1 Cochrane Central Register of Controlled Trials (CENTRAL; 2017, Issue 1) in the Cochrane Library, which includes the Cochrane Developmental, Psychosocial and Learning Problems Specialised Register (searched 2 February 2017), 2 Ovid MEDLINE (1946 to January Week 4 2017), 3 Ovid MEDLINE E-PUB (searched 2 February 2017), 4 Ovid MEDLINE In-P (searched 2 February 2017), 5 Embase Ovid (1974 to 2017 Week 05), 6 PsycINFO Ovid (1806 to January Week 5 2017), 7 CINAHL Plus EBSCOhost (Cumulative Index to Nursing, and Allied Health Literature; 1937 to 3 February 2017), 8 ERIC EBSCOhost (Education Resources Information Center; 1966 to 3 February 2017), 9 SPORTDiscus EBSCOhost (1980 to 6 February 2017), 10 IBSS ProQuest (International Bibliography of Social Science; 1951 to 3 February 2017), 11 Conference Proceedings Citation Indexes (CPCI; 1990 to 2, February 2017), 12 Cochrane Database of Systematic Reviews (CDSR; 2017, Issue 2) part of the Cochrane Library (searched 2 February 2017), 13 Database of Reviews of Effectiveness (DARE; 2015, Issue, 2) part of the Cochrane Library (searched 3 February 2017), 14 Database of Promoting Health Effectiveness Reviews, (DoPHER; searched 6 February 2017), 15 EPPI-Centre Database of Health Promotion Research (searched 6 February 2017), 16 Trials Register of Promoting Health Interventions TRoPHI; searched 6 February 2017), 17 Dissertations and Theses Global-ProQuest (searched 8 February 2017), 18 ISRCTN Registry (wwwisrctncom; searched 8 February2017 ), 19 WHO International Clinical Trials Registry Platform (WHO ICTRP: whoint/trialsearch; searched 8 February 2017)" | February 2017 |
| Martin and Murtagh (2017)(12) | 1. Population: School-aged children (5-18 years), including overweight, 2. Intervention: PA in a school classroom setting, teaching academic context using physically active methods, of at least 1 week duration, 3. Outcomes: proportion of class time in MVPA, duration of MVPA, learning outcomes (academic performance), facilitators of learning, and health outcomes. 4. Design: interventions, 5. Language: English only, 6. Search: January 1990 - March 2015, 7. Excluded: PA interventions for participants with specific illnesses or multifactorial diseases. | "The search terms used to search titles/abstracts were (classroom AND (physical activity OR exercise OR physical inactivity OR sedentary) AND (school)) The search terms were slightly modified for certain databases such as Google Scholar and PubMed where the search terms were classroom AND physical activity AND school | ERIC, PubMed, Google Scholar, Science Direct, Cochrane Library, and EMBASE)" | January 1990 - March 2015 |
| Mura et al (2015)(13) | 1. Population: healthy pupils (3-18 years), 2. Intervention: PA interventions carried out in school settings (classroom, schoolyard, school gym and after-school), 3. Outcomes: cognitive/academic outcomes, 4. Design: Experimental and quasi-experimental studies, 5. Language: -, 6. Search: January 1980-June 2014, 7. Excluded: unpublished papers, studies with a non-experimental design or carried out in a different setting than schools, targeted at disabled children, not including an actual PA intervention, based on acute PA. | "School-based intervention or school-based program or school or schoolchildren or children AND physical activity or exercise AND cognition or cognitive performance or cognitive tasks or attention or memory or concentration or executive functions, OR academic achievement or academic performance or academic attainment or reading or writing or mathematic or learning" | PubMed/Medline, Scopus and Google Scholar | 1980- June 2014 |
| Pucher, Boot, and Vries (2013)(14) | 6. Search: June 7, 2010, 7. Excluded if not one of the following: Design: RCT, quasi-experimental, pre-post controlled trial or cross-over controlled trial. Outcomes: individual school grades results of national test examinations or GPAc. Intervention: mental health intervention, social/emotional learning interventions, or interventions for specific target groups rather than general school population. If no full text was available or repeated reports of interventions reporting identical outcomes with identical measures on the same population | Provided in the appendix of the paper | PubMed and PsycINFO | June 7, 2010 |
| Singh et al (2019)(15) | 1. Population: apparently healthy children or adolescents, 2. Intervention: PA-related intervention studies, 3. Outcomes: at least one cognitive or academic performance assessment, 4. Design: intervention studies, 5. Language: English only, 6. Search: until September 2017, 7. Excluded: studies that focused on a clinical sample | More information is provided in the appendix of the paper. "(1) physical activity (eg, physical activity, exercise, physical fitness, and sport); (2) cognitive and academic performance (eg, academic achievement, cognitive performance, academic performance, and school learning); (3) age (eg, infant, child, adolescent, and 018 years old); (4) intervention studies with various study designs (eg, randomised controlled trials (RCTs), cluster randomised trials)" | PubMed, PsycINFO, Cochrane Central, Web of Science, ERIC, and SPORTDiscus | Until sept 2017 |
| Spruit et al (2016)(16) | 1. Population: mean age between 11 and 18 and range 10-21 years old, 2. Intervention: PA intervention with a considerable sports or (aerobic) exercise component, 3. Outcome: report on psychosocial outcomes (internalising problems, externalising problems, self-concept and academic performance), with sufficient statistical information to calculate an effect size, 4. Design: experimental, 5. Language: -, 6. Search: performed in August 2015, 7. Excluded: population with physical health issues (except obesity) | "The search string comprised three elements: a physical activity intervention element, an age element, and a psychosocial outcome element. For the physical activity intervention element, the following keywords were used: "physical activity intervention", "sport$*$ intervention", or "exercise intervention" For the age element, the following keywords were used: "youth", "adolescen$*$", or "child" For the psychosocial outcome element, the keywords "behavior$*$", "internali$*$", "anxiety", "depression", "externali$*$", "delinquency", "conduct", "ODD", "aggression", "behavio$*$ problem", "antisocial", "psychosocial", "emotion$*$", "mental", "psychiat$*$", "academic", "grades", "GPA", "self concept", or "self-esteem" were used" | Ovid, Google Scholar, EBSCOhost (including SPORTdiscus), Proquest, Web of Science, Picarta, Wiley Online Library, Wiley Cochrane Library, and ScienceDirect | August 2015 |
| Suarez-Manzano et al (2018)(17) | 1. Population: young people with ADHD, school and high-school students (6-18 years), 2. Intervention: -, 3. Outcomes: -, 4. Design: interventional, 5. Language: English only, 6. Search: January 2000-January 2017, 7. Excluded: - | "1) Physical activity (physical fitness, cardiovascular fitness, physical activity, physical education, fitness, exercise, physical exercise, acute exercise, chronic exercise, healthy exercise, aerobic exercise, resistance exercise, anaerobic exercise) 2) Attention deficit hyperactivity disorder (attention-deficit hyperactivity disorder, ADHD, attention deficit disorder, ADD) 3) Children and adolescents (children, childhood, school-age, youth, adolescents, teenagers, students, school, high school)" | PubMed, SPORTDiscus, Web of Science, ProQuest and SCOPUS | January 2000-January 2017 |
| Vazou et al (2019)(18) | 1. Population: Typically developing children and adolescents, 2. Intervention: chronic PA interventions, 3. Outcome: cognitive outcomes, 4. Design: Intervention studies with a comparison group, 5. Language: - , 6. Search: - , 7. Excluded: the intervention did not include PA, participants were adults or non-typically developing children, the design was correlational or cross-sectional, the treatment consisted of a single exercise bout, no control or comparison group, the outcome variable was academic achievement and no direct measure of cognitive function | keywords including: (children, youth, adolescents) and (cognition, EF, cognitive performance) and (exercise, PA) | PubMed, PsycINFO, Web of Knowledge, and Scopus | NA |
| Verburgh et al (2014)(19) | 1. Population: children (6-12 years), adolescents (1317 years) or young adults (1835 years) with a mean age below or equal to 30 years, 2. Intervention: acute or chronic physical exercise, 3. Outcomes: executive function, 4. Design: , 5. Language: English only, 6. Search: until 1 April 2012, 7. Excluded: if multiple studies were extracted using the same participants, only the study with the largest sample was included | "physical activity", "physical exercise", "training", "aerobic exercise", "executive functions", "children", "youth", "adolescence", "young adults" | PubMed (early 1800- 2012), EMBASE (1974- 2012) and SPORTDiscus (1830- 2012) | Up to 2012 |

**References**

1. Álvarez-Bueno C, Pesce C, Cavero-Redondo II, Sanchez-Lopez M, Garrido-Miguel M, Martinez-Vizcaino V, et al. Academic Achievement and Physical Activity: A Meta-analysis. Pediatrics. 2017;140(6):e20171498.

2. Álvarez-Bueno C, Pesce C, Cavero-Redondo I, Sánchez-López M, Martínez-Hortelano JA, Martínez-Vizcaíno V. The Effect of Physical Activity Interventions on Children’s Cognition and Metacognition: A Systematic Review and Meta-Analysis. J Am Acad Child Adolesc Psychiatry. 2017;56(9):729–38.

3. Bustamante EE, Williams CF, Davis CL. Physical Activity Interventions for Neurocognitive and Academic Performance in Overweight and Obese Youth. A Systematic Review. Pediatr Clin North Am. 2016;63(3):459–80.

4. de Greeff JW, Bosker RJ, Oosterlaan J, Visscher C, Hartman E. Effects of physical activity on executive functions, attention and academic performance in preadolescent children: a meta-analysis. J Sci Med Sport. 2018;21(5):501–7.

5. Gunnell KE, Poitras VJ, LeBlanc A, Schibli K, Barbeau K, Hedayati N, et al. Physical activity and brain structure, brain function, and cognition in children and youth: A systematic review of randomized controlled trials. Ment Health Phys Act. 2018;16:105–27.

6. Haapala E. Physical Activity, Academic Performance and Cognition in Children and Adolescents. A Systematic Review. Balt J Heal Phys Act. 2012;4(1):53–61.

7. Jackson WM, Davis N, Sands SA, Whittington RA, Sun LS. Physical Activity and Cognitive Development: A Meta-Analysis. J Neurosurg Anesthesiol. 2016;28(4):373–80.

8. Lees C, Hopkins J. Effect of aerobic exercise on cognition, academic achievement, and psychosocial function in children: A systematic review of randomized control trials. Prev Chronic Dis. 2013;10(10):1–8.

9. Li JW, O’Connor H, O’Dwyer N, Orr R. The effect of acute and chronic exercise on cognitive function and academic performance in adolescents: A systematic review. J Sci Med Sport. 2017;20(9):841–8.

10. Lubans D, Richards J, Hillman C, Faulkner G, Beauchamp M, Nilsson M, et al. Physical activity for cognitive and mental health in youth: A systematic review of mechanisms. Pediatrics. 2016;138(3).

11. Martin A, Booth JN, Laird Y, Sproule J, Reilly JJ, Saunders DH. Physical activity, diet and other behavioural interventions for improving cognition and school achievement in children and adolescents with obesity or overweight. Cochrane Database Syst Rev. 2018;3(3):CD009728.

12. Martin R, Murtagh EM. Effect of Active Lessons on Physical Activity, Academic, and Health Outcomes: A Systematic Review. Res Q Exerc Sport. 2017;88(2):149–68.

13. Mura G, Vellante M, Nardi AE, Machado S, Carta MG. Effects of school-based physical activity interventions on cognition and academic achievement: a systematic review. CNS Neurol Disord - Drug Targets. 2015;14(9):1194–208.

14. Pucher KK, Boot N m. w. m., de Vries NK. Systematic review: School health promotion interventions targeting physical activity and nutrition can improve academic performance in primary- and middle school children. Health Educ. 2013;113(5):372–91.

15. Singh AS, Saliasi E, Van Den Berg V, Uijtdewilligen L, De Groot RHM, Jolles J, et al. Effects of physical activity interventions on cognitive and academic performance in children and adolescents: A novel combination of a systematic review and recommendations from an expert panel. Br J Sports Med. 2019;53(10):640–7.

16. Spruit A, Assink M, van Vugt E, van der Put C, Stams GJ. The effects of physical activity interventions on psychosocial outcomes in adolescents: A meta-analytic review. Clin Psychol Rev. 2016;45:56–71.

17. Suarez-Manzano S, Ruiz-Ariza A, De La Torre-Cruz M, Martínez-López EJ. Acute and chronic effect of physical activity on cognition and behaviour in young people with ADHD: A systematic review of intervention studies. Res Dev Disabil. 2018;77:12–23.

18. Vazou S, Pesce C, Lakes K, Smiley-Oyen A. More than one road leads to Rome: A narrative review and meta-analysis of physical activity intervention effects on cognition in youth. Int J Sport Exerc Psychol. 2019;17(2):153–78.

19. Verburgh L, Königs M, Scherder EJAA, Oosterlaan J. Physical exercise and executive functions in preadolescent children, adolescents and young adults: a meta-analysis. Br J Sports Med. 2014;48(12):973–9.
