# Supplementary material for: A critical evaluation of systematic reviews assessing the effect of chronic physical activity on academic achievement, cognition and the brain in children and adolescents: a systematic review
Source: Int J Behav Nutr Phys Act. 2020 Jun 22;17:79. doi: 10.1186/s12966-020-00959-y (PMC7310146; doi:10.1186/s12966-020-00959-y)
Supplement: Supplementary file 5 — Additional file 5. Countries where PA interventions were conducted. [file 12966_2020_959_MOESM5_ESM.docx]

# S5. Countries where PA interventions were conducted

For each outcome, the total number of unique publications across all reviews were extracted. We subsequently used the summary tables of the systematic reviews to extract the countries where the studies were conducted and searched for the original articles if an overview of countries was not included. The number of publications per country were then graphically displayed for each outcome measure: academic outcomes (Figure 1), cognitive outcomes (Figure 2), and brain outcomes (Figure 3).


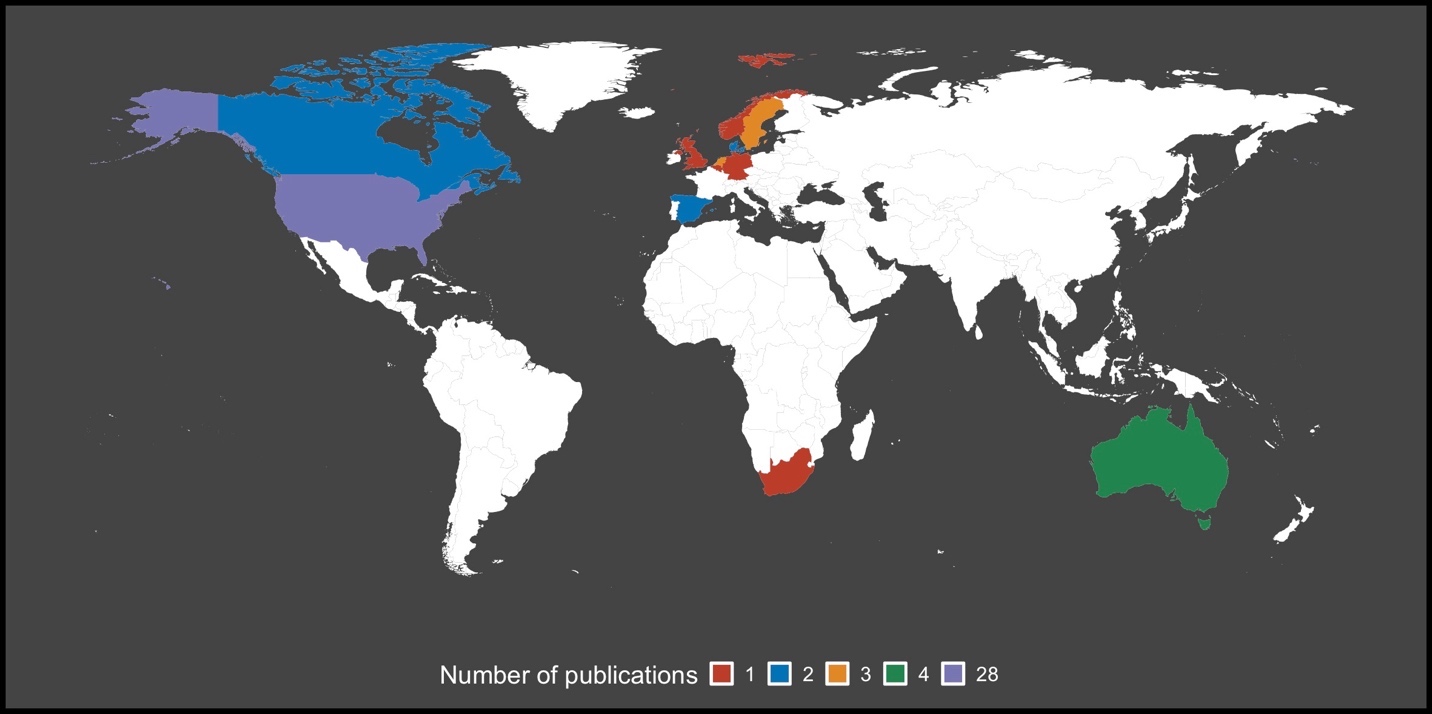


### Figure 1. Countries where PA interventions were conducted to assess effects on academic performance. Legend: the number of publications per country.

###
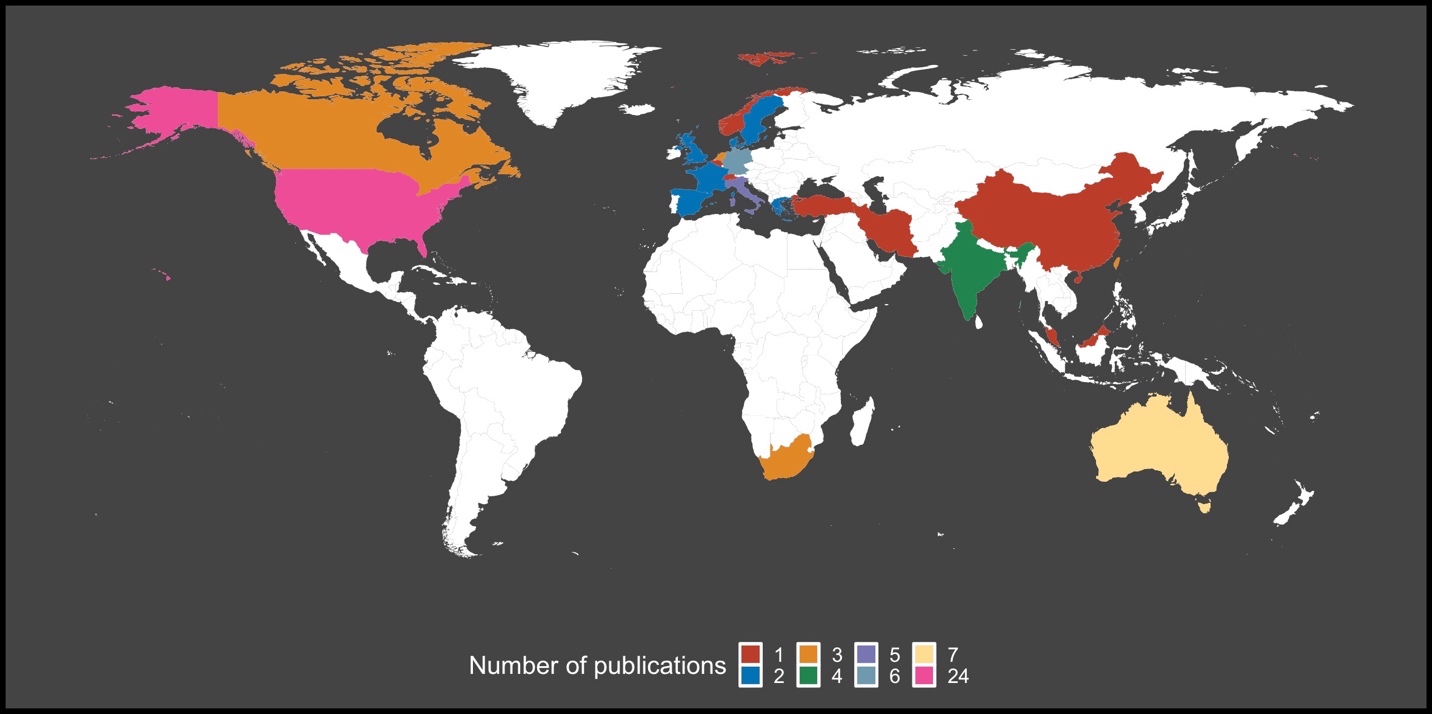
 Figure 2. Countries where PA interventions were conducted to assess effects on cognitive performance.

Legend: the number of publications per country.


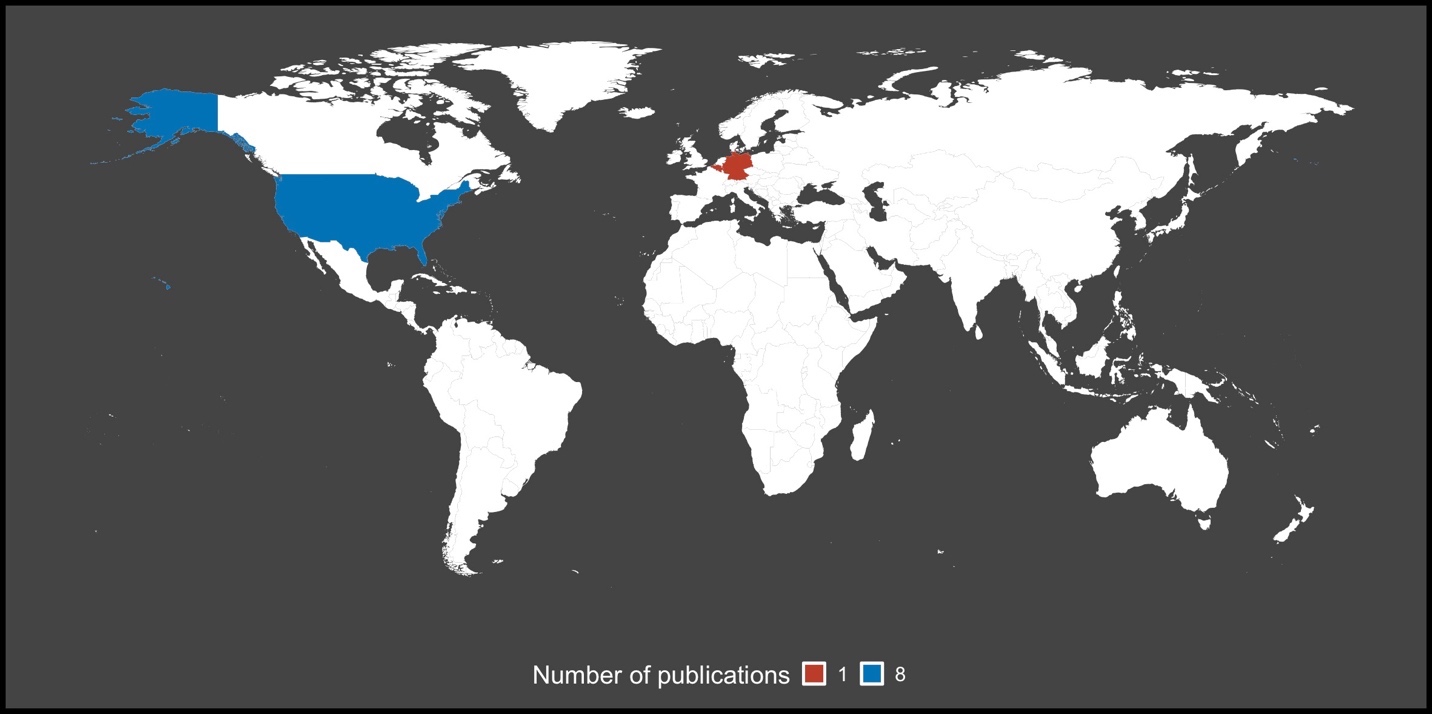


### Figure 3. Countries where PA interventions were conducted to assess effects brain outcomes.

Legend: the number of publications per country.
