# Supplementary material for: A critical evaluation of systematic reviews assessing the effect of chronic physical activity on academic achievement, cognition and the brain in children and adolescents: a systematic review
Source: Int J Behav Nutr Phys Act. 2020 Jun 22;17:79. doi: 10.1186/s12966-020-00959-y (PMC7310146; doi:10.1186/s12966-020-00959-y)
Supplement: Supplementary file 6 — Additional file 6. Quality assessment and bias tools from systematic reviews. [file 12966_2020_959_MOESM6_ESM.docx]

# S6. Quality assessment and bias tools

The majority of systematic reviews (15 out of 19) assessed the quality of the included studies. An overview of quality assessment tools used in the systematic reviews is provided in Table 1. Note, to preserve the interpretation of the tools by the authors, we took the descriptions of the tools from the manuscripts and made minor adjustments in order to limit the number of words.

### Table 1. Quality assessment of the primary studies

| **Authors** | **Tool**$\boldsymbol{}^{\boldsymbol{1}}$ | **Explanation by authors** | **Quality of the evidence** | **Quality (% high)**$\boldsymbol{}^{\boldsymbol{4}}$ |
| --- | --- | --- | --- | --- |
| Álvarez-Bueno et al (2017)(1) | Jadad Scale, EPHPP | **Jadad scale - RCTs:** Evaluation of 3 domains: randomization, double-blinding, and description of withdrawals and dropouts. Each item was scored as "1" or "0", with an additional point if randomization and double blinding were described in detail (range: 05 points) **EPHPP - non-RCT:** Seven domains were evaluated: selection bias, study design, confounders, blinding, data collection method, withdrawals, and dropouts. Each domain could be scored as strong, moderate, or weak. Studies were classified as strong (with no weak domains), moderate (with 1 weak domain), or weak (with 2 or more weak domains) | **Jadad scale (RCT):** Five points (1), four points (4), three points (10), two points (3), one point (0)  **EPHPP (non-RCT)** moderate (2), weak (6) | 19 |
| Álvarez-Bueno et al (2017)(2) | Jadad Scale, EPHPP | See description above | **Jadad scale (RCT):** five points (2), four points (6), three points (13), two points (9), one point (1)  **EPHPP (non-RCT):** moderate (1), weak (4) | 22 |
| Bustamante, Williams, and Davis (2016)(3) | Rigor score | Quality of the evidence for efficacy (3-points score): 1= low, 2 = medium, 3 = high | **RCT:** high (4), medium (3), low (2) **Non-RCT:** low (5) | 29 |
| De Greeff et al (2018)(4) | PEDro scale | The PEDro scale assesses randomization, blinding, intention-to-treat, between-group comparison and measures of variability (11 items). Scores on the PEDro scale range between 0 and 10 (one item pertains external validity and is not used). Adequate quality is defined as a study having an adequate generation of random sequence, concealment of allocation and blinding of outcome assessors, represented by a summary score of at least 5 points. | seven points (1), six points (3), five points (2), four points (4), three points (4), | 0 |
| Gunnell et al. (2018)(5) | Cochrane’s risk of bias tool | Evaluated each study for risk of bias caused by inadequate random sequence generation; allocation concealment; blinding of participants and personnel, or blinding of outcome assessment; incomplete outcome data; selective reporting; and other sources of bias. The risk of bias was ranked as low, high or unclear. | 66.7% low risk, 33.3% high risk | — |
| Haapala (2012)(6) | PEDro scale | A lower score (scale 0 to 11) indicates poorer methodological quality of the trials; a higher score is indicative of higher methodological quality. | nine points (1), eight points (1), five points (1) | 25 |
| Jackson et al (2016)(7) | Created their own scale | The risk of bias was assessed based on the quality of randomization, dropout rate, and the use of intention-to-treat principles, whether explicitly stated or inferred from the protocol | Low to moderate risk of bias, but lack of reporting of protocols of blinding and randomisation increased uncertainty | — |
| Lees and Hopkins (2013)(8) | Jadad scale | The Jadad Scale focuses on 3 criteria that have been shown to correlate with bias: randomization, blinding, and an account of all participants. The scale ranges from 0 to 5; studies less subject to bias score higher. | five points (0), four points (2), three points (0), two points (1), one point (0), zero points (1) | 0 |
| Li et al (2017)(9) | PEDro scale | The higher the PEDro score, the better the quality of the study. The quality of the studies is described as: 9-10 (excellent); 6-8 (good); 4-5 (fair); <3 (poor) | eight points (1), six points (1) | 0 |
| Lubans et al (2016)(10) | PEDro scale | This scale consists of 11 separate items representing different sources of potential bias in scientific research | five points (1), six points (3), nine points (2) | 33 |
| Martin et al (2018)(11) | Cochrane’s risk of bias tool | This included assessment of selection bias (random sequence allocation and allocation concealment), performance bias (blinding of participants and personnel), detection bias (blinding of outcome assessment), attrition bias (incomplete outcome data), reporting bias (selective reporting) and other sources of bias. The review authors judged the risk of bias as "high", "low" or "unclear", using the information provided | Not aggregated by authors | — |
| Martin and Murtagh (2017)(12) | Cochrane’s risk of bias tool | This seven-component rating scale assesses randomization, allocation blinding, blinding of participants and researchers, incomplete outcome data, discriminatory reporting, and other potential biases. High, unclear, or low risks of bias were awarded in each category. | Most of the studies have high risk of bias or have elements of the design that are unclear (e.g. random sequence generation, blinding of participants)$\dagger$ | NA |
| Mura et al (2015)(13) | NA | NA | NA | NA |
| Pucher, Boot, and Vries (2013)(14) | NA | NA | NA | NA |
| Singh et al (2019)(15) | adapted Quality assessment tool for quantitative studies | Assessed the methodological quality in seven dimensions: selection bias, study design, adjustment for potential confounders (e.g. age, gender, social economic status, and baseline academic/cognitive performance), data collection methods, blinding, withdrawals, and dropouts. High-quality studies: at least two strong and no weak dimensions, moderate-quality: less than two strong dimensions, but no more than one weak dimension, low-quality: more than one weak dimension. | High quality (11), moderate quality (29), weak (17) | 19 |
| Spruit et al (2016)(16) | NA | NA | NA | NA |
| Suarez-Manzano et al (2018)(17) | Standardized assessment lists | The list included six items on population, measurements, design, confounders and reporting of the results. Each item was rated as "2" (fully reported), "1" (moderately reported) or "0" (not reported or unclear). High quality (HQ): total score of nine or higher. Medium quality (MQ): total score of five to eight. Low quality (LQ): total score less than five. | All studies of high quality | 100 |
| Vazou et al (2019)(18) | NA | NA | NA | NA |
| Verburgh et al (2014)(19) | Newcastle-Ottowa scale | The scores ranged from 0 to 6 for crossover designs and from 0 to 7 for RCTs. Quantification of study quality according to the selection of individuals (2 points), comparability of experimental and control groups (2 points) and exposure of individuals to the condition assessed (3 points). Higher quality studies receive higher scores (0-7 points). | No significant association between study quality and effect sizes | — |

Abbreviations: EPHPP = Effective Public Health Practice Project, NA = not assessed / not available, PEDro scale = Physiotherapy Evidence Database Scale, RCT = randomised controlled trial.
^1^ References: Cochrane’s Risk of Bias tool (20) , EPHPP (21) , Jadad scale (22), Newcastle-Ottowa scale (23), PEDro scale (24), Standardized assessment lists (25,26), Quality assessment tool for quantitative studies (27).
^2^ Largely taken from manuscripts to ensure accurate description of the scales as intended by the authors.
^3^ The number of points / score is followed by the number of studies that achieved that score, i.e. score (no. studies).
^4^ The percentage of studies whose quality score fell within the top 25 % of scores on a given scale. Scores denoted with "—" could not be computed due to missing scores for individual studies or scales for which the scoring was unclear.
$\dagger$ Not specific to studies with cognitive-, academic- or brain outcomes.

**References**

1. Álvarez-Bueno C, Pesce C, Cavero-Redondo II, Sanchez-Lopez M, Garrido-Miguel M, Martinez-Vizcaino V, et al. Academic Achievement and Physical Activity: A Meta-analysis. Pediatrics. 2017;140(6):e20171498.

2. Álvarez-Bueno C, Pesce C, Cavero-Redondo I, Sánchez-López M, Martínez-Hortelano JA, Martínez-Vizcaíno V. The Effect of Physical Activity Interventions on Children’s Cognition and Metacognition: A Systematic Review and Meta-Analysis. J Am Acad Child Adolesc Psychiatry. 2017;56(9):729–38.

3. Bustamante EE, Williams CF, Davis CL. Physical Activity Interventions for Neurocognitive and Academic Performance in Overweight and Obese Youth. A Systematic Review. Pediatr Clin North Am. 2016;63(3):459–80.

4. de Greeff JW, Bosker RJ, Oosterlaan J, Visscher C, Hartman E. Effects of physical activity on executive functions, attention and academic performance in preadolescent children: a meta-analysis. J Sci Med Sport. 2018;21(5):501–7.

5. Gunnell KE, Poitras VJ, LeBlanc A, Schibli K, Barbeau K, Hedayati N, et al. Physical activity and brain structure, brain function, and cognition in children and youth: A systematic review of randomized controlled trials. Ment Health Phys Act. 2018;16:105–27.

6. Haapala E. Physical Activity, Academic Performance and Cognition in Children and Adolescents. A Systematic Review. Balt J Heal Phys Act. 2012;4(1):53–61.

7. Jackson WM, Davis N, Sands SA, Whittington RA, Sun LS. Physical Activity and Cognitive Development: A Meta-Analysis. J Neurosurg Anesthesiol. 2016;28(4):373–80.

8. Lees C, Hopkins J. Effect of aerobic exercise on cognition, academic achievement, and psychosocial function in children: A systematic review of randomized control trials. Prev Chronic Dis. 2013;10(10):1–8.

9. Li JW, O’Connor H, O’Dwyer N, Orr R. The effect of acute and chronic exercise on cognitive function and academic performance in adolescents: A systematic review. J Sci Med Sport. 2017;20(9):841–8.

10. Lubans D, Richards J, Hillman C, Faulkner G, Beauchamp M, Nilsson M, et al. Physical activity for cognitive and mental health in youth: A systematic review of mechanisms. Pediatrics. 2016;138(3).

11. Martin A, Booth JN, Laird Y, Sproule J, Reilly JJ, Saunders DH. Physical activity, diet and other behavioural interventions for improving cognition and school achievement in children and adolescents with obesity or overweight. Cochrane Database Syst Rev. 2018;3(3):CD009728.

12. Martin R, Murtagh EM. Effect of Active Lessons on Physical Activity, Academic, and Health Outcomes: A Systematic Review. Res Q Exerc Sport. 2017;88(2):149–68.

13. Mura G, Vellante M, Nardi AE, Machado S, Carta MG. Effects of school-based physical activity interventions on cognition and academic achievement: a systematic review. CNS Neurol Disord - Drug Targets. 2015;14(9):1194–208.

14. Pucher KK, Boot N m. w. m., de Vries NK. Systematic review: School health promotion interventions targeting physical activity and nutrition can improve academic performance in primary- and middle school children. Health Educ. 2013;113(5):372–91.

15. Singh AS, Saliasi E, Van Den Berg V, Uijtdewilligen L, De Groot RHM, Jolles J, et al. Effects of physical activity interventions on cognitive and academic performance in children and adolescents: A novel combination of a systematic review and recommendations from an expert panel. Br J Sports Med. 2019;53(10):640–7.

16. Spruit A, Assink M, van Vugt E, van der Put C, Stams GJ. The effects of physical activity interventions on psychosocial outcomes in adolescents: A meta-analytic review. Clin Psychol Rev. 2016;45:56–71.

17. Suarez-Manzano S, Ruiz-Ariza A, De La Torre-Cruz M, Martínez-López EJ. Acute and chronic effect of physical activity on cognition and behaviour in young people with ADHD: A systematic review of intervention studies. Res Dev Disabil. 2018;77:12–23.

18. Vazou S, Pesce C, Lakes K, Smiley-Oyen A. More than one road leads to Rome: A narrative review and meta-analysis of physical activity intervention effects on cognition in youth. Int J Sport Exerc Psychol. 2019;17(2):153–78.

19. Verburgh L, Königs M, Scherder EJAA, Oosterlaan J. Physical exercise and executive functions in preadolescent children, adolescents and young adults: a meta-analysis. Br J Sports Med. 2014;48(12):973–9.

20. Higgins JPT, Altman DG, Gøtzsche PC, Jüni P, Moher D, Oxman AD, et al. The Cochrane Collaboration’s tool for assessing risk of bias in randomised trials. BMJ. 2011;343(7829):1–9.

21. Armijo-Olivo S, Stiles CR, Hagen NA, Biondo PD, Cummings GG. Assessment of study quality for systematic reviews: A comparison of the Cochrane Collaboration Risk of Bias Tool and the Effective Public Health Practice Project Quality Assessment Tool: Methodological research. J Eval Clin Pract. 2012;18(1):12–8.

22. Jadad AR, Moore RA, Carroll D, Jenkinson C, Reynolds DJM, Gavaghan DJ, et al. Assessing the quality of reports of randomized clinical trials: Is blinding necessary? Control Clin Trials. 1996;17(1):1–12.

23. Wells G, Shea B, O’Connell D, Peterson J, Welch V, Losos M, et al. The Newcastle-Ottawa Scale (NOS) for assessing the quality of nonrandomised studies in meta-analyses [Internet]. [cited 2019 Oct 27].

24. Maher CG, Sherrington C, Herbert RD, Moseley AM, Elkins M. Reliability of the PEDro Scale for Rating Quality of Randomized Controlled Trials. Phys Ther. 2003;83(8):713–21.

25. Ruiz-Ariza A, Grao-Cruces A, Marques De Loureiro NE, Martï¿½nez-Lï¿½pez EJ. Influence of physical fitness on cognitive and academic performance in adolescents: A systematic review from 2005–2015. Int Rev Sport Exerc Psychol. 2017;10(1):108–33.

26. Ruiz JR, Castro-Piñero J, Artero EG, Ortega FB, Sjöström M, Suni J, et al. Predictive validity of health-related fitness in youth: A systematic review. Br J Sports Med. 2009;43(12):909–23.

27. Thomas BH, Ciliska D, Dobbins M, Micucci S. A process for systematically reviewing the literature: Providing the research evidence for public health nursing interventions. Worldviews Evidence-Based Nurs. 2004;1(3):176–84.
