# Supplementary material for: A critical evaluation of systematic reviews assessing the effect of chronic physical activity on academic achievement, cognition and the brain in children and adolescents: a systematic review
Source: Int J Behav Nutr Phys Act. 2020 Jun 22;17:79. doi: 10.1186/s12966-020-00959-y (PMC7310146; doi:10.1186/s12966-020-00959-y)
Supplement: Supplementary file 7 — Additional file 7. Quality assessment AMSTAR-2. [file 12966_2020_959_MOESM7_ESM.docx]

# S7. Quality assessment: outcomes

### Table 1. AMSTAR-2 quality assessment

| **Review** | **1** | **2** | **3** | **4** | **5** | **6** | **7** | **8** | **9** | **10** | **11** | **12** | **13** | **14** | **15** | **16** | **Critical** | **Non-**  **critical** | **Total** | **Confidence**  **rating** |
| --- | --- | --- | --- | --- | --- | --- | --- | --- | --- | --- | --- | --- | --- | --- | --- | --- | --- | --- | --- | --- |
| Álvarez-Bueno et al (2017)(1) | ✓ | ✓ | ✘ | ◇ | ✓ | ✓ | ✘ | ✓ | ✓ | ✘ | ✓ | ✘ | ✘ | ✓ | ✓ | ✓ | 4.5 | 6 | 10.5 | Critically low |
| Álvarez-Bueno et al (2017)(2) | ✓ | ✓ | ✘ | ◇ | ✓ | ✓ | ✘ | ✓ | ✓ | ✘ | ✓ | ✘ | ✘ | ✓ | ✓ | ✓ | 4.5 | 6 | 10.5 | Critically low |
| Bustamante, Williams, and Davis (2016)(3) | ✓ | ✘ | ✘ | ◇ | ✘ | ✘ | ✘ | ✓ | ✘ | ✘ | ✘ | ✘ | ✓ | ✓ | ✘ | ✘ | 1.5 | 3 | 4.5 | Critically low |
| De Greeff et al (2018)(4) | ✓ | ✘ | ✓ | ◇ | ✘ | ✘ | ◇ | ✓ | ✓ | ✘ | ✓ | ✘ | ✘ | ✓ | ✓ | ✘ | 4 | 4 | 8 | Critically low |
| Gunnell et al. (2018)(5) | ✓ | ✓ | ✘ | ◇ | ✓ | ✓ | ◇ | ✓ | ✓ | ✘ | ✘ | ✘ | ✘ | ✓ | ✘ | ✘ | 3 | 5 | 8 | Critically low |
| Haapala (2012)(6) | ✓ | ✘ | ✘ | ◇ | ✘ | ✘ | ✘ | ✓ | ✓ | ✘ | ✘ | ✘ | ✘ | ✘ | ✘ | ✘ | 1.5 | 2 | 3.5 | Critically low |
| Jackson et al (2016)(7) | ✓ | ✘ | ✘ | ◇ | ✓ | ✘ | ✘ | ◇ | ◇ | ✘ | ✓ | ✘ | ✘ | ✓ | ✓ | ✘ | 3 | 3.5 | 6.5 | Critically low |
| Lees and Hopkins (2013)(8) | ✓ | ✘ | ✓ | ◇ | ✘ | ✘ | ✘ | ✘ | ✓ | ✘ | ✘ | ✘ | ✘ | ✓ | ✘ | ✘ | 1.5 | 3 | 4.5 | Critically low |
| Li et al (2017)(9) | ✓ | ✘ | ✘ | ◇ | ✓ | ✘ | ✘ | ✓ | ✓ | ✘ | ✘ | ✘ | ✘ | ✓ | ✘ | ✘ | 1.5 | 4 | 5.5 | Critically low |
| Lubans et al (2016)(10) | ✓ | ✘ | ✘ | ◇ | ✘ | ✘ | ✘ | ✓ | ✓ | ✘ | ✘ | ✘ | ✘ | ✓ | ✘ | ✓ | 1.5 | 4 | 5.5 | Critically low |
| Martin et al (2018)(11) | ✓ | ✓ | ✓ | ✓ | ✓ | ✓ | ✓ | ✓ | ✓ | ✓ | ✓ | ✓ | ✓ | ✓ | ✓ | ✓ | 7 | 9 | 16 | High |
| Martin and Murtagh (2017)(12) | ✓ | ✘ | ✘ | ◇ | ✓ | ✘ | ✘ | ✓ | ✓ | ✘ | ✘ | ✘ | ✘ | ✓ | ✘ | ✘ | 1.5 | 4 | 5.5 | Critically low |
| Mura et al (2015)(13) | ✓ | ✘ | ✘ | ◇ | ✘ | ✘ | ✘ | ✓ | ✘ | ✘ | ✘ | ✘ | ✘ | ✘ | ✘ | ✓ | 0.5 | 3 | 3.5 | Critically low |
| Pucher, Boot, and Vries (2013)(14) | ✓ | ✘ | ✓ | ◇ | ✓ | ✘ | ✘ | ✓ | ✘ | ✘ | ✘ | ✘ | ✘ | ✓ | ✘ | ✘ | 0.5 | 5 | 5.5 | Critically low |
| Singh et al (2019)(15) | ✓ | ✓ | ✓ | ◇ | ✓ | ✓ | ✘ | ✓ | ✓ | ✘ | ✘ | ✘ | ✓ | ✓ | ✘ | ✓ | 3.5 | 7 | 10.5 | Low |
| Spruit et al (2016)(16) | ✓ | ✘ | ✘ | ◇ | ✘ | ✘ | ✘ | ✘ | ✘ | ✘ | ✓ | ✘ | ✘ | ✓ | ✓ | ✘ | 2.5 | 2 | 4.5 | Critically low |
| Suarez-Manzano et al (2018)(17) | ✘ | ✘ | ✘ | ◇ | ✓ | ✓ | ✘ | ✘ | ✓ | ✘ | ✘ | ✘ | ✘ | ✘ | ✘ | ✓ | 1.5 | 3 | 4.5 | Critically low |
| Vazou et al (2019)(18) | ✓ | ✘ | ✘ | ◇ | ✘ | ✘ | ✘ | ✓ | ✘ | ✘ | ✘ | ✘ | ✘ | ✓ | ✘ | ✘ | 0.5 | 3 | 3.5 | Critically low |
| Verburgh et al (2014)(19) | ✓ | ✘ | ✘ | ◇ | ✘ | ✘ | ✘ | ✘ | ✓ | ✘ | ✓ | ✓ | ✘ | ✓ | ✓ | ✓ | 3.5 | 4 | 7.5 | Critically low |
| **Total (%)** | 18  (95) | 5  (26) | 5  (26) | 19  (100) | 10  (53) | 6  (32) | 3  (16) | 15  (79) | 14  (74) | 1  (5) | 7  (88) | 2  (25) | 3  (16) | 16  (84) | 7  (88) | 8  (42) |  |  |  |  |

**Legend:** ✓= Yes, ◇ = partial yes, ✘= no

**AMTAR-2 details** Details of the AMTAR-2 items are provided in (20) and a brief explanation is provided here. *1*: whether the research question and inclusion criteria include PICO components, *2*: whether the review methods were established prior to the review, *3*: explanation of selection of included study designs, *4*: whether a comprehensive search of the literature was performed, *5*: whether study selection was performed in duplicate, *6*: whether data extraction was performed in duplicate, *7*: whether a list of excluded studies and justification was provided, *8*: whether the characteristics of the included studies were provided, *9*: whether a satisfactory technique was used to assess risk of bias, *10*: whether authors reported on the sources of funding, *11*: whether the appropriate methods for combining findings were used in meta-analysis, *12*: whether the impact of risk of bias on results was assessed in meta-analysis, *13*: whether risk of bias was used interpreting / discussing results, *14*: whether a satisfactory explanation and discussion of heterogeneity was provided , *15*: whether publication bias was assessed and discussed, *16*: whether a conflict of interest statement was included

**Modified scoring** Several items contained sub-items that were not included in the majority of the reviews. We considered these sub-items important, but believed that the lack of reporting should not determine the total item score. These sub-items included: *1*: whether a comparator group was included, *2*: whether a justification of publication restrictions was provided, *9*: whether the risk of bias assessment questioned if results were selected from among multiple measurements or analyses of a specified outcome, *11*: whether separate summary statistics were reported for RCT and non-randomised studies. In particular, for *9*, if a validated risk of bias assessment was used, this item was answered with "Yes".

**References**

1. Álvarez-Bueno C, Pesce C, Cavero-Redondo II, Sanchez-Lopez M, Garrido-Miguel M, Martinez-Vizcaino V, et al. Academic Achievement and Physical Activity: A Meta-analysis. Pediatrics. 2017;140(6):e20171498.

2. Álvarez-Bueno C, Pesce C, Cavero-Redondo I, Sánchez-López M, Martínez-Hortelano JA, Martínez-Vizcaíno V. The Effect of Physical Activity Interventions on Children’s Cognition and Metacognition: A Systematic Review and Meta-Analysis. J Am Acad Child Adolesc Psychiatry. 2017;56(9):729–38.

3. Bustamante EE, Williams CF, Davis CL. Physical Activity Interventions for Neurocognitive and Academic Performance in Overweight and Obese Youth. A Systematic Review. Pediatr Clin North Am. 2016;63(3):459–80.

4. de Greeff JW, Bosker RJ, Oosterlaan J, Visscher C, Hartman E. Effects of physical activity on executive functions, attention and academic performance in preadolescent children: a meta-analysis. J Sci Med Sport. 2018;21(5):501–7.

5. Gunnell KE, Poitras VJ, LeBlanc A, Schibli K, Barbeau K, Hedayati N, et al. Physical activity and brain structure, brain function, and cognition in children and youth: A systematic review of randomized controlled trials. Ment Health Phys Act. 2018;16:105–27.

6. Haapala E. Physical Activity, Academic Performance and Cognition in Children and Adolescents. A Systematic Review. Balt J Heal Phys Act. 2012;4(1):53–61.

7. Jackson WM, Davis N, Sands SA, Whittington RA, Sun LS. Physical Activity and Cognitive Development: A Meta-Analysis. J Neurosurg Anesthesiol. 2016;28(4):373–80.

8. Lees C, Hopkins J. Effect of aerobic exercise on cognition, academic achievement, and psychosocial function in children: A systematic review of randomized control trials. Prev Chronic Dis. 2013;10(10):1–8.

9. Li JW, O’Connor H, O’Dwyer N, Orr R. The effect of acute and chronic exercise on cognitive function and academic performance in adolescents: A systematic review. J Sci Med Sport. 2017;20(9):841–8.

10. Lubans D, Richards J, Hillman C, Faulkner G, Beauchamp M, Nilsson M, et al. Physical activity for cognitive and mental health in youth: A systematic review of mechanisms. Pediatrics. 2016;138(3).

11. Martin A, Booth JN, Laird Y, Sproule J, Reilly JJ, Saunders DH. Physical activity, diet and other behavioural interventions for improving cognition and school achievement in children and adolescents with obesity or overweight. Cochrane Database Syst Rev. 2018;3(3):CD009728.

12. Martin R, Murtagh EM. Effect of Active Lessons on Physical Activity, Academic, and Health Outcomes: A Systematic Review. Res Q Exerc Sport. 2017;88(2):149–68.

13. Mura G, Vellante M, Nardi AE, Machado S, Carta MG. Effects of school-based physical activity interventions on cognition and academic achievement: a systematic review. CNS Neurol Disord - Drug Targets. 2015;14(9):1194–208.

14. Pucher KK, Boot N m. w. m., de Vries NK. Systematic review: School health promotion interventions targeting physical activity and nutrition can improve academic performance in primary- and middle school children. Health Educ. 2013;113(5):372–91.

15. Singh AS, Saliasi E, Van Den Berg V, Uijtdewilligen L, De Groot RHM, Jolles J, et al. Effects of physical activity interventions on cognitive and academic performance in children and adolescents: A novel combination of a systematic review and recommendations from an expert panel. Br J Sports Med. 2019;53(10):640–7.

16. Spruit A, Assink M, van Vugt E, van der Put C, Stams GJ. The effects of physical activity interventions on psychosocial outcomes in adolescents: A meta-analytic review. Clin Psychol Rev. 2016;45:56–71.

17. Suarez-Manzano S, Ruiz-Ariza A, De La Torre-Cruz M, Martínez-López EJ. Acute and chronic effect of physical activity on cognition and behaviour in young people with ADHD: A systematic review of intervention studies. Res Dev Disabil. 2018;77:12–23.

18. Vazou S, Pesce C, Lakes K, Smiley-Oyen A. More than one road leads to Rome: A narrative review and meta-analysis of physical activity intervention effects on cognition in youth. Int J Sport Exerc Psychol. 2019;17(2):153–78.

19. Verburgh L, Königs M, Scherder EJAA, Oosterlaan J. Physical exercise and executive functions in preadolescent children, adolescents and young adults: a meta-analysis. Br J Sports Med. 2014;48(12):973–9.

20. Shea BJ, Reeves BC, Wells G, Thuku M, Hamel C, Moran J, et al. AMSTAR 2: A critical appraisal tool for systematic reviews that include randomised or non-randomised studies of healthcare interventions, or both. BMJ. 2017;358:1–9.
