# Supplementary material for: A critical evaluation of systematic reviews assessing the effect of chronic physical activity on academic achievement, cognition and the brain in children and adolescents: a systematic review
Source: Int J Behav Nutr Phys Act. 2020 Jun 22;17:79. doi: 10.1186/s12966-020-00959-y (PMC7310146; doi:10.1186/s12966-020-00959-y)
Supplement: Supplementary file 8 — Additional file 8. Overlap of primary studies across reviews. [file 12966_2020_959_MOESM8_ESM.docx]

# S8. Overlap of primary studies across reviews

Intersection plots were used to display whether primary studies were present in multiple systematic reviews. An intersection plot was created for cognitive-, academic- and brain outcomes (Figures 1 – 3). These plots can be interpreted as follows:

- *Left bar plot*: This bar plot contains the number of studies included in each systematic review, the name of which is displayed at the right side of the barplot
- *Middle circle plot*: This plot shows the intersections, i.e. whether a study was included in one or multiple systematic reviews. A closed dark blue circle denotes that a study was present in a systematic review and vertical lines connecting circles refer to the presence of that study in multiple reviews.
- *Top bar plot*: This bar plot shows the frequency of each intersection. That is, multiple studies can be included in the same set of multiple reviews, which results in a greater number of intersections. The cumulative frequency of intersections is equal to the total number of studies across reviews. Here, a value of one corresponds to a single study.

For each intersection plot of cognitive and academic outcomes, we extracted the studies that were present in five or more systematic reviews. For the intersection plot of brain outcomes, we extracted the studies that were present in all three systematic reviews.

The presence or absence of overlap of primary studies across reviews may help interpret the consistency of reviews’ conclusions. That is, consistency among reviews in their conclusions could be due to reviews being based on the same / similar set of primary research papers, whereas differences between review’s conclusions may be due to a non-overlapping set of primary studies being included.

## S8.1. Academic outcomes

Five primary studies have been included in at least five systematic reviews: Ahamed et al (2007)(1), Ardoy et al (2014)(2), Davis et al (2011)(3), Donnelly et al (2009)(4), Reed et al (2010)(5).


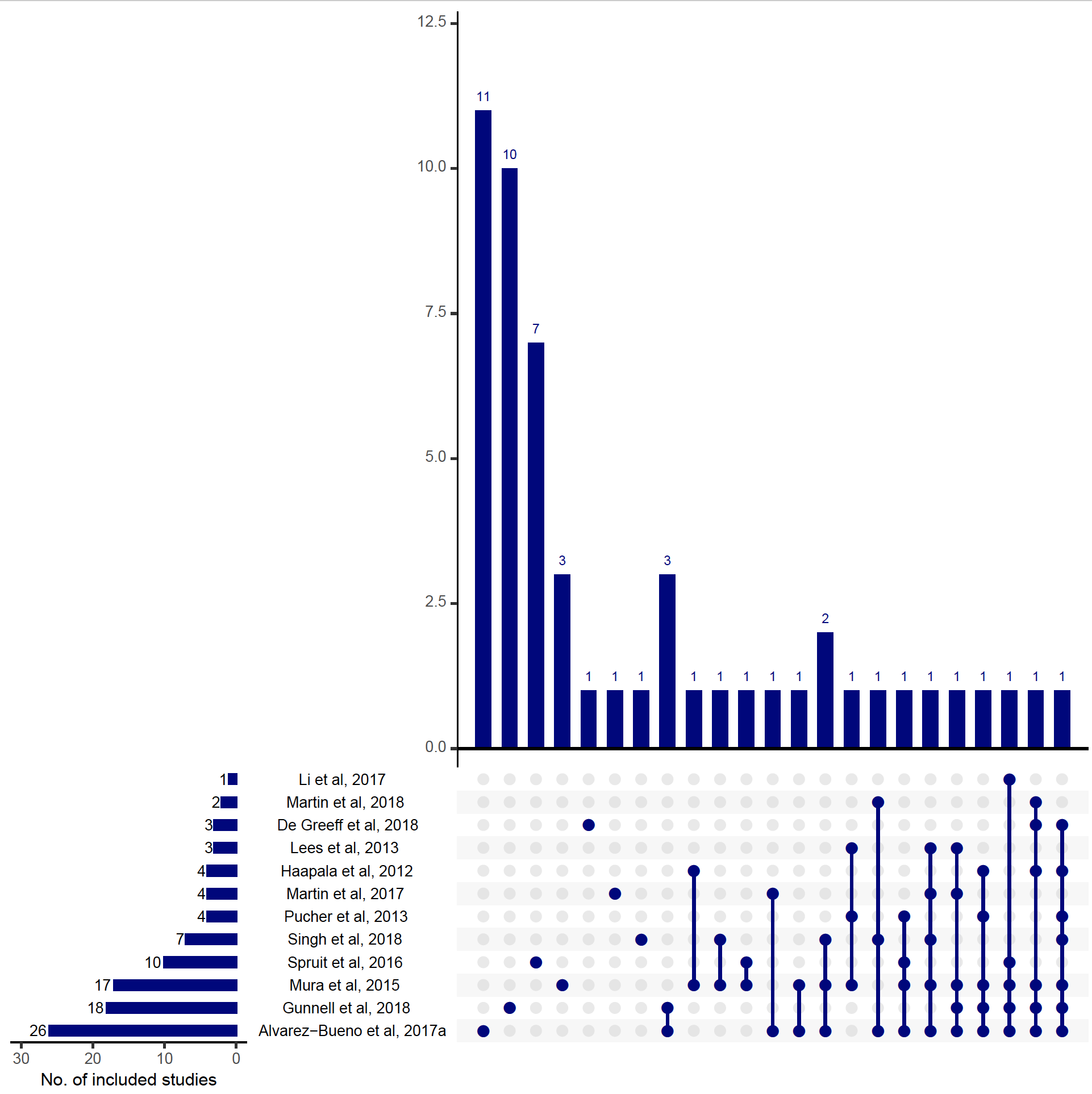


### Figure 1. Studies with academic outcomes included in one or multiple systematic reviews.

The barplot on the left displays the number of studies included in each systematic review. The bars and bullets at the right show how many of the unique publications (bars) were included in one or more systematic reviews (bullets). For example, the systematic review by Gunnell et al (2018)(6) included 10 studies that were unique to this systematic review (second bar, top bar-plot and single dot underneath it), one study that was included in six other systematic reviews (last bar, top bar plot and dot connected to six other studies beneath it).

## S8.2. Cognitive outcomes

Eight primary studies were included in at least five systematic reviews: Crova et al (2014)(7), Davis et al (2011)(3), Fisher et al (2011)(8), Gallotta et al (2015)(9), Hillman et al (2014)(10), Kamijo et al (2011)(11), Krafft et al (2014)(12), Schmidt et al (2015)(13).


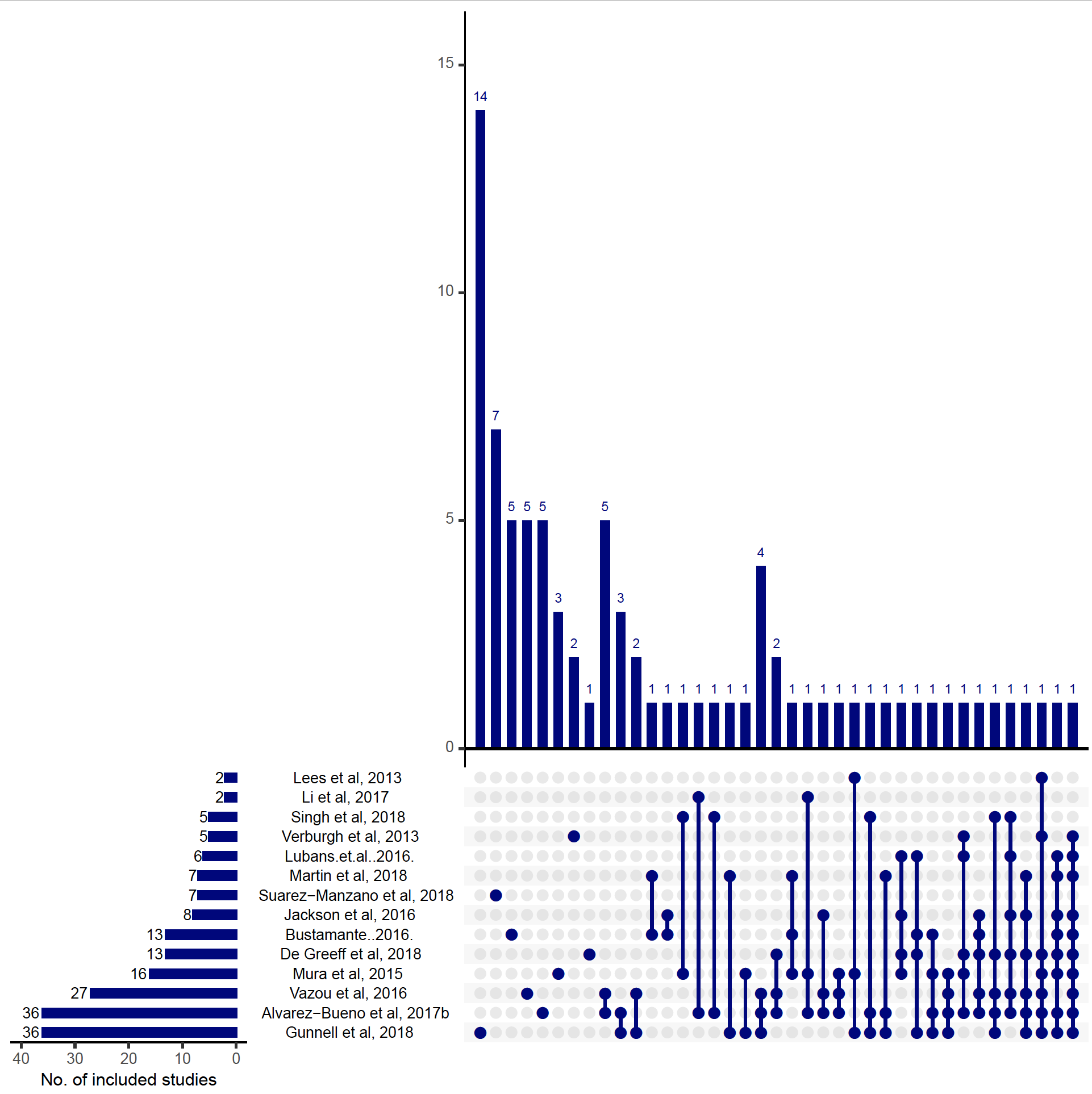


### Figure 2. Studies with cognitive outcomes included in one or multiple systematic reviews.

The barplot on the left displays the number of studies included in each systematic review. The bars and bullets at the right show how many of the unique publications (bars) were included in one or more systematic reviews (bullets). For example, the systematic review by Gunnell et al (2018)(6) included fourteen studies that were unique to this systematic review (first bar, top bar-plot and single dot underneath it), and one study that was included in nine other systematic reviews (last bar, top bar plot and dot connected to nine other studies beneath it).

## S8.3. Brain outcomes

Three primary studies were included in all three systematic reviews: Krafft et al (2014)(14), Krafft et al (2014)(12), Davis et al (2011)(3).


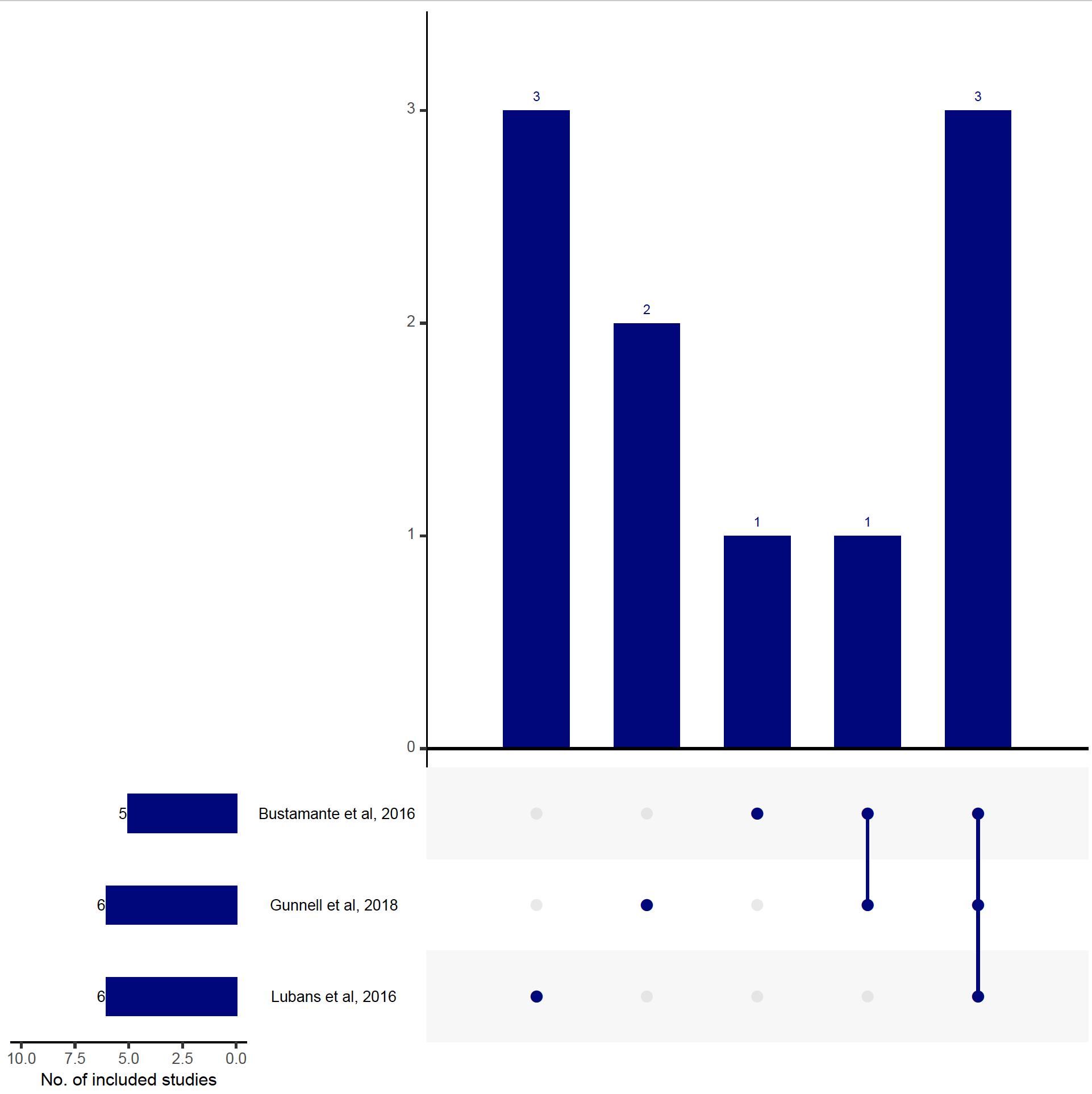


### Figure 3. Studies with brain outcomes included in one or multiple systematic reviews.

The barplot on the left displays the number of studies included in each systematic review. The bars and bullets at the right show how many of the unique publications (bars) were included in one or more systematic reviews (bullets). For example, the systematic review by Lubans et al (2016)(15) included three studies that were unique to this systematic review (second bar, top bar-plot and single dot underneath it), and one study that was included in two other systematic reviews (last bar, top bar plot and dot connected to two other studies beneath it).

**References**

1. Ahamed Y, Macdonald H, Reed K, Naylor PJ, Liu-Ambrose T, Mckay H. School-based physical activity does not compromise children’s academic performance. Med Sci Sports Exerc. 2007;39(2):371–6.

2. Ardoy DN, Castillo R, Ruiz JR, Ortega FB. A Physical Education trial improves adolescents ’ cognitive performance and academic achievement : the EDUFIT study. Scand J Med Sci Sports. 2014;24:52–61.

3. Davis CL, Tomporowski PD, McDowell JE, Austin BP, Miller PH, Allison JD, et al. Exercises improves executive function and achievement and alters brain activation in overweight children A randomized controlled trial. Heal Psychol. 2011;30(1):91–8.

4. Donnelly JE, Greene JL, Gibson CA, Smith BK, Washburn RA, Sullivan DK, et al. Physical Activity Across the Curriculum (PAAC): a randomized controlled trial to promote physical activity and diminish overweight and obesity in elementary school children. Prev Med (Baltim). 2009;49(4):336–41.

5. Reed JA, Einstein G, Hahn E, Hooker SP, Gross VP, Kravitz J. Examining the impact of integrating physical activity on fluid intelligence and academic performance in an elementary school setting: A preliminary investigation. J Phys Act Heal. 2010;7(3):343–51.

6. Gunnell KE, Poitras VJ, LeBlanc A, Schibli K, Barbeau K, Hedayati N, et al. Physical activity and brain structure, brain function, and cognition in children and youth: A systematic review of randomized controlled trials. Ment Health Phys Act. 2018;16:105–27.

7. Crova C, Struzzolino I, Marchetti R, Masci I, Forte R, Pesce C, et al. Cognitively challenging physical activity benefits executive function in overweight children. J Sports Sci. 2014;32(3):201–11.

8. Fisher A, Boyle JMEE, Paton JY, Tomporowski P, Watson C, Mccoll JH, et al. Effects of a physical education intervention on cognitive function in young children: Randomized controlled pilot study. BMC Pediatr. 2011;11(1):97.

9. Gallotta MC, Emerenziani G Pietro, Iazzoni S, Meucci M, Baldari C, Guidetti L. Impacts of coordinative training on normal weight and overweight/obese children’s attentional performance. Front Hum Neurosci. 2015;9:1–9.

10. Hillman CH, Pontifex MB, Castelli DM, Khan NA, Raine LB, Scudder MR, et al. Effects of the FITKids Randomized Controlled Trial on Executive Control and Brain Function. Pediatrics. 2014;134(4):e1063–71.

11. Kamijo K, Pontifex MB, O’Leary KC, Scudder MR, Wu CT, Castelli DM, et al. The effects of an afterschool physical activity program on working memory in preadolescent children. Dev Sci. 2011;14(5):1046–58.

12. Krafft CE, Schwarz NF, Chi L, Weinberger AL, Schaeffer DJ, Pierce JE, et al. An 8-Month Randomized Controlled Exercise Trial Alters Brain Activation During Cognitive Tasks in Overweight Children. Obesity. 2014;22(1):232–42.

13. Schmidt M, Egger F, Roebers CM, Conzelmann A. Cognitively Engaging Chronic Physical Activity , But Not Aerobic Exercise , Affects Executive Functions in Primary School Children. J Sport Exerc Psychol. 2015;37:575–91.

14. Krafft CE, Schaeffer DJ, Schwarz NF, Chi L, Weinberger AL, Pierce JE, et al. Improved frontoparietal white matter integrity in overweight children is associated with attendance at an after-school exercise program. Dev Neurosci. 2014;36(1):1–9.

15. Lubans D, Richards J, Hillman C, Faulkner G, Beauchamp M. Physical Activity for Cognitive and Mental Health in Youth : A Systematic Review of Mechanisms. Pediatrics. 2016;138(3):e20161642.
