# Supplementary material for: A critical evaluation of systematic reviews assessing the effect of chronic physical activity on academic achievement, cognition and the brain in children and adolescents: a systematic review
Source: Int J Behav Nutr Phys Act. 2020 Jun 22;17:79. doi: 10.1186/s12966-020-00959-y (PMC7310146; doi:10.1186/s12966-020-00959-y)
Supplement: Supplementary file 9 — Additional file 9. Supplementary findings from meta-analyses. [file 12966_2020_959_MOESM9_ESM.docx]

# S9. Supplementary findings from meta-analyses

Eight systematic reviews included meta-analyses. These meta-analyses were commonly accompanied by sub-group analyses, sensitivity analyses, meta-regressions and tests of publication bias. The findings of these analyses for academic outcomes are presented in Table 1, and for cognitive outcomes in Table 2.

### Table 1. Supplementary information on meta-analyses on academic outcomes

| **Author** | **Methods** | **Sub-group analysis** | **Sensitivity analysis** | **Meta-regression** | **Publication bias** |
| --- | --- | --- | --- | --- | --- |
| **High-quality reviews** | | | | | |
| Martin et al (2018)(1) | Random-effects model | NA | Performed for high risk of bias (e.g. attrition) and cluster RCT designs. No change in conclusions | NA | Could not be assessed due to low number of studies |
| **Critically low-quality reviews** | | | | | |
| Alvarez-Bueno et al (2017)(2) | Random-effects model, based on the DerSimonian and Laird method | *School time*  (1) curricular PE: maths (0.16 [0.00,0.32], reading (0.21 [0.05, 0.37]), composite scores (0.30 [0.03, 0.56]),  (2) Integrated or extracurricular PA: maths (0.29 [0.07, 0.51]) | Modification of language, reading and composite score ES upon removal of studies.  Removal of overweight/obese studies: maths (0.21 [0.09,0.34]), reading (0.16 [0.03,0.28]).  Removal of low quality (EPHPP) studies: maths (0.12 [0.03,0.18], $I^{2}$=19.4%), reading (0.13 [0.03,0.23], $I^{2}$=21%) | No associations with age of length of the intervention | For sub-group analysis of composite scores only (Egger’s regression asymmetry test) |
| De Greeff et al (2018)(3) | Random-effects model | *Type of PA:*  Aerobic: 0.29 (0.13,0.45), $I^{2}$ = 43.23% (s=11, k=12)  Cognitively engaging PA: 0.53 (0.14,0.92), $I^{2}$=78.87% (s=5, k=6) | NA | Duration had no significant influence on the effect of PA interventions | No evidence (funnel plot, Rosenthal’s fail-safe N and Egger’s linear regression method) |
| Spruit et al (2016)(4) | Multilevel random effects model | **Moderator analyses:**  Type of academic achievement (i.e. language, science, overall): $n.s.$  Type of measure: stronger effects if academic achievement measured through grades  Study design (i.e. RCT or quasi-experimental): $n.s.$  Type of comparison: $n.s.$  Sample (ethnic minority or males): $n.s.$ Intervention characteristics (type, duration and frequency, type of PA, team or individual): $n.s.$ | NA | NA | No evidence for publication bias (trim and fill procedure) |

Abbreviations: $d$ = Cohen’s $d$, ES = effect size, $g$ = Hedges’$g$, k = number of comparisons, n = number of participants, NA = not assessed, PA = physical activity, RCT = randomised controlled-trial, s = study/studies

### Table 2. Supplementary information on meta-analyses on cognitive outcomes

| **Author** | **Methods** | **Sub-group analysis** | **Sensitivity analysis** | **Meta-regression** | **Publication bias** |
| --- | --- | --- | --- | --- | --- |
| **High-quality reviews** | | | | | |
| Martin et al (2018)(1) | Random-effects model | NA | Performed for high risk of bias (e.g. attrition) and cluster RCT designs. No change in conclusions | NA | Insufficient numbers of included studies |
| **Critically low-quality reviews** | | | | | |
| Alvarez-Bueno et al (2017)(5) | Random-effects model based on the Der Simonian and Laird method (Cohen’s $d$) | *School time*: (1) Curricular PE: non-executive cognitive functions (0.42 [0.14,0.70]); the selective attention/inhibition component of core executive functions (0.41 [0.15,0.67]; and higher-level executive functions (0.25 [0.06,0.43]), no influence on working memory or cognitive flexibility.  *Task characteristics:* Non-executive functions: enhanced PA programs: (0.21 [0.07,0.35]); Working memory: enhanced PA programs (0.28 [0.04,0.52]); Selective attention/inhibition: Enriched PA programs (0.49 [0.05,0.93]), Cognitive flexibility: none; Higher level EF: enhanced PA (0.21 [0.09,0.34]).  *Weight status:* Selective attention/inhibition (-0.02 [-0.22,0.17])  *Time / accuracy (inhibition / cognitive flexibility):* (1) studies that focused on accuracy only: 0.15 (0.01,0.29), $I^{2}$=54.9%, (2) studies that focused on time only: -0.25 (-0.45, -0.04), $I^{2}$=0%, (3) studies that focused on accuracy and time, the effect of accuracy: 0.29 (0.04,0.54), $I^{2}$=83.4%, (4) studies that focused on accuracy and time, the effect of reaction time: -0.05 (-0.20, 0.11), $I^{2}$=61.8% | Effect size for working memory was modified | Positive association of the effect of PA on non-executive functions with age. The effect of PA interventions on working memory was negatively associated with length of intervention. Quality of the study was not related to heterogeneity across studies. | Significant publication bias for subgroup analysis of working memory (Egger’s regression asymmetry test, trim-and-fill plot) |
| De Greeff et al (2018)(3) | Random-effects model (Hedges’ $g$) Mean ES was used to combine outcomes within a sub-domain (e.g. accuracy and reaction time) | *Type of PA:* Aerobic: 0.29 [0.13,0.45],$I^{2}$ = 43.23% (s=11, k=12); Cognitively engaging PA: 0.53 [0.14,0.92], $I^{2}$ = 78.87% (s=5, k=6).  *Duration:* n.s. | No | NA | No evidence (Funnel plot, Rosenthal’s fail-safe N, Egger’s linear regression method) |
| Jackson et al (2016)(6) | ANOVA (Cohen’s $d$) Combined measures of RT and accuracy using the Borenstein et al method. | NA | NA | NA | NA |
| Verburgh et al (2014)(7) | Fixed-effects model, or random-effects model (Cohen’s $d$) | NA | NA | Duration of exercise did not account for a significant proportion of the variance for the effects of chronic exercise on executive functions ($\beta$=-0.39, $p$=0.35) | No evidence for publication bias (Rosenthal’s fail-safe N, Egger’s regression asymmetry method, meta-regression to assess the relation between sample size and effect size) |
| Vazou et al (2019)(8) | Random-effects model (Hedges’ $g$) | **Qualitatively different PA interventions contrasted with comparison conditions** *Aerobic:* Aerobic PA in overweight/obese versus no-treatment: $g$=1.8 (s=1); Aerobic PA versus academic instruction: 0.57 (s=2); Aerobic PA versus traditional PE: -0.08 (s=2)  *Motor skill (s=2):* no pooled effect size could be calculated.  *Cognitively engaging PA (s=2):* Cognitively engaging PA (yoga) versus no treatment ($g$=0.54); Cognitively engaging PA (yoga) versus aerobic ($g$=5.41);  *Motor and aerobic (s=1):* compared with no treatment: ($g$=0.05) (s=1)  *Motor and cognitively engaging:* compared to academic instruction: $g$=0.18 (s=1), compared to traditional PE: 0.21 (s=4)  *Aerobic and cognitively engaging:* compared to no treatment: $g$=1.44 (s=1), compared to academic instruction: 0.69 (s=6), $I^{2}$=88%, compared to traditional PE: $g$=0.03 (s=1), compared to aerobic PA: $g$=0.17 (s=2)  *Aerobic, cognitively engaging and motor skill:* compared to traditional PE: $g$=0.26 (s=1) | Overall effect on cognitive function, after excluding the two potential outliers: 0.38, (0.22,0.53), $I^{2}$ = 80% (s=19) Comparing all PA to academic instruction after removing 1 study: 0.42 (0.28,0.56), $I^{2}$=42% (s=9) Different combinations of PA interventions compared to aerobic PA without one study: 0.18 (-0.08, 0.44), $I^{2}$=0% (s=3) | NA | NA |

Abbreviations: $d$ = Cohen’s $d$, ES = effect size, $g$ = Hedges’$g$, k = number of comparisons, n = number of participants, NA = not assessed, PA = physical activity, RCT = randomised controlled-trial, s = study/studies

**References**

1. Martin A, Booth JN, Laird Y, Sproule J, Reilly JJ, Saunders DH. Physical activity, diet and other behavioural interventions for improving cognition and school achievement in children and adolescents with obesity or overweight. Cochrane Database Syst Rev. 2018;3(3):CD009728.

2. Álvarez-Bueno C, Pesce C, Cavero-Redondo II, Sanchez-Lopez M, Garrido-Miguel M, Martinez-Vizcaino V, et al. Academic Achievement and Physical Activity: A Meta-analysis. Pediatrics. 2017;140(6):e20171498.

3. de Greeff JW, Bosker RJ, Oosterlaan J, Visscher C, Hartman E. Effects of physical activity on executive functions, attention and academic performance in preadolescent children: a meta-analysis. J Sci Med Sport. 2018;21(5):501–7.

4. Spruit A, Assink M, van Vugt E, van der Put C, Stams GJ. The effects of physical activity interventions on psychosocial outcomes in adolescents: A meta-analytic review. Clin Psychol Rev. 2016;45:56–71.

5. Álvarez-Bueno C, Pesce C, Cavero-Redondo I, Sánchez-López M, Martínez-Hortelano JA, Martínez-Vizcaíno V. The Effect of Physical Activity Interventions on Children’s Cognition and Metacognition: A Systematic Review and Meta-Analysis. J Am Acad Child Adolesc Psychiatry. 2017;56(9):729–38.

6. Jackson WM, Davis N, Sands SA, Whittington RA, Sun LS. Physical Activity and Cognitive Development: A Meta-Analysis. J Neurosurg Anesthesiol. 2016;28(4):373–80.

7. Verburgh L, Königs M, Scherder EJAA, Oosterlaan J. Physical exercise and executive functions in preadolescent children, adolescents and young adults: a meta-analysis. Br J Sports Med. 2014;48(12):973–9.

8. Vazou S, Pesce C, Lakes K, Smiley-Oyen A. More than one road leads to Rome: A narrative review and meta-analysis of physical activity intervention effects on cognition in youth. Int J Sport Exerc Psychol. 2019;17(2):153–78.
