# Supplementary material for: A critical evaluation of systematic reviews assessing the effect of chronic physical activity on academic achievement, cognition and the brain in children and adolescents: a systematic review
Source: Int J Behav Nutr Phys Act. 2020 Jun 22;17:79. doi: 10.1186/s12966-020-00959-y (PMC7310146; doi:10.1186/s12966-020-00959-y)
Supplement: Supplementary file 10 — Additional file 10. Limitations and recommendations of included reviews. [file 12966_2020_959_MOESM10_ESM.docx]

# S10. Limitations and recommendations of included reviews

Limitations and recommendations were extracted from the discussion sections of systematic reviews (Table 1).

### Table 1. Supplementary information limitations and recommendations across systematic reviews

| **Review** | **Limitations** | **Recommendations** |
| --- | --- | --- |
| Alvarez-Bueno et al (2017)(1) | - Scarcity of studies prevented analysis of which characteristics (enriched, enhanced etc.) of interventions are associated with greater effects - There may be publication bias; poor results are usually not published - Substantial variability in measurement of academic achievement - Only few studies examined after school PA interventions - Removal of poor quality studies modified the effect sizes for maths-related skills | - Need for PA after-school studies - Qualified professionals are essential - Research is needed to support which type of intervention is most appropriate for school time - Further studies are needed to elucidate how enriched PA programs could affect academic achievement |
| Alvarez-Bueno et al (2017)(2) | - Lack of information regarding design of interventions (assessments, compliance, methodology) - Possibility of publication bias - Meta-analysis: data manipulation was necessary and could have introduced bias - Less than 50% of studies included were of good quality - Various cognitive measurements were used - Length of interventions and age could influence estimates | - Describe in more detail the intervention and compliance - Include long-term follow-up assessments - Well-designed PA programs joining motor and cognitive stimulation should be complemented with the great variety of outdoor free-play experiences - Qualified professionals are essential in designing PA strategies |
| Bustamante et al (2016) | - Low rigor of studies - Quasi-experimental studies suffered of a lack of a control group and were confounded with other elements of the intervention | - More high-quality trials are needed, particularly for minority racial or ethnic groups - Investigate the qualitative aspects of PA on neurocognition - Translational work is needed to examine how best to integrate PA into the school day |
| De Greeff et al (2018)(3) | - Not all moderators may have been considered - Nearly all studies included were on MVPA - Relatively low number of studies per subdomain - Only studies published after 2000 were included | - Test different exercise intensities - Need to test for initial fitness levels - Need to focus on cognitively engaging PA |
| Gunnell et al (2018) (4) | - Excluded studies with multiple distinct behavioural components - Unintentional confounding within studies, resulting in confounded relationships between PA and brain health - Studies of low and high quality received the same weight - May have used a different coding system compared to other studies for categorisation of outcomes - Only healthy populations were included - Did not include grey literature - Excluded exergames or screen-based PA - May have missed on more recent publications - Did not include studies with unstandardized outcomes of brain health | - More research needed on brain function and structure - Explore the effects of exergaming on brain and cognition - Use Bayesian statistics to directly test the null hypotheses |
| Haapala et al (2012) (5) | - Studies lack data on whether cardiorespiratory fitness improved - Studies were of short intervention length | - Need for long term intervention studies |
| Jackson et al (2016)(6) | - Variety in exercise programs, with different types of activity (i.e. heterogeneity) - Variety of measures were used to examine executive function (heterogeneity) - Control groups will have likely varied in the amount of PA - Potential risk of publication bias given the absence of negative studies | - Need to examine other executive functions |
| Lees et al (2013)(7) | - Variability/heterogeneity in measurement tools, and some lacked validity - Small sample sizes across studies - Short follow-up times - Limited number of available studies; with potential for publication bias | - Need for rigorous trials with large sample sizes - Need for standardized interventions - Need for valid and reliable tools of measurement - Need for longer term follow-ups |
| Li et al (2017)(8) | - Great heterogeneity of cohorts - Unvalidated cognitive and academic assessments - Small sample sizes - Inadequate or ineffective dose of exercise could explain inconsistent effects of exercise - Exercise protocol and dose are poorly reported - There is a bias towards the early adolescent years - Cannot directly compare studies due to variability of control conditions, diversity in cognitive measures | - Need for clearly defined exercise protocols (including higher intensities) - Greater collaboration between exercise-and neuroscientists is needed - Need for consensus on the measurement of cognitive domains and academic performance |
| Lubans et al (2016)(9) | - Study heterogeneity limited conclusions on pathways - Existing studies have investigated different aspects of the brain using a variety of methods - Only few studies examined neurobiological mechanisms | - Blinding of assessors in data collection - Replicate findings of previous research and assess transferability into real-world settings - No studies targeted adolescents, hence future studies should examine mediators in this population - More research needed from across the age spectrum - Need to report standardized regression coefficients - Conduct mediation analyses using the conceptual framework provided in this review |
| Martin et al (2018)(10) | - Heterogeneity in measurement tools - Low numbers of studies resulted in no assessment of publication bias - Only few studies were on adolescents - The majority of studies were conducted in high income countries - Small samples with potentially low power - Different executive function metrics and categorisations may have an impact on the conclusions | - Need for community-based interventions with larger samples - Need for studies in adolescents - Need to examine the extent to which sex and ethnicity influence the effect of PA interventions on cognition and academic achievement - Research is needed in low and middle-income countries - Longer-term follow-up trials are needed - To include brain imaging techniques in future research |
| Martin et al (2017)(11) | - Implementation of the interventions varied (by teachers, researchers, specialist teachers) - Great variability between studies: sample size, intervention duration, training and resources provided, implementation personnel, use of theoretical frameworks and outcomes - All studies used different assessments - Variability in duration of intervention, frequency, resources etc - On primary school pupils only - Only studies published in English were included - Conclusions are based on a small number of studies - Few studies were based on a theoretical framework - Few studies provided details on the intervention and implementation - Most studies were carried out in the USA - Presence of publication bias | - Need for more robust research (e.g. RCTs) - Essential to evaluate teacher approval and student enjoyment with regard to PA interventions - Need to examine and develop specific guidelines for teachers on how to implement physically active lessons |
| Mura et al (2015)(12) | NA | NA |
| Pucher et al (2013)(13) | - Only few report on gender and sex effects - Only studies in North America - Little information on moderators - Lack of homogeneity of intervention studies - Limited number of studies - Findings are partly based on cross-over controlled trials | - Pay more attention to factors mediating the effects of interventions on academic performance - Interventions should use outcome measures that allow better understanding of the contributions of individual intervention elements to educational outcomes - Gender and SES interactions should be considered - Long-term evaluations are needed |
| Singh et al (2019)(14) | - Majority of studies were conducted in children 6-12 years - Comparison of different doses and types of PA within studies, did not include a true no PA-control - Publication bias: overestimation of the beneficial effect of PA on cognitive/academic performance - Discrepancies in outcome quality assessment between studies suggesting a degree of subjectivity | - Need for well-designed studies to provide insight into causality, mechanisms and moderators, as well as PA characteristics that benefit academic performance/cognition (i.e. high quality RCTs, with multiple intervention arms and non-active control group) - Need to understand PA effects at the cellular, functional and morphological level - Explore mediating role of psychological mediators and advance understanding of roles of social belonging and support - Need to distinguish between qualitative and quantitative characteristics of PA - Explore mediators and moderators - Need to examine the effets of limiting or interrupting sedentary behavior - Careful consideration of the contrast in PA between intervention and control groups - Need to monitor actual PA levels and consider compliance in analysis - Need for adequate sample sizes - Application of valid and reliable measures of cognitive performance using standardised and practical assessment tools. - Need to report effect sizes and confidence intervals |
| Spruit et al (2016)(15) | - Quality of some included studies was low, with small samples and not all the information to code moderators - None of the primary studies controlled for amount of PA of the intervention and comparison groups during leasure time - The meta-analysis was based on a small number of studies which could negatively influence generalisability | - More empirical research into effects of PA on psychosocial outcomes is desirable to assess when, how and why PA positively influence psychosocial outcomes in adolescents - Intervention studies with robust designs (RCT and large samples), with well-documented and well-described interventions are needed - Focus on the influence of contextual factors of PA interventions on psychosocial outcomes |
| Suarez-Manzano et al (2018)(16) | - Only few studies (25%) included confounders | - Need more research on acute and chronic effect on cognition and behavior in young people with ADHD |
| Vazou et al (2019)(17) | - High heterogeneity between studies - Performing a formal moderator analysis was not possible due to the small number of studies within the various categories - A limitation of the meta-analysis is that all of the cognitive outcomes were combined to have one effect size per study - It is important to emphasise that interpretation of the results from comparisons between different types of PA programmes should be conducted with caution due to the very small number of studies in many types of PA - Grouping was made based on the authors’ descriptions provided in each study, however, it is possible that different qualitative characteristics of PA were implemented in the intervention but were not fully described in the manuscript | - Need to account for qualitative types of PA on cognitive benefits - When comparing a PA programme with a specific dose and characteristics of exercise to another comparator, the selection of the comparator will impact the degree of effectiveness of the intervention. Small-to-medium differences between treatments that involve different types of PA should be taken into account in planning future investigations. - To reach adequate statistical power, trial designs need to target factors that strengthen effects and improve reliability of measurements - Studies need to compare cognitively engaging PA, motor skills combined with aerobic PA or all three to academic content - The majority of studies were in elementary school children, more studies in other age groups are needed - There were few studies in overweight and obesity populations, more studies in these groups are needed - Need to examine whether findings generalise to other cultures - More studies needed regarding which types of PA and what doses may be optimal for different age groups - Muscle-strengthening activities have been understudied - More research into summer programmes is needed - Number of qualitative characteristics (e.g. rhythm, balance etc) need to be further explored |
| Verburgh et al (2014)(18) | - Not possible to examine the role of exercise intensity on the effects of exercise on executive functions given that only 12/25 studies monitored heart rate - It may be that the interventions were not suitable in terms of intensity, frequency or duration to enhance executive functioning - No details on timing of assessment have been provided - Only five studies addressed chronic exercise, of which three in planning, therefore no conclusions can be drawn regarding different executive function domains | - Investigate whether chronic exercise shows effects on executive functions comparable to acute exercise - More research is needed to examine age related differences in effects of exercise on executive functions - Need to monitor heart rate to improve comparability between studies - Need for high quality RCTs manipulating intensity and duration |

# References

1. Álvarez-Bueno C, Pesce C, Cavero-Redondo II, Sanchez-Lopez M, Garrido-Miguel M, Martinez-Vizcaino V, et al. Academic Achievement and Physical Activity: A Meta-analysis. Pediatrics. 2017;140(6):e20171498.

2. Álvarez-Bueno C, Pesce C, Cavero-Redondo I, Sánchez-López M, Martínez-Hortelano JA, Martínez-Vizcaíno V. The Effect of Physical Activity Interventions on Children’s Cognition and Metacognition: A Systematic Review and Meta-Analysis. J Am Acad Child Adolesc Psychiatry. 2017;56(9):729–38.

3. de Greeff JW, Bosker RJ, Oosterlaan J, Visscher C, Hartman E. Effects of physical activity on executive functions, attention and academic performance in preadolescent children: a meta-analysis. J Sci Med Sport. 2018;21(5):501–7.

4. Gunnell KE, Poitras VJ, LeBlanc A, Schibli K, Barbeau K, Hedayati N, et al. Physical activity and brain structure, brain function, and cognition in children and youth: A systematic review of randomized controlled trials. Ment Health Phys Act. 2018;16:105–27.

5. Haapala E. Physical Activity, Academic Performance and Cognition in Children and Adolescents. A Systematic Review. Balt J Heal Phys Act. 2012;4(1):53–61.

6. Jackson WM, Davis N, Sands SA, Whittington RA, Sun LS. Physical Activity and Cognitive Development: A Meta-Analysis. J Neurosurg Anesthesiol. 2016;28(4):373–80.

7. Lees C, Hopkins J. Effect of aerobic exercise on cognition, academic achievement, and psychosocial function in children: A systematic review of randomized control trials. Prev Chronic Dis. 2013;10(10):1–8.

8. Li JW, O’Connor H, O’Dwyer N, Orr R. The effect of acute and chronic exercise on cognitive function and academic performance in adolescents: A systematic review. J Sci Med Sport. 2017;20(9):841–8.

9. Lubans D, Richards J, Hillman C, Faulkner G, Beauchamp M. Physical Activity for Cognitive and Mental Health in Youth : A Systematic Review of Mechanisms. Pediatrics. 2016;138(3):e20161642.

10. Martin A, Booth JN, Laird Y, Sproule J, Reilly JJ, Saunders DH. Physical activity, diet and other behavioural interventions for improving cognition and school achievement in children and adolescents with obesity or overweight. Cochrane Database Syst Rev. 2018;3(3):CD009728.

11. Martin R, Murtagh EM. Effect of Active Lessons on Physical Activity, Academic, and Health Outcomes: A Systematic Review. Res Q Exerc Sport. 2017;88(2):149–68.

12. Mura G, Vellante M, Nardi AE, Machado S, Carta MG. Effects of school-based physical activity interventions on cognition and academic achievement: a systematic review. CNS Neurol Disord - Drug Targets. 2015;14(9):1194–208.

13. Pucher KK, Boot N m. w. m., de Vries NK. Systematic review: School health promotion interventions targeting physical activity and nutrition can improve academic performance in primary- and middle school children. Health Educ. 2013;113(5):372–91.

14. Singh AS, Saliasi E, Van Den Berg V, Uijtdewilligen L, De Groot RHM, Jolles J, et al. Effects of physical activity interventions on cognitive and academic performance in children and adolescents: A novel combination of a systematic review and recommendations from an expert panel. Br J Sports Med. 2019;53(10):640–7.

15. Spruit A, Assink M, van Vugt E, van der Put C, Stams GJ. The effects of physical activity interventions on psychosocial outcomes in adolescents: A meta-analytic review. Clin Psychol Rev. 2016;45:56–71.

16. Suarez-Manzano S, Ruiz-Ariza A, De La Torre-Cruz M, Martínez-López EJ. Acute and chronic effect of physical activity on cognition and behaviour in young people with ADHD: A systematic review of intervention studies. Res Dev Disabil. 2018;77:12–23.

17. Vazou S, Pesce C, Lakes K, Smiley-Oyen A. More than one road leads to Rome: A narrative review and meta-analysis of physical activity intervention effects on cognition in youth. Int J Sport Exerc Psychol. 2019;17(2):153–78.

18. Verburgh L, Königs M, Scherder EJAA, Oosterlaan J. Physical exercise and executive functions in preadolescent children, adolescents and young adults: a meta-analysis. Br J Sports Med. 2014;48(12):973–9.
