# Supplementary material for: A critical evaluation of systematic reviews assessing the effect of chronic physical activity on academic achievement, cognition and the brain in children and adolescents: a systematic review
Source: Int J Behav Nutr Phys Act. 2020 Jun 22;17:79. doi: 10.1186/s12966-020-00959-y (PMC7310146; doi:10.1186/s12966-020-00959-y)
Supplement: Supplementary file 11 — Additional file 11. Studies included in PA committee reports. [file 12966_2020_959_MOESM11_ESM.docx]

# S11. Studies included in PA committee reports

The UK Expert Working Group Working Paper on children and young people (1) and the 2018 Physical Activity Guidelines Advisory Committee (2) each included a systematic search for systematic reviews that examined the effects of PA on cognitive and academic outcomes. The systematic reviews on which their conclusions were based can be found in Table 1, along with a list of reasons why a particular review was or was not included in the current systematic review of reviews.

### Table 1. Overview of reviews included in committee reports

| **Committee paper** | **Review** | **In- or excluded with reasons** |
| --- | --- | --- |
| US guidelines(2) | Bustamante et al (2016)(3) | Included in review |
| US guidelines(2) | Carson et al (2016)(4) | Different target population (early childhood) |
| US guidelines(2) | Donnelly et al (2016)(5) | Mixture of observational and interventional |
| US guidelines(2) | Janssen et al (2014)(6) | Full text not screened (and on acute PA) |
| US guidelines(2) | Esteban-Cornejo et al (2015)(7) | Mixture of observational and interventional |
| US guidelines(2) | Spruit et al (2016)(8) | Included in review |
| US guidelines(2) | Cerrillo-Urbana et al (2015)(9) | Mixture of acute and chronic |
| US guidelines(2) | Den Heijer et al (2017)(10) | Mixture of acute and chronic |
| US guidelines(2) | Tan et al (2016)(11) | Different target population |
| UK working group(1) | Chalkley et al (2015)(12) | Not part of the literature search (and no systematic review) |
| UK working group(1) | Singh et al (2019)(13) | Included in review |
| UK working group(1) | Li et al (2017)(14) | Included in review |

**References**

1. Jago R, Fairclough SJ, Mackintosh KA, Mccrorie P, Sebire SJ, Sherar LB, et al. Expert Working Group Working Paper Children and Young People UK physical activity guidelines: draft review and recommendations for Children and Young People [Internet]. 2018.

2. 2018 Physical Activity Guidelines Advisory Committee. 2018 Physical Activity Guidelines Advisory Committee Scientific Report [Internet]. Washington, DC; 2018.

3. Bustamante EE, Williams CF, Davis CL. Physical Activity Interventions for Neurocognitive and Academic Performance in Overweight and Obese Youth. A Systematic Review. Pediatr Clin North Am. 2016;63(3):459–80.

4. Carson V, Hunter S, Kuzik N, Wiebe SA, Spence JC, Friedman A, et al. Systematic review of physical activity and cognitive development in early childhood. J Sci Med Sport. 2016;19:573–8.

5. Donnelly JE, Hillman CH, Castelli D, Etnier JL, Lee S, Tomporowski P, et al. Physical activity, fitness, cognitive function, and academic achievement in children: a systematic review. Med Sci Sports Exerc. 2016;48(6):1197.

6. Janssen M, Toussaint HM, van Mechelen W, Verhagen EA. Effects of acute bouts of physical activity on children’s attention: a systematic review of the literature. Springerplus. 2014;3:410.

7. Esteban-Cornejo I, Tejero-Gonzalez CM, Sallis JF, Veiga OL. Physical activity and cognition in adolescents: A systematic review. J Sci Med Sport. 2015;18(5):534–9.

8. Spruit A, Assink M, van Vugt E, van der Put C, Stams GJ. The effects of physical activity interventions on psychosocial outcomes in adolescents: A meta-analytic review. Clin Psychol Rev. 2016;45:56–71.

9. Cerrillo-Urbina AJ, García-Hermoso A, Sánchez-López M, Pardo-Guijarro MJ, Santos Gómez JL, Martínez-Vizcaíno V. The effects of physical exercise in children with attention deficit hyperactivity disorder: A systematic review and meta-analysis of randomized control trials. Child Care Health Dev. 2015;41(6):779–88.

10. Den Heijer AE, Groen Y, Tucha L, Fuermaier ABM, Koerts J, Lange KW, et al. Sweat it out? The effects of physical exercise on cognition and behavior in children and adults with ADHD: a systematic literature review. J Neural Transm. 2017;124(S1):3–26.

11. Tan BWZ, Pooley JA, Speelman CP. A Meta-Analytic Review of the Efficacy of Physical Exercise Interventions on Cognition in Individuals with Autism Spectrum Disorder and ADHD. J Autism Dev Disord. 2016;46:3126–43.

12. Chalkley A, Milton K, Foster C. Change4Life evidence review: Rapid evidence review on the effect of physical activity participation among children aged 5-11 years. London; 2015.

13. Singh AS, Saliasi E, Van Den Berg V, Uijtdewilligen L, De Groot RHM, Jolles J, et al. Effects of physical activity interventions on cognitive and academic performance in children and adolescents: A novel combination of a systematic review and recommendations from an expert panel. Br J Sports Med. 2019;53(10):640–7.

14. Li JW, O’Connor H, O’Dwyer N, Orr R. The effect of acute and chronic exercise on cognitive function and academic performance in adolescents: A systematic review. J Sci Med Sport. 2017;20(9):841–8.
